# Enantioselective synthesis of *Iboga* alkaloids and vinblastine *via* rearrangements of quaternary ammoniums†
†Electronic supplementary information (ESI) available: Experimental procedures and characterisation data for all compounds are provided. CCDC 1433180, 1433182 and 1433188. For ESI and crystallographic data in CIF or other electronic format see DOI: 10.1039/c6sc00932h


**DOI:** 10.1039/c6sc00932h

**Published:** 2016-05-16

**Authors:** Yun Zhang, Yibin Xue, Gang Li, Haosen Yuan, Tuoping Luo

**Affiliations:** a Key Laboratory of Bioorganic Chemistry and Molecular Engineering of Ministry of Education , Beijing National Laboratory for Molecular Science (BNLMS) , College of Chemistry and Molecular Engineering , Peking-Tsinghua Center for Life Sciences , Academy for Advanced Interdisciplinary Studies , Peking University , Beijing 100871 , China . Email: tuopingluo@pku.edu.cn; b Laboratory of Chemical Genomics , School of Chemical Biology and Biotechnology , Peking University Shenzhen Graduate School , Shenzhen 518055 , China

## Abstract

An efficient and novel strategy for the enantioselective syntheses of various *iboga* alkaloids has been developed. The salient features include a gold-catalyzed oxidation of a terminal alkyne followed by cyclization, a Stevens rearrangement and a tandem sequence that combines the gold-catalyzed oxidation, cyclization and [1,2]-shift. The catharanthine analogs provided by our approach were further converted to the *vinca* alkaloid vinblastine and its analogs, which confirmed the remarkable sensitivity of the cytotoxicity to the C20′ substituent of vinblastine.

## Abstract 2

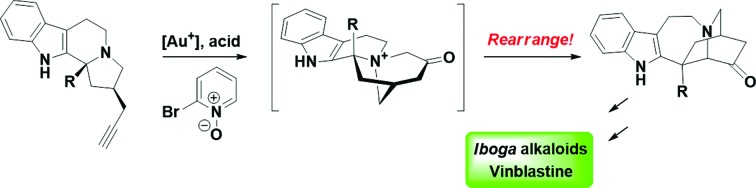
We present an efficient and unified strategy for the enantioselective syntheses of various *iboga* alkaloids and vinblastine, involving gold-catalyzed oxidation and Stevens rearrangement. New vinblastine analogs were prepared by our 10-step synthesis.

## Introduction

The total synthesis of complex natural product small molecules invites the examination of various methodologies in a complicated system, which at times reveals current limitations and inspires new advances.^1^ Importantly, total synthesis could also provide valuable analogs to explore the structure–activity relationships of targeted chemotypes.^2^ We have been interested in using rearrangement reactions that lead to dramatic changes in molecular skeletons to develop novel and efficient synthetic routes towards various biologically active natural products.^3^ Herein, we describe a concise and collective synthesis^4^ of *iboga* alkaloids and vinblastine which further substantiates these concepts.

**Fig. 1:**
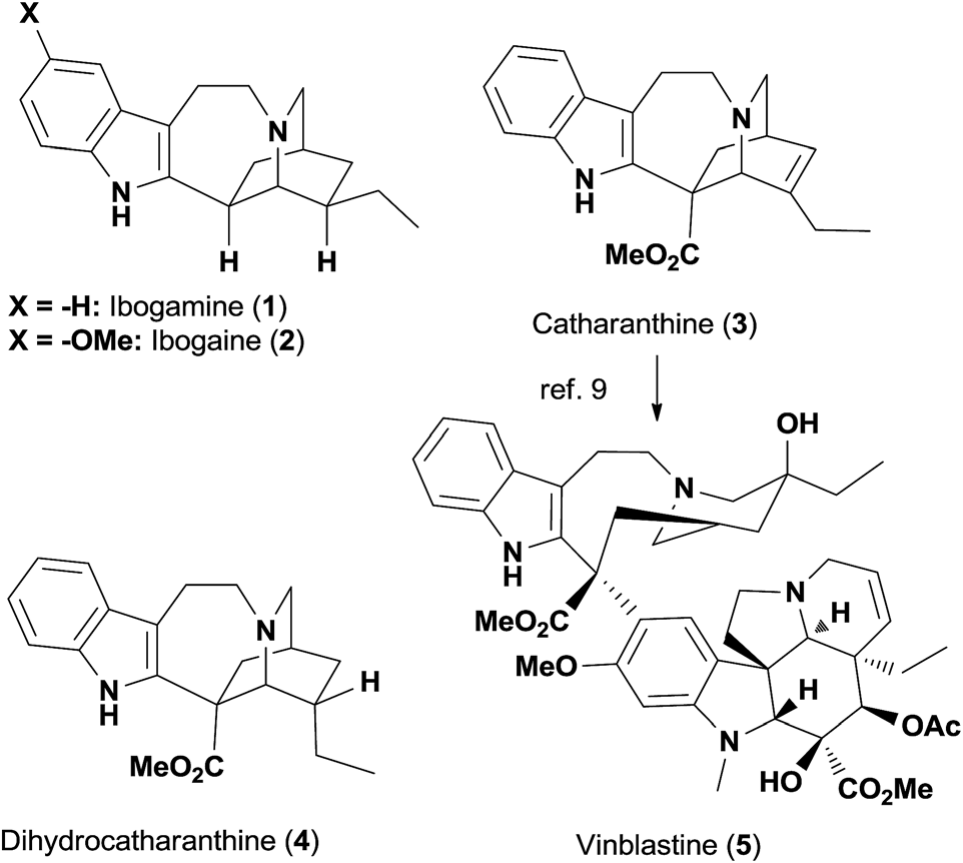
Representative *iboga* alkaloids and vinblastine.

The *iboga* alkaloid family of natural products comprises over 60 members of monoterpene indoles that share a common pentacyclic skeleton of ibogamine (Fig. 1).^5^ Among the various neurological activities of ibogamine (**1**) and ibogaine (**2**), the most exciting one is their capability to attenuate the addiction to a number of drugs, although the molecular mechanism of action remains largely elusive.^6^ Ibogaine, as the most abundant alkaloid in the root bark of the shrub *Tabernanthe iboga*, has even been studied in a clinical setting.^6^ Interestingly, both enantiomers of ibogamine are not only active in reducing the self-administration of cocaine and morphine in rats but are also devoid of tremorigenic activity—a side effect exhibited by ibogaine, which hence deserves further investigation.^7^ Catharanthine (**3**) and its derivative dihydrocatharanthine (**4**) have recently been identified as among the most potent TRPM8 antagonists and modulate cold-induced pain signals as well as mammalian thermoregulation.^8^ More importantly, the conversion of catharanthine to the potent anti-cancer drug vinblastine (**5**) *via* a one-pot procedure has boosted the value of this iboga alkaloid, and its derivatives have led to vinblastine analogs revealing insightful structure–activity relationships.^9^

Despite the variety of synthetic approaches towards different *iboga* alkaloids that have been reported, the enantioselective total syntheses remain relatively sparse.^10^–^13^ Since Trost's group published the elegant synthesis of enantioenriched ibogamine in 1978,^11^ the preparation of chiral isoquinuclidine fragments followed by the construction of a C2–C16 bond in the late stages (catharanthine numbering, throughout) has become the focus of asymmetric synthesis studies.^12^ The only two exceptions are the efficient syntheses of (–)-ibogamine and (–)-catharanthine by White's group and Oguri's group respectively, both employing the asymmetric Diels–Alder reaction.^13^ An alternative approach to prepare such a privileged skeleton, especially in an enantioselective manner, would be a valuable addition to current synthetic endeavours and more importantly, would enable flexible structural changes of this chemotype.

## Results and discussion

**Fig. 2:**
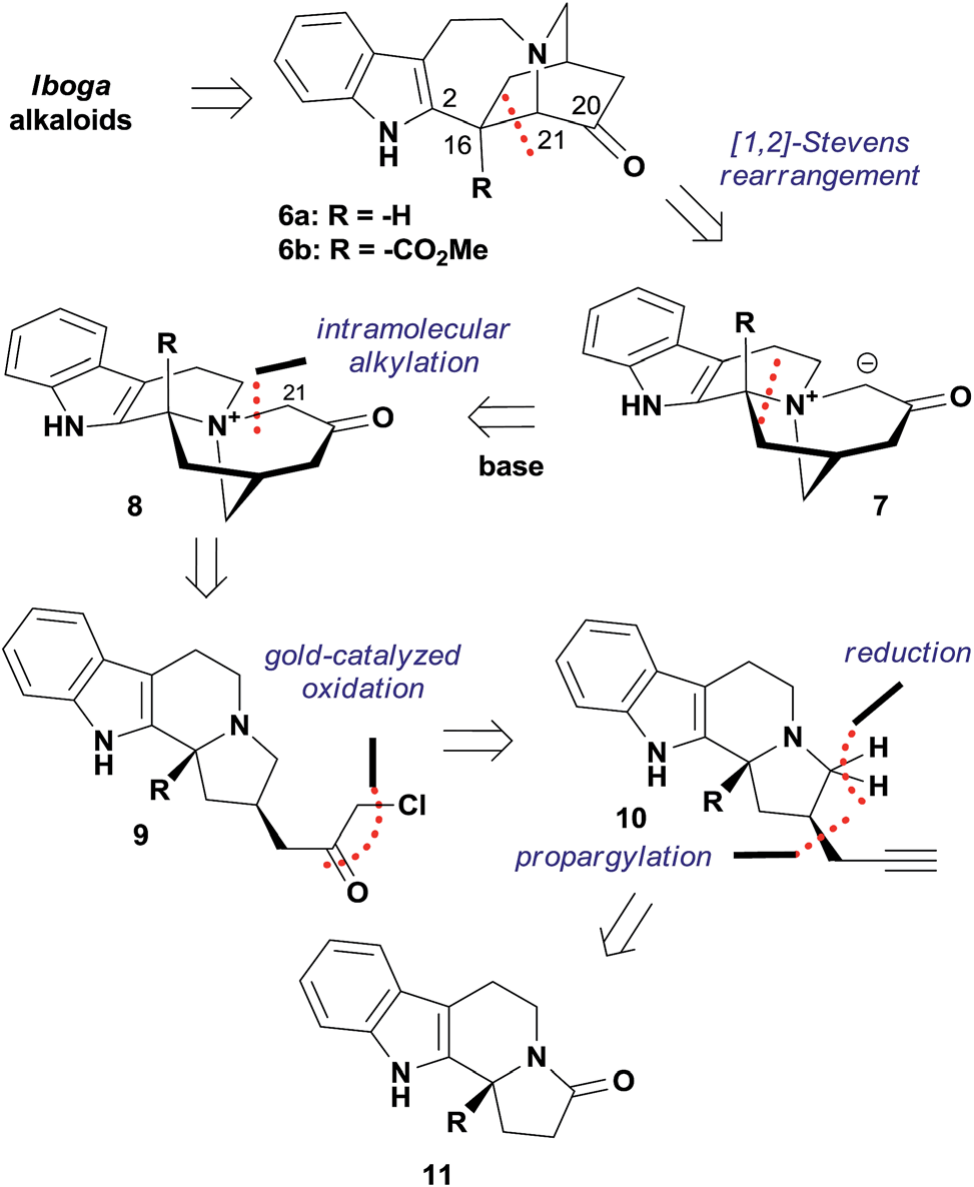
Retrosynthetic analysis of *iboga* alkaloids based on the [1,2]-Stevens rearrangement reaction.

While seeking a unified strategy to access *iboga* alkaloids with and without the methoxycarbonyl group at C16, we envisioned two late-stage intermediates **6a** and **6b** (Fig. 2). The C20 carbonyl group of **6** could be a versatile handle for the preparation of bioactive natural products and small-molecule probes. Inspired by the transannular cyclization accomplished by Kutney and co-workers,10*b* as well as recent advances in the fragmentation of the C16–C21 bond,^14^ we decided to explore the [1,2]-Stevens rearrangement of ammonium ylide **7** to construct the C16–C21 bond and give the structurally compact product **6**.^15^ Given that zwitterion **7** could be generated from the quaternary ammonium cation **8** upon treatment with base due to the enhanced acidity at C21, **6** would therefore be accessible from **8** in one step. This key precursor **8** could be prepared by intramolecular alkylation of the tertiary amine **9**, for which we hypothesized that the recently developed gold-catalyzed conversion of terminal alkynes to α-chloromethyl ketones could find application.^16^ Thus, the tertiary amine **10** became the precursor for **9**, which could be traced back to the known amide **11***via* propargylation and reduction.^17^,^18^

**Scheme 1:**
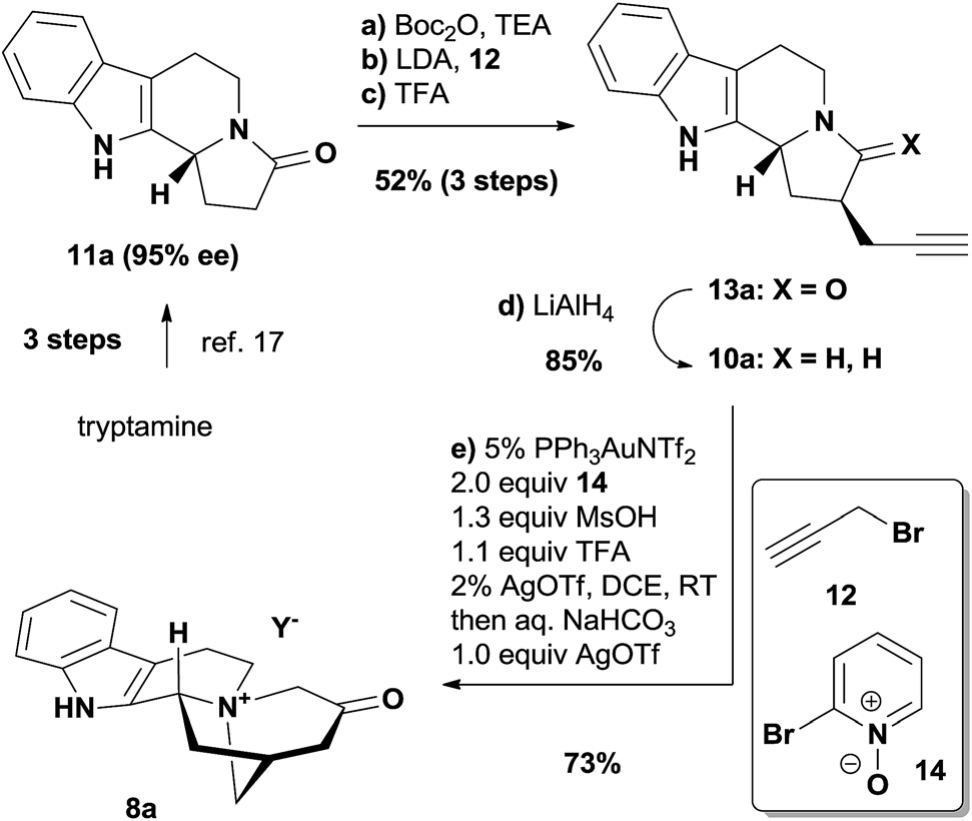
Preparation of the quaternary ammonium compound **8a**. Reagents and conditions: (a) Boc_2_O (3.0 equiv.), TEA (1.1 equiv.), DMAP (0.2 equiv.), DCM, RT, 14 h, 89%; (b) LDA (1.2 equiv.), propargyl bromide **12** (2.5 equiv.), –78 °C to RT, 2 h, THF; (c) TFA (5.0 equiv.), DCM, RT, 16 h; 58% over two steps; (d) LiAlH_4_ (3.0 equiv.), THF, 80 °C, 1 h, 85%; (e) PPh_3_AuNTf_2_ (5 mol%), **14** (2.0 equiv.), MsOH (1.3 equiv.), TFA (1.1 equiv.), AgOTf (2 mol%), DCE, RT, 6 h; then NaHCO_3_ (sat.), AgOTf (1.0 equiv.), RT, 73%.

We commenced our studies with the chiral amide **11a**, which was prepared from tryptamine in 3 steps *via* the organocatalytic Pictet–Spengler reaction reported by Jacobsen's group (Scheme 1).^17^ The introduction of the propargyl group was achieved with the protection of the nitrogen atom, and the following deprotection afforded a pair of readily separable diastereomers, where the desired stereoisomer **13a** was isolated as the major product in 52% yield over 3 steps. The subsequent reduction of **13a** by LiAlH_4_ produced the tertiary amine **10a** smoothly in 85% yield. We ultimately developed a one-pot procedure that converted **10a** to the quaternary ammonium compound **8a** in good yield (see the ESI† for the determination of the counteranion).

The extensive optimization of this gold-catalyzed reaction followed by intramolecular alkylation was carried out using racemic **10a** (Table 1 and S1†). The basicity of the tertiary amine **10a** is detrimental to the cationic gold catalysis and needs to be neutralized with the addition of another equivalent of acid.16b,^19^ Using a 10 mol% (Ph_3_P)AuNTf_2_ catalyst and 2 equiv. of MsOH additive, we examined a variety of oxidants and identified 2-bromopyridine *N*-oxide **14** as the optimal one (Table S1†). The formation of intermediate **15** was supported by LCMS analysis, and the intermediate then underwent facile cyclization upon the treatment of the reaction mixture with a saturated aqueous solution of sodium bicarbonate. The end product **8a** was obtained in 47% yield with the recovery of 39% starting material **10a** (Table 1, entry 1). Inspired by the screening of the acid additives to improve the conversion in similar transformations,^20^ we discovered that complete consumption of **10a** was achieved in 20 h at room temperature with 1.3 equiv. MsOH and 1.1 equiv. TFA as the acid additives to afford **8a** in 63% isolated yield (entry 2). The addition of 3 mol% AgOTf significantly accelerated the reaction,^21^ which gave **8a** in 69% yield on an even larger scale (200 mg scale, entry 3). We intentionally lowered the gold catalyst loading to 5 mol% for the gram scale reaction and found that the first step went to completion in 8 h at room temperature but **8a** was isolated in only 51% yield after workup (entry 4). The conditions for the gram scale reaction were further improved by discovering that the addition of 1 equiv. AgOTf effectively promoted the cyclization step, which eventually afforded **8a** in 74% isolated yield (entry 5).

**Table 1:** Gold-catalyzed synthesis of **8a**: selected optimization^*a*^

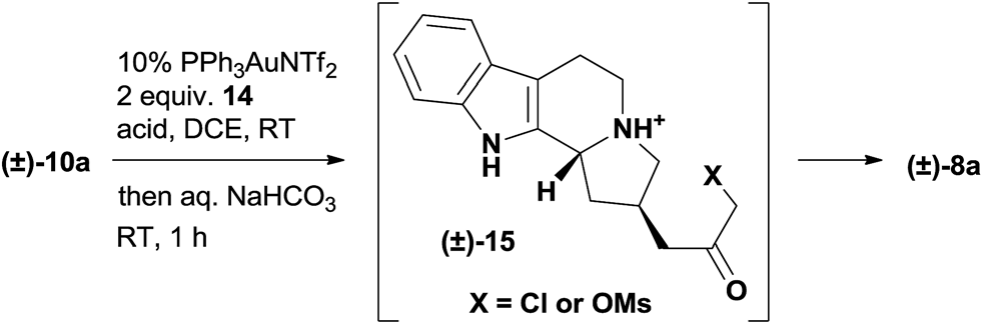			
Entry	Acid	Time	Yield^*b*^
1	2.1 equiv. MsOH	24 h	47%^*c*^
2	1.3 equiv. MsOH, 1.1 equiv. TFA	20 h	63%
3	1.3 equiv. MsOH, 1.1 equiv. TFA	6 h	69%^*d*^
4	1.3 equiv. MsOH, 1.1 equiv. TFA	8 h	51%^*e*^
5	1.3 equiv. MsOH, 1.1 equiv. TFA	8 h	74%^*f*^

^*a*^[**10a**] = 0.1 M (0.12 mmol).

^*b*^Isolated yield after flash chromatography.

^*c*^39% starting material **10a** was recovered.

^*d*^200 mg scale reaction, 3% AgOTf was added as an additive.

^*e*^1 g scale reaction, 5% PPh_3_AuNTf_2_, 2% AgOTf as an additive.

^*f*^3 g scale reaction, 5% PPh_3_AuNTf_2_, 2% AgOTf as an additive, 1 equiv. AgOTf was added with NaHCO_3_ (s, aq.) to facilitate the cyclization.

With abundant **8a** in hand, we proceeded to test the Stevens rearrangement. Initial efforts in base, solvent and temperature screening proved unfruitful, leading to either starting material recovery or decomposition (Table S2†). Inspired by the development of organocatalytic sigmatropic reactions,^22^ we shifted our focus to exploiting a novel Stevens rearrangement through the intermediacy of an enamine.^23^ By examining a variety of amines (Table S3†), we identified that 5 equiv. of piperidine **16** could promote the desired transformation in methanol even if the isolated yield of **6a** was only 11% after heating at 170 °C for 8 h in a sealed tube (Table 2, entry 1).^24^ We therefore turned to microwave technology and found that it was more effective than conventional thermal conditions (entry 2).^25^ Through a series of optimization procedures including the amount of **16** (entry 3), solvent (entry 4), concentration and heating sequence (entry 5), the *iboga* alkaloid framework **6a** was eventually obtained from **8a** in 50–60% isolated yield (over 90% yield based on starting material recovery). Furthermore, when *N*-methylmorpholine was employed in place of piperidine **16** under the optimized reaction conditions, we did not observe the formation of **6a** and the starting material **8a** was recovered in 88% yield, thus suggesting that the formal Stevens rearrangement was not base mediated. To the best of our knowledge, this transformation represents the first example of Stevens rearrangement through secondary amine catalysis.

**Table 2:** Rearrangement of **8a** to **6a**: selected optimization

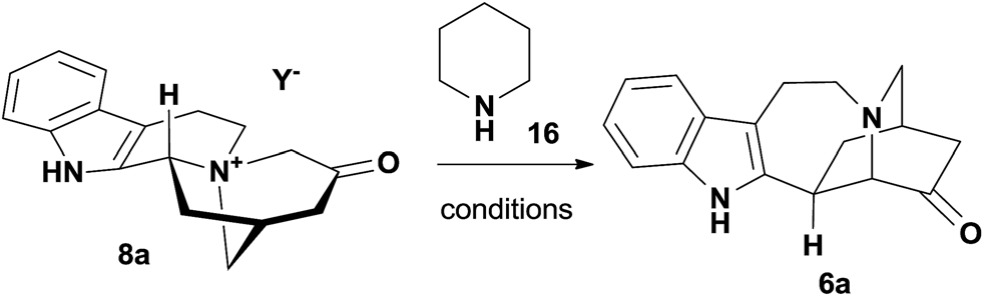			
Entry	Conditions^*a*^	Conversion^*b*^	Yield^*c*^
1	5 equiv. **16**, MeOH, 170 °C, 8 h^*d*^	46%	13%
2	5 equiv. **16**, MeOH, 120 °C (mW), 12 h	N.D.	21%
3	0.4 equiv. **16**, MeOH, 120 °C (mW), 8 h	N.D.	32%^*e*^
4	0.4 equiv. **16**, HFIP, 150 °C (mW), 3 h	45%	47%^*e*^ ^,^^*f*^
5	0.4 equiv. **16**, HFIP, 150 °C (mW), 3 h	58%	56%^*e*^ ^,^^*f*^ ^,^^*g*^

^*a*^[**8a**] = 0.1 M (0.075 mmol).

^*b*^Conversion was calculated based on the recovery of **8a**.

^*c*^Isolated yield after column chromatography.

^*d*^The reaction was carried out in a sealed tube.

^*e*^The reaction vial was pretreated by *N*,*O*-bis(trimethylsilyl)acetamide.

^*f*^[**8a**] = 0.5 M (1 mmol).

^*g*^The heating sequence was composed of 12 cycles with each cycle including irradiation at 150 °C for 15 min and at 50 °C for 15 min.

**Scheme 2:**
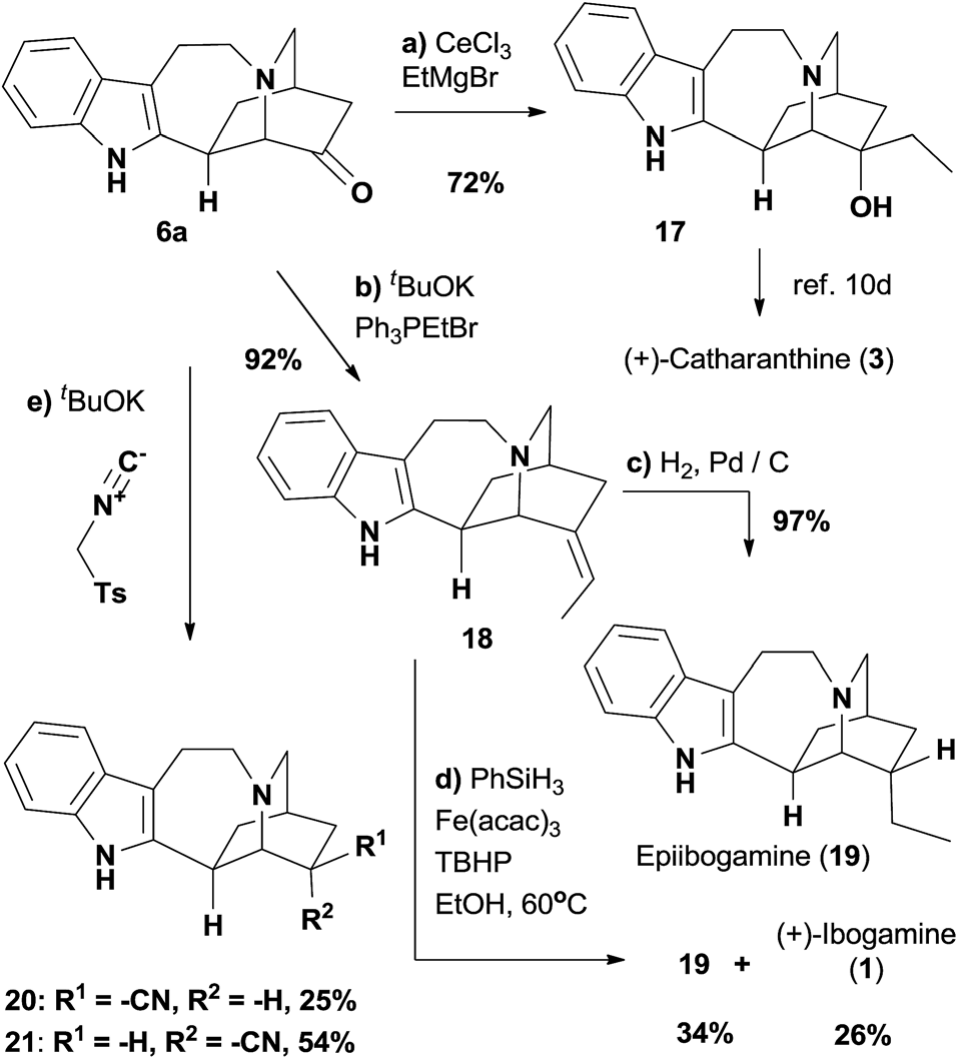
Formal synthesis of catharanthine (**3**) and the total synthesis of ibogamine (**1**). Reagents and conditions: (a) CeCl_3_ (2.5 equiv.), EtMgBr (2.0 equiv.), THF, 0.5 h, 72%; (b) ^*t*^BuOK (3.0 equiv.), Ph_3_PEtBr (3.0 equiv.), THF, 2 h, 92%; (c) H_2_, Pd/C (2.0 equiv.), MeOH, 2 h, 97%; (d) PhSiH_3_ (2.5 equiv.), Fe(acac)_3_ (0.8 equiv.), TBHP (1.5 equiv.), EtOH, 60 °C, 6 h; **19**, 34%; **1**, 26%; (e) ^*t*^BuOK (2.5 equiv.), TosMIC (1.3 equiv.), EtOH (1.7 equiv.), DME, 12 h; **20**, 25%; **21**, 54%.

The stage was set for the late-stage functional group manipulations of **6a** (Scheme 2). First, the addition of an organocerium reagent, prepared from ethylmagnesium bromide, to the ketone afforded **17** as a single diastereomer, effectively completing the formal synthesis of (+)-catharanthine (**3**).10d,12c The Wittig reaction was then employed to convert **6a** to olefin **18**, where the *Z* configuration of the trisubstituted olefin was assigned by a NOESY experiment (see ESI† for details). Hydrogenation of alkene **18** using activated Pd/C as the catalyst afforded epiibogamine **19** in 97% yield.^26^ The high stereoselectivity could be attributed to the preferential addition of H_2_ to the less hindered side of the molecule. Therefore we turned to the radical-based hydrogenation of electron-neutral alkenes initiated by hydrogen atom transfer.^27^ While manganese and cobalt-based catalyst precursors also produced **19** as the predominant product (Table S4†), we were delighted to find that Fe(acac)_3_, the precatalyst reported by Baran and co-workers for reductive alkene coupling,^28^ afforded separable **19** and (+)-ibogamine **1** in 34% and 26% yields, respectively. The key intermediate **6a** could be expediently decorated to other interesting derivatives with an *iboga* alkaloid skeleton. For instance, the reductive cyanation of ketone **6a** with tosylmethylisocyanide produced a pair of separable diastereomers **20** and **21** in 25% and 54% yields, respectively.^29^ The structures of racemic **19** and **20** were determined unambiguously by X-ray crystallography,^30^ while the analytical data of **1** and **17** corresponded well with that in the literature.12a,c

**Scheme 3:**
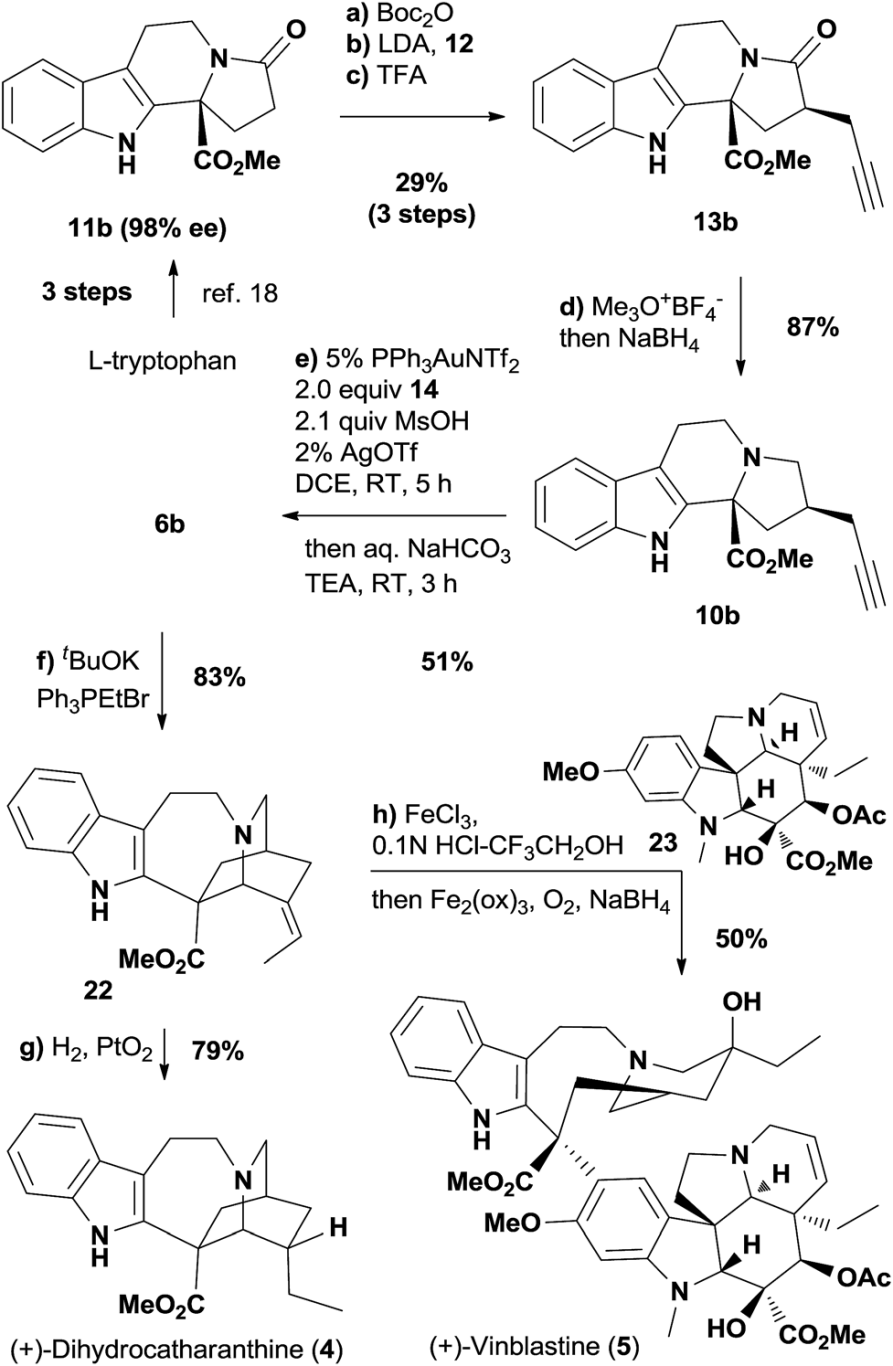
Syntheses of dihydrocatharanthine (**4**) and vinblastine (**5**). Reagents and conditions: (a) Boc_2_O (3.0 equiv.), TEA (1.1 equiv.), DMAP (0.2 equiv.), DCM, RT, 14 h, 87%; (b) LDA (1.2 equiv.), **12** (2.5 equiv.), THF, 12 h; (c) TFA (5.0 equiv.), DCM, 16 h, 33% over two steps; (d) trimethyloxonium tetrafluoroborate (2.5 equiv.), 2,6-di-*tert*-butylpyridine (3.5 equiv.), DCM, 12 h; then NaBH_4_ (0.5 equiv.), MeOH, 0.5 h, 87%; (e) PPh_3_AuNTf_2_ (5 mol%), **14** (2.0 equiv.), MsOH (2.1 equiv.), AgOTf (2 mol%), DCE, RT, 5 h; then NaHCO_3_ (sat.), TEA (3.0 equiv.), RT, 3 h, 51%; (f) ^*t*^BuOK (3.0 equiv.), Ph_3_PEtBr (3.0 equiv.), THF, 2 h, 83%; (g) H_2_, PtO_2_ (0.3 equiv.), MeOH, 15 h, 79%; (h) vindoline **23** (1.2 equiv.), HCl-CF_3_CH_2_OH, FeCl_3_ (5.0 equiv.), 2 h; Fe_2_(ox)_3_ (30 equiv.), O_2_; then NaBH_4_ (20 equiv.), 0 °C, 0.5 h, 50%.

Encouraged by the completion of (+)-ibogamine (**1**), we moved towards the synthetic study of the *iboga* alkaloids with the methoxycarbonyl group at C16 (Scheme 3). The amide **13b** was prepared from the known compound **11b** with excellent enantiopurity^18^ following the same procedures depicted in Scheme 1, while the undesired diastereomeric amide could also be converted to **13b** readily (see ESI† for details). The selective reduction of the amide carbonyl group in **13b** subsequently afforded the tertiary amine **10b**.^31^ We fortunately isolated a trace amount of the rearranged product **6b** after the work-up of the gold-catalyzed oxidation reaction of **10b**, indicating that the [1,2]-shift was quite facile in the presence of the C16 methoxycarbonyl group. Therefore, the gold-catalyzed oxidation was followed by the addition of a saturated aqueous solution of sodium bicarbonate and excess triethylamine to promote the cyclization and rearrangement. Gratifyingly, this one-pot procedure afforded ketone **6b** in 51% yield from **10b** under mild reaction conditions. The Wittig reaction of **6b** gave rise to **22**—a catharanthine isomer with an exocyclic *versus* endocyclic double bond. Hydrogenation of **22** afforded dihydrocatharanthine (**4**) in 79% yield. Interestingly, **22** differs from a known compound derived from catharanthine in the olefin geometry.^32^ Eventually, employing the conditions reported by Boger and coworkers,32*a* we successfully made vinblastine (**5**) in 50% yield by coupling **22** with commercially available vindoline **23**.

**Fig. 3:**
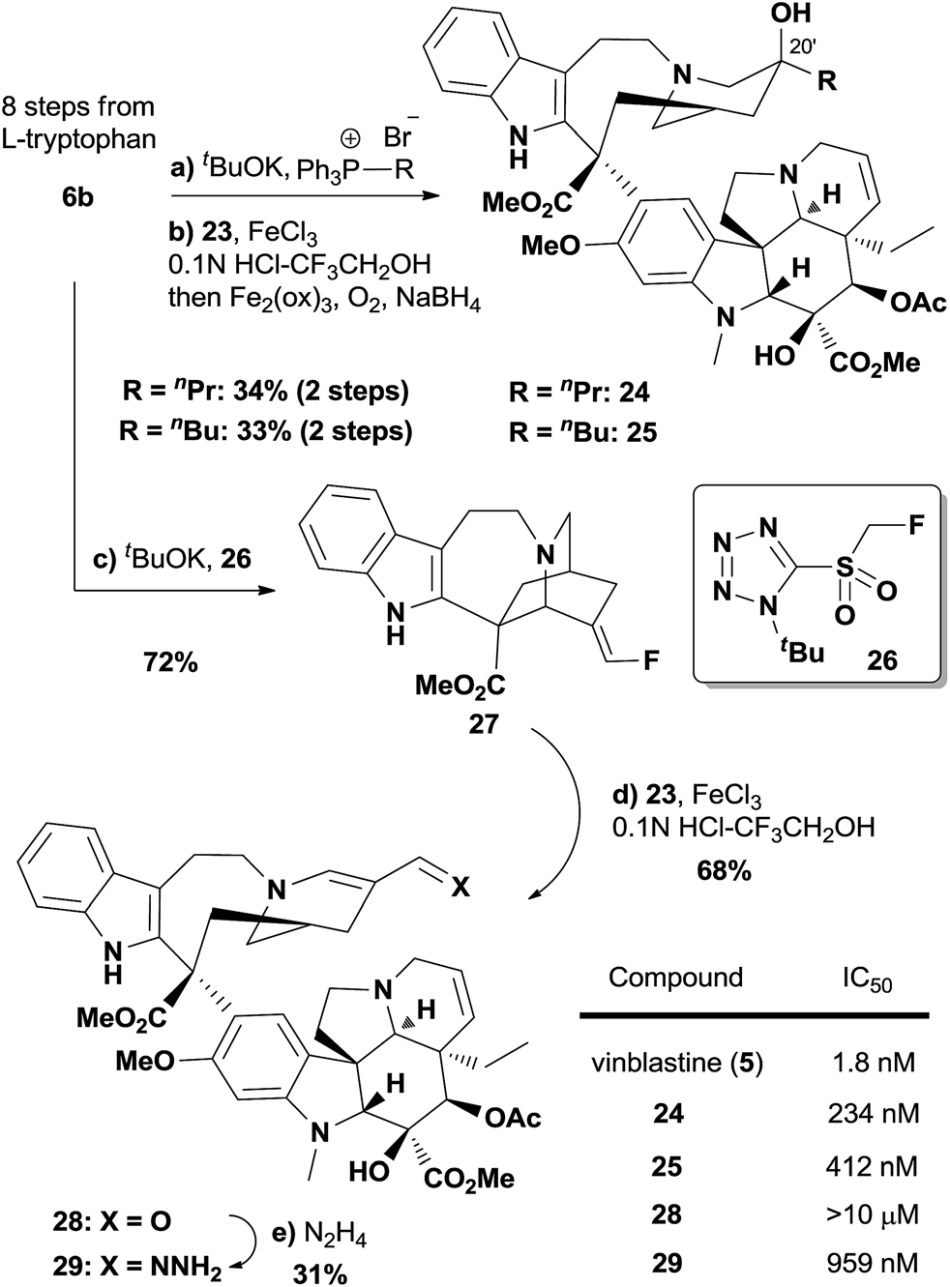
Synthesis of vinblastine analogs and their cell growth inhibitory activity.

It is noteworthy that the chiral compound **6b**, which was prepared from l-tryptophan in 8 steps, would be a valuable synthetic intermediate towards vinblastine analogs. To illustrate this point, compounds **24** and **25**, vinblastine analogs differing only in the C20′ substituent, were readily prepared by employing the Wittig reaction of **6b** followed by biomimetic coupling (Fig. 3). We also prepared fluoroalkene **27** using reagent **26**,^33^ where the *E* configuration of the olefin was assigned by a NOESY experiment (see ESI† for details). Interestingly, the coupling of **27** with vindoline (**23**) afforded aldehyde **28** in 68% yield (see Fig. S1† for a proposed mechanism). The cytotoxicities of **24** and **25** were measured in the HCT116 cell line using vinblastine (**5**) as a positive control. Our data indicated that **24** was over 100-fold less active than vinblastine, and that **25** was even less active than **24**. Based on a 40-step total synthesis, Fukuyama's group has reported inactive vinblastine analogs with C20′ acetylene functionalities that differ significantly in size and shape with the ethyl group of the natural product.^34^ Herein we showed that even a subtle change—with the C20′ alkyl substituent length extended for one (**24**) or two more carbons (**25**)—was enough to dramatically decrease the potency. This could be rationalized by the X-ray crystallographic analysis of the vinblastine–tubulin interactions, in which the C20′ ethyl substituent of vinblastine is embedded in a hydrophobic binding site.^35^ Interestingly, the aldehyde analog of vinblastine, compound **28**, almost lost the ability to inhibit the growth of HCT116 cells. However, compound **29**, obtained by the condensation of **28** with hydrazine, showed decent cytotoxicity (IC_50_ = 959 nM). This observation implies the necessity of a hydrogen bond donor around the C20′ position,^36^ although further investigation is needed to provide more insight into the hydrazone analog.

## Conclusions

In summary, we have accomplished a unique and general route for the enantioselective synthesis of *iboga* alkaloids by developing a Stevens rearrangement through secondary amine catalysis and an oxidation/cyclization/rearrangement tandem sequence. The precise mechanism of the rearrangement remains to be investigated to identify whether a radical or an ionic intermediate is involved. Nonetheless, both reactions have the potential to be applied in the synthesis of a myriad of complex alkaloids. This study nicely exemplifies the total synthesis of complex natural products serving as not only a driving force for advancing the synthetic methodology but also as an important source for providing analogs. Furthermore, this practical approach to modify *iboga* alkaloids and vinblastine paves the way for studies into their pronounced pharmacological properties using state-of-the-art chemical biology technologies, which are underway in our group and will be reported in due course.

## Supplementary Material

Supplementary information

Crystal structure data
