# Supplementary material for: Enantioselective synthesis of Iboga alkaloids and vinblastine via rearrangements of quaternary ammoniums
Source: Chem Sci. 2016 May 16;7(8):5530–6. doi: 10.1039/c6sc00932h (PMC6021789; doi:10.1039/c6sc00932h)
Supplement: Supplementary file 1 [file SC-007-C6SC00932H-s001.pdf]

Electronic Supplementary Material (ESI) for Chemical Science.
This journal is © The Royal Society of Chemistry 2016

SUPPORTING INFORMATION

Enantioselective Synthesis of *Iboga* Alkaloids and Vinblastine via Rearrangements of Quaternary Ammoniums

Yun Zhang,† YibinXue,‡ Gang Li,‡ Haosen Yuan,‡ and TuopingLuo*,‡

‡Key Laboratory of Bioorganic Chemistry and Molecular Engineering, Ministry of Education, Beijing National Laboratory for Molecular Science, College of Chemistry and Molecular Engineering, Peking-Tsinghua Center for Life Sciences, Peking University, Beijing 100871, China.

†Laboratory of Chemical Genomics, School of Chemical Biology and Biotechnology, Peking University Shenzhen Graduate School, Shenzhen 518055, China

Table of Contents

I: Tables S1-4…………………………………………………………………………………………P3

II: Experimental Procedures and Spectroscopic Data of the Synthesized Compounds …..…………P6
Determination of the Counteranion of Quanternary Ammonium 8a (Table S5).....…………….....P9
Figure S1……………………………………………………………………………………………P21

III: Cell Growth Inhibition Assay…………………………………………………………………..P23

IV: Chiral HPLC Traces for Measuring Enantiomeric Excess…………….………………..……...….P24

V: ^{1}H and ^{13}C NMR Spectra of Compounds……………..……………………………..…..….….......P26

VI: References……………………………………...………………………………………….……..…P61

I: Tables S1-4

Table S1. Gold-Catalyzed Synthesis of 8a[a]

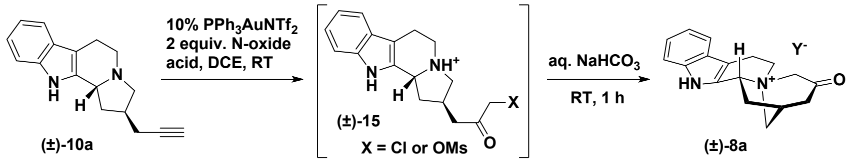

entry	N-oxide	acid	Time	converseion[b]	yield, %[c]
1	**1a**	2.1 equiv MsOH	20 h	68	36
2	**1a**	2.1 equiv TfOH	20 h	43	41
3	**1a**	2.1 equiv Tf_2NH	20 h	44	31
4	**1a**	2.1 equiv Tf_2NH	20 h	decompose[d]	
5	**1a**	2.1 equiv Cl_3CCO_2H	48 h	<5	0
6	**1a**	2.1 equiv TFA	48 h	<5	0
7	**1a**	2.1 equiv TsOH	48 h	<5	0
8	**1b**	2.1 equiv MsOH	24 h	65	43
9	**14**	2.1 equiv MsOH	24 h	63	47
10	**1d**	2.1 equiv MsOH	24 h	58	38
11	**1e**	2.1 equiv MsOH	24 h	62	0[e]
12	**1f**	2.1 equiv MsOH	20 h	100	0[f]
13	**14**	1.3 equiv MsOH<br>1.1 equiv TFA	20 h	100	63
14	**14**	1.3 equiv MsOH<br>1.1 equiv Cl_3CCO_2H	20 h	100	57
15	**14**	1.3 equiv MsOH<br>1.1 equiv TsOH	20 h	68	33
16	**14**	1.3 equiv MsOH<br>1.1 equiv TFA	6 h	100	69[g]
17	**14**	1.3 equiv MsOH<br>1.1 equiv TFA	36 h	100	52[h]
18	**14**	1.3 equiv MsOH<br>1.1 equiv TFA	36 h	100	76[i]

[a] [**10a**] = 0.1 M (0.12 mmol). [b] Conversion was calculated based on the recovery of **10a**. [c] Isolated yield after column chromatography. [d] The reaction was performed under 80 °C. [e] Compound **1g** was isolated in 69% yield. [f] Compound **1g** and **1h** was isolated in 45% and 29% yield, respectively.[g] 200 mg **10a** was used, and 3% AgOTf was added as an additive. [h] 1 g scale reaction, 5% PPh_3AuNTf_2, 2% AgOTf as additive. [i] 2 g scale reaction, 5% PPh_3AuNTf_2, 2% AgOTf as additive, 1 equiv AgOTf was added with $NaHCO_3$ (*s*, aq.) to facilitate the cyclization.

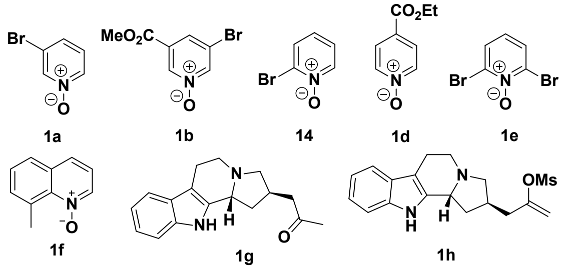

Table S2

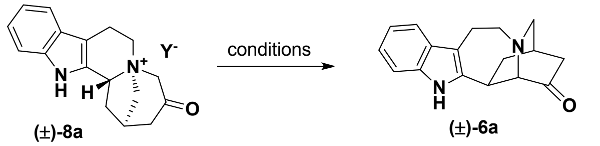

entry	conditions	conversion, %[a]	yield, %[b]
1	3 equiv CH_3SOCH_2Na, DMSO, 80^oC, 3 h	100	0
2	3 equiv NaH, DMSO, 60^oC, 5 h	100	0
3	3 equiv NaH, DMF, 60^oC, 5 h	100	0
4	5 equiv NaH, THF, 60^oC, 5 h	0	0
5	3 equiv *t*-BuOK, THF, 40^oC, 5 h	30	0
6	3 equiv *t*-BuOK, DMF, 40^oC, 5 h	65	0
7	3 equiv *t*-BuOK, DMSO, 40^oC, 5 h	68	0
8	3 equiv NaOMe, DMSO, 40^oC, 5 h	78	0
9	3 equiv NaOMe, DMF, 40^oC, 5 h	75	0
10	5 equiv DBU, ACN, rt, 5 h	38	0
11	5 equiv DBU, dioxane, rt, 5 h	42	0
12	3 equiv NaHMDS, THF, -78^oC to rt	0	0
13	3 equiv KHMDS, THF, -78^oC to rt	0	0
14	5 equiv CsF, DMF, 40^oC, overnight	0	0

[a] Conversion was calculated based on the recovery of **8a**. [b] Isolated yield after column chromatography.

Table S3

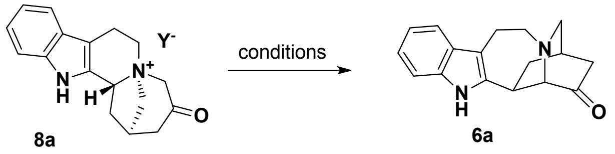

entry	conditions	converseion, %[a]	yield, %[b]
1	5 equiv **3a**, MeOH, 170^oC (sealed tube), 8 h	47	6
2	5 equiv **16**, MeOH, 170^oC (sealed tube), 8 h	46	13
3	5 equiv **3c**, MeOH, 170^oC (sealed tube), 8 h	51	0
4	5 equiv **3d**, MeOH, 170^oC (sealed tube), 8 h	90	0
5	5 equiv **3e**, MeOH, 170^oC (sealed tube), 8 h	79	0
6	5 equiv **3f**, MeOH, 170^oC (sealed tube), 8 h	37	0
7	5 equiv **3g**, MeOH, 170^oC (sealed tube), 8 h	58	0
8	5 equiv **3h**, MeOH, 170^oC (sealed tube), 8 h	0	0

[a] Conversion was calculated based on the recovery of **8a**. [b] Isolated yield after column chromatography.

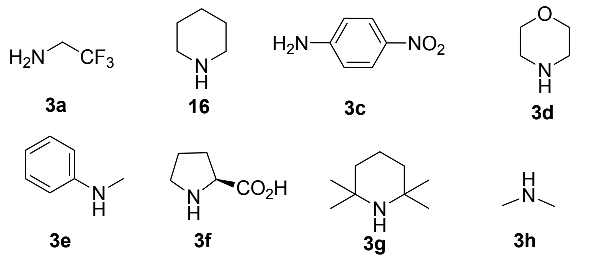

Table S4

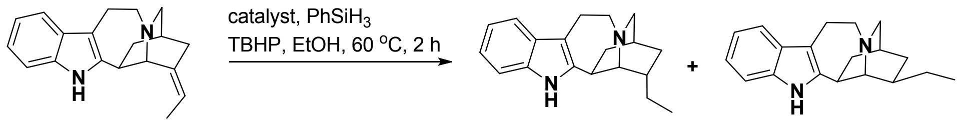

18 **19** **1**

Entry	catalyst	$PhSiH_3$ (equiv.)	TBHP (equiv.)	yield, %[a]	Ratio **19:1**
1	$Fe(acac)_3$ (0.2 equiv)	2.5	1.5	20	7:1
2[b]	$Mn(dpm)_3$ (0.2 equiv)	2.5	1.5	55	only **19** was isolated
3	$Co(dpm)_2$ (0.2 equiv)	2.5	1.5	45	only **19** was isolated
4	$Fe(acac)_3$ (0.8 equiv)	2.5	1.5	60	1.5:1
5	$Fe(acac)_3$ (1.2 equiv)	2.5	1.5	14	4:1
6	$Fe(acac)_3$ (0.8 equiv)	2.5	2	35	2.5:1
7	$Fe(acac)_3$ (0.8 equiv)	2.5	0	trace	
8	$Fe(acac)_3$ (0.8 equiv)	0	1.5	0	

[a] Isolated yield after column chromatography. [b] Reaction condition: room temperature, 3 h.

II：Experimental Procedures and Spectroscopic Data of the Synthesized Compounds

General Information.

Unless otherwise mentioned, all reactions were carried out under a nitrogen atmosphere under anhydrous conditions and all reagents were purchased from commercial suppliers without further purification. Yields refer to chromatographically and spectroscopically (^{1}H NMR) homogeneous materials.

Reactions were monitored by Thin Layer Chromatography on plates (GF254) supplied by Yantai Chemicals (China) visualized by UV or stained with ethanolic solution of phosphomolybdic acid. If not specially mentioned, flash column chromatography was performed using silica gel (200-300 mesh) supplied by Tsingtao Haiyang Chemicals (China). NMR spectra were recorded on Bruker AV400, Bruker AV500 instruments and calibrated by using residual undeuterated chloroform (δH = 7.26 ppm) and $CDCl_3$ (δC = 77.0 ppm), or undeuterated dimethyl sulfoxide (δH = 2.50 ppm) and dimethyl sulfoxide-d6 (δC = 39.5 ppm) as internal references. The 19F-NMR spectra were referenced with respect to $CFCl_3$ using externally to a neat $CFCl_3$ reference sample at 24 °C.The following abbreviations were used to explain the multiplicities: s = singlet, d = doublet, t = triplet, q = quartet, b = broad, td =triple doublet, dt = double triplet, dq = double quartet, m = multiplet. Infrared (IR) spectra were recorded on a Thermo Nicolet Avatar 330 FT-IR spectrometer or a Thermo Nicolet iS5 spectrometer. High-resolution mass spectra (HRMS) were recorded on a Bruker Apex IVFTMS mass spectrometer using ESI (electrospray ionization) as the ionization method. Mass spectrometric data were obtained using Waters 2545 Binary Gradient Module using ESI (electrospray ionization) as the ionization method.

Synthesis of compound **S1**:

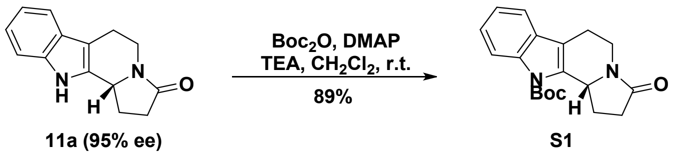

To a solution of compound **11a** (4.9 g, 21.7 mmol) in CH_2Cl_2 (108 mL) was added di-tert-butyl dicarbonate (12 mL, 52 mmol) at r.t., then added trimethylamine (3.6 mL, 26.0 mmol) and DMAP (1.1 g, 8.7 mmol), respectively. The resultant mixture was stirred at the same temperature for 14 h. The reaction mixture was diluted with DCM and quenched with a saturated solution of NH_4Cl (50 mL), and then extracted with CH_2Cl_2 (3× 50 mL). The combined organic layers were washed with brine (100 mL) and dried over Na_2SO_4. The solvent was removed under vacuum, and the residue was purified by a flash column chromatography on silica gel (petroleum ether/ethyl acetate = 2:1) to give product **S1** (6.3 g, 89% yield) as a light yellow foam; R_f = 0.40 (Silica gel, ethyl acetate); [α]20 D=+ 317(c = 1.3 in $CHCl_3$); MP: 119 °C; IR (neat): ν_{max} = 2977, 2929, 1729, 1694, 1456, 1416, 1368, 1318, 1280, 1259, 1223, 1138, 1119, 1017, 862, 839, 747 cm^{-1}; ^1H NMR (400 MHz, $CDCl_3$):δ = 8.04 (d, *J* = 8.2 Hz, 1H), 7.43 (d, *J* = 7.2 Hz, 1H), 7.31 (td,*J* = 7.7, 1.0 Hz, 1H), 7.25 (td, *J* = 7.4, 1.0 Hz, 1H), 5.23 (td, *J* = 6.7, 2.2 Hz, 1H), 4.54 (ddd, *J* = 12.7, 4.9, 1.8 Hz, 1H), 3.04 – 2.94 (m, 1H), 2.90 – 2.70 (m, 3H), 2.65 – 2.53 (m, 1H), 2.43 (ddd, *J* = 16.7, 9.7, 2.0 Hz, 1H), 1.90 – 1.77 (m, 1H), 1.70 (s, 9H) ppm;^{13}C NMR (100 MHz, $CDCl_3$):δ = 173.7, 150.0, 135.7, 135.1, 128.9, 124.6, 123.0, 118.4, 115.5, 115.5, 84.4, 56.4, 36.8, 31.4, 28.2, 27.0, 21.6 ppm; HRMS (ESI): *m/z*calcd for $C_{19}H_{23}N_2O_3^+$ [M + H]$^+$: 327.1703, found 327.1704.

Synthesis of compounds **S2** and **13a**:

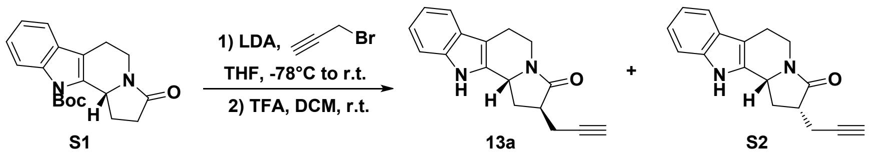

To a solution of compound **S1** (3.4 g, 10.4 mmol) in THF (52 mL) was slowly added lithium diisopropylamide (2.0M solution in THF, 6.3 mL, 12.5 mmol) dropwise at - 78 °C, and the resultant mixture was stirred at the same temperature for 0.5 h. To this solution was added 3-bromopropyne (80% w/w) (3 mL, 31.2 mmol) dropwise. The resultant mixture was warmed to room temperature slowly, and stirred at room temperature for 2 h. The reaction mixture was quenched with saturated solution of NH_4Cl (25 mL), and then extracted with ethyl acetate (3 × 50 mL). The combined organic layers were washed with brine and dried over Na_2SO_4. The solvent was removed under vacuum to obtain the crude product that was used directly in the next step.

The crude residue obtained from the last step was dissolved in CH_2Cl_2 (52 mL), and added trifluoroacetic acid (4.2 mL, 52.0 mmol) dropwise at r.t. The resultant mixture was stirred at the same temperature for 16 h. The reaction mixture was evaporated to dryness and the residue was purified by a flash column chromatography on silica gel (petroleum ether/ethyl acetate = 4:1 to 7:3) to give product **13a** (1.6 g, 58% yield over two steps) as a light yellow oil, and **S2** (0.9 g, 29% yield over two steps) as a light brown oil; compound **13a**: R_f = 0.24 (petroleum ether/ethyl acetate = 1:1); [α]20 D = + 14.5(c = 1.3 in $CHCl_3$); IR (neat): ν_{max} = 3281, 2923, 2852, 1669, 1431, 1352, 1326, 1309, 1262, 1234, 1176, 744, 641, 496 cm^{-1}; ^1H NMR (400 MHz, $CDCl_3$): δ = 8.24 (s, 1H), 7.51 (d, J = 7.7 Hz, 1H), 7.36 (d, J = 8.0 Hz, 1H), 7.23 – 7.17 (m, 1H), 7.16 – 7.11 (m, 1H), 4.95 – 4.81 (m, 1H), 4.61 – 4.46 (m, 1H), 3.14 – 2.97 (m, 1H), 2.97 – 2.79 (m, 4H), 2.75 (ddd, *J* = 17.0, 3.9, 2.8 Hz, 1H), 2.43 (ddd, *J* = 17.0, 8.2, 2.6 Hz, 1H),

1.91 (t, J = 2.6 Hz, 1H), 1.88 – 1.75 (m, 1H) ppm; ^{13}C NMR (100 MHz, $CDCl_3$): δ = 172.6, 136.3, 132.8, 126.8, 122.3, 119.9, 118.5, 111.1, 108.1, 81.1, 70.2, 52.2, 41.9, 37.8, 32.3, 21.2, 20.0 ppm; HRMS (ESI): *m/z* calcd for $C_{17}H_{17}N_2O^+$ $[M + H]^+$: 265.1335, found 265.1337; *m/z* calcd for $C_{17}H_{16}N_2NaO^+$ $[M + Na]^+$: 287.1155, found 287.1157. compound **S2**: R_f = 0.41 (petroleum ether/ethyl acetate = 1:1); [α]20 D = + 63.3(c = 0.62 in $CHCl_3$); IR (neat): ν_{max} = 3281, 2923, 2851, 1667, 1433, 1310, 1262, 1234, 1176, 1008, 800, 744, 639 cm^{-1}; ^{1}H NMR (400 MHz, $CDCl_3$): δ = 8.50 (s, 1H), 7.50 (d, J = 7.7 Hz, 1H), 7.36 (d, J = 8.0 Hz, 1H), 7.20 (t, J = 7.1 Hz, 1H), 7.13 (t, J = 7.5 Hz, 1H), 5.08 (t, J = 7.3 Hz, 1H), 4.55 (dd, J = 13.0, 5.7 Hz, 1H), 3.13 (ddd, J = 12.9, 11.5, 5.2 Hz, 1H), 2.90 (ddd, J = 11.3, 6.0, 2.2 Hz, 1H), 2.85 – 2.72 (m, 2H), 2.70 – 2.55 (m, 3H), 2.30 (ddd, J = 13.0, 9.7, 6.7 Hz, 1H), 2.02 (t, J = 2.6 Hz, 1H) ppm; ^{13}C NMR (100 MHz, $CDCl_3$): δ = 173.9, 136.3, 133.2, 126.8, 122.3, 119.9, 118.4, 111.1, 108.2, 81.3, 70.1, 53.3, 41.8, 38.2, 30.0, 21.0, 20.9 ppm; HRMS (ESI): *m/z* calcd for $C_{17}H_{17}N_2O^+$ $[M + H]^+$: 265.1335, found 265.1337; *m/z* calcd for $C_{17}H_{16}N_2NaO^+$ $[M + Na]^+$: 287.1155, found 287.1157.

Synthesis of compound **10a**:

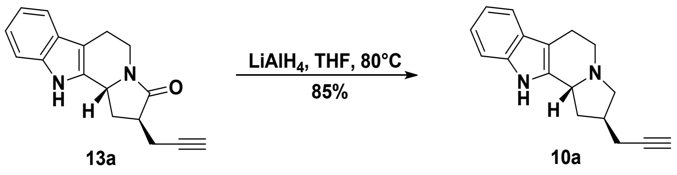

To a solution of compound **13a** (700.0 mg, 2.6 mmol) in THF (26 mL) was added $LiAlH_4$ (301.5 mg, 7.9 mmol) at 0 °C, the heterogeneous mixture was stirred at 0°C until no gas exhausted. Then the mixture was heated at 80 °C for 1 h and cooled to 0°C again. The mixture was quenched at 0°C with water (0.3 mL) slowly, NaOH (3.75 M solution in water, 0.6 mL) and water (0.9 mL) sequentially. Then the reaction mixture was filtered through a pad of celite, and washed with DCM (3×20 mL). The solvent was removed under vacuum, and the residue was purified by flash column chromatography on silica gel (DCM/MeOH = 40:1) to give product **10a** (553.2 mg, 85% yield) as a yellow oil; R_f = 0.54 (DCM/MeOH = 10:1); [α]20 D = +26.2 (c = 2.1 in $CHCl_3$); IR (neat): ν_{max} = 3399, 3285, 3053, 2918, 2846, 1672, 1449, 1309, 1280, 1264, 1233, 1142, 1009, 937, 736, 637 cm^{-1}; ^{1}H NMR (400 MHz, $CDCl_3$):δ = 7.90 (s, 1H), 7.50 (d, J = 7.5 Hz, 1H), 7.30 (d, J = 7.6 Hz, 1H), 7.19 – 7.08 (m, 2H), 4.28 (td, J = 5.0, 2.6 Hz, 1H), 3.28 (ddd, J = 12.1, 5.0, 2.3 Hz, 1H), 3.16 (dd, J = 9.7, 7.8 Hz, 1H), 3.10 – 2.89 (m, 2H), 2.73 (dd, J = 9.8, 5.3 Hz, 1H), 2.69 – 2.61 (m, 1H), 2.50 – 2.21 (m, 3H), 2.21 – 2.01 (m, 2H), 1.97 (t, J = 2.5 Hz, 1H) ppm; ^{13}C NMR (100 MHz, $CDCl_3$): δ = 136.0, 134.8, 127.4, 121.6, 119.5, 118.2, 110.8, 108.3, 83.2, 68.9, 57.4, 54.7, 46.1, 36.0, 35.6, 23.9, 17.9 ppm; HRMS (ESI): *m/z* calcd for $C_{17}H_{19}N_2^+$ $[M + H]^+$: 251.1543, found 251.1542.

Synthesis of compound **8a**:

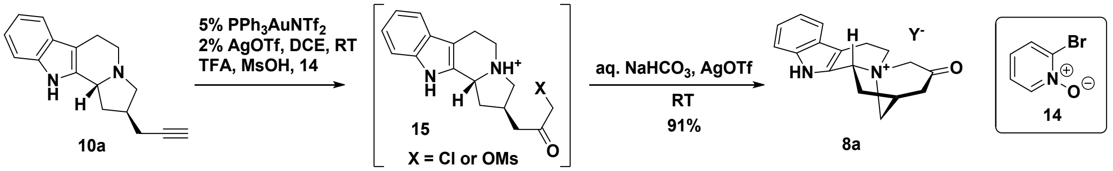

To a solution of compound **10a** (600.0 mg, 2.4 mmol) in 1,2-dichloroethane (24 mL) was added trifluoroacetic acid (220.0 μL, 2.9 mmol), methanesulfonic acid (203.0 μL, 3.1 mmol) and *N*-oxide **14** (835.2 mg, 4.8 mmol) at room temperature sequentially. The resultant mixture was stirred at the same temperature until all substances were fully

dissolved. Then PPh_3AuNTf_2 (88.7 mg, 0.12 mmol) and AgOTf (10.3 mg, 0.04 mmol) were added into the reaction mixture, respectively. The resultant mixture was stirred at room temperature for 6 h. The reaction mixture was subsequently quenched with saturated solution of $NaHCO_3$ (4 mL), after which AgOTf (739.8 mg, 2.4 mmol) was added and the reaction mixture was transferred into the separatory funnel. The two-phase mixture was vigorously shaken in the separatory funnel until the intermediates **15** were completely transformed to product **8a** (monitored by TLC and LC-MS).The reaction mixture was extracted with 10% methanol in CH_2Cl_2 (3× 30 mL). The combined organic layers were dried over Na_2SO_4. The mixture was filtered through a pad of celite and the solvent was removed under vacuum. The residue was purified by flash column chromatography on silica gel (DCM/MeOH = 50:1 to 10:1) to give product **8a** (557.1 mg, 73% yield) as a brown oil; R_f = 0.20 (DCM/MeOH = 10:1); [α]20 D = +44.7 (c = 0.92 in EtOH); IR (neat): ν_{max} = 3309, 2935, 1737, 1455, 1255, 1226, 1164, 1029, 759, 639 cm^{-1}; ^{1}H NMR (400 MHz, DMSO-d6):δ = 11.15 (s, 1H), 7.52 (d, *J* = 7.9 Hz, 1H), 7.38 (d, *J* = 8.0 Hz, 1H), 7.14 (t, *J* = 7.6 Hz, 1H), 7.06 (t, *J* = 7.4 Hz, 1H), 5.42 (dd, *J* = 8.5, 5.5 Hz, 1H), 4.57 (d, *J* = 16.8 Hz, 1H), 4.47 (d, *J* = 15.3 Hz, 1H), 4.02 (dt, *J* = 12.7, 3.4 Hz, 1H), 3.95 – 3.83 (m, 2H), 3.83 – 3.69 (m, 1H), 3.17 – 2.99 (m, 3H), 2.99 – 2.82 (m, 1H), 2.68 (d, *J* = 16.9 Hz, 1H), 2.55 (dd, *J* = 13.8, 8.7 Hz, 1H), 2.42 – 2.29 (m, 1H) ppm; ^{13}C NMR (100 MHz, DMSO-d6): δ = 200.7, 136.9, 130.2, 125.4, 125.1, 122.2, 122.1, 119.2, 119.0, 118.3, 115.8, 111.5, 102.6, 73.0, 67.5, 58.9, 56.7, 45.4, 36.6, 31.1, 16.0 ppm; ^{19}F NMR (471 MHz, DMSO) δ = -77.74 ppm; HRMS (ESI): *m/z* calcd for $C_{17}H_{19}N_2O^+$ $[M]^+$: 267.1492, found 267.1495.

Table S5.

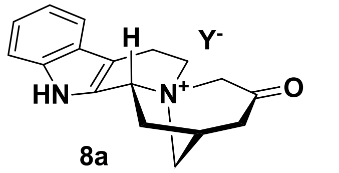

8a

Y^-	anal. calcd. C	anal. calcd. N
**$Y^- = HCO_3^-$**	65.84%	8.53%
**$Y^- = CO_3^{2-}$**	70.69%	9.42%
**$Y^- = Cl^-$**	67.43%	9.25%
**$Y^- = OTf^-$**	51.92%	6.73%
**$Y^- = MsO^-$**	59.65%	7.73%
**$Y^- = CF_3CO_2^-$**	60.00%	7.36%
Found	67.85±0.64%	8.81±0.08%

The counteranion of ammonium salt **8a** (Y^-) could be a mixture of various species and the quantitative determination of the composition turned out to be difficult given various counteranions had been introduced into the system. The elemental analysis revealed the carbon and nitrogen content of **8a** was 67.85% and 8.81%, respectively (Table S5). The following experiment was carried out to confirm the existence of CO_3^{2-}, HCO_3^- or Cl^-: the ammonium salt **8a** (30 mg) was dissolved in water (1 mL) at 50 ^{o}C, followed by the addition of AgOTf (30 mg, dissolved in 0.2 mL water); a white precipitate was observed, which disappeared upon the addition of 3 drops of MsOH. Moreover, the NMR spectra (^{13}C NMR, δ = 125.4, 122.2, 119.0 and 115.8; ^{19}F NMR, δ = -77.74 ppm) suggested the presence of

TfO$^-$. These results were most consistent with the hypothesis that majority of the counteranions would be HCO_3^-, whereas others may present in relatively small amount. The average molecular weight of **8a** could be calculated by assuming each **8a** contains only two nitrogen atoms, thus 318.0 Da.

Synthesis of compound **6a**:

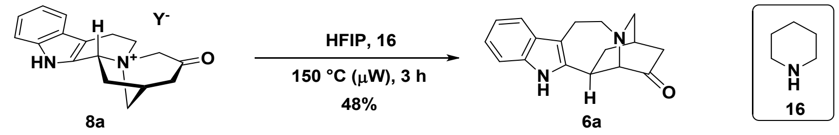

To a solution of compound **8a** (250.0 mg, 0.79 mmol) in hexafluoroisopropyl alcohol (1.9 mL) was added piperidine **16** (37 μL, 0.37 mmol) at room temperature. The resultant mixture was irradiated with microwave for a sequence of 12 cycles while each cycle involved microwaving at 150 °C for 15 min and cooling to 50 °C for 15 min. Then the solvent was removed under vacuum, and the residue was purified by flash column chromatography on silica gel (DCM/MeOH = 80:1) to give product **6a** (119.7mg, 56% yield) as a light brown foam. Part of the starting material **8a** (105.2 mg) was recovered; compound **6a**: R_f = 0.60 (DCM/MeOH = 20:1); [α]20 D = + 16 (c = 0.27 in $CHCl_3$); IR (neat): ν_{max} = 3399, 2901, 1735, 1461, 1337, 1248, 1221, 1143, 1098, 1052, 1012 cm^{-1}; ^{1}H NMR (400 MHz, $CDCl_3$):δ = 7.71 (s, 1H), 7.49 (d, *J* = 7.7 Hz, 1H), 7.27 (d, *J* = 7.8 Hz, 1H), 7.20 – 7.08 (m, 2H), 3.52 – 3.39 (m, 3H), 3.34 – 3.20 (m, 3H), 3.16 (dt, *J* = 9.5, 3.0 Hz, 1H), 2.92 – 2.76 (m, 1H), 2.68 – 2.57 (m, 1H), 2.43 – 2.31 (m, 2H), 2.11 (td, *J* = 11.6, 2.6 Hz, 1H), 1.77 (dt, *J* = 13.5, 3.6 Hz, 1H) ppm; ^{13}C NMR (100 MHz, $CDCl_3$): δ = 208.2, 138.9, 135.0, 129.5, 121.7, 119.5, 118.2, 110.4, 109.4, 66.2, 52.9, 48.1, 41.9, 37.5, 34.2, 28.4, 21.1 ppm; HRMS (ESI): *m/z* calcd for $C_{17}H_{19}N_2O^+$ $[M]^+$: 267.1492, found 267.1493.

Synthesis of compound **17**:

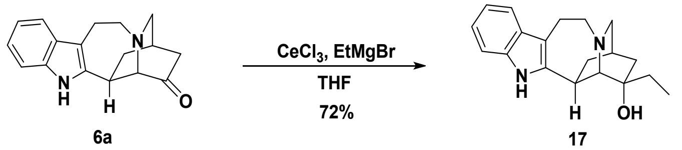

A 10 mL round-bottom flask charged with cerium (III) chloride (60 mg, 0.24 mmol) and a stirring bar was heated to 150 °C and stirred for 3h under vacuum. Then the flask was charged with nitrogen, cooled to 0 °C and added THF (0.4 mL). The resultant mixture was stirred at the same temperature for 1 h. To this mixture was added ethylmagnesium bromide (0.9M solution in THF, 0.17 mL, 0.15 mmol) at 0°C, and the resultant mixture was stirred at 0°C for 1 h. After addition of a solution of compound **6a** (20 mg, 0.07 mmol) in THF (0.4 mL) to the above prepared reaction mixture at 0°C, the resultant mixture was stirred at 0°C for 30 min, and quenched with the saturated solution of NH_4Cl (1 mL).The mixture was extracted with CH_2Cl_2 (3×5 mL).The combined organic layers were dried over Na_2SO_4. The solvent was removed under vacuum, and the residue was purified by flash chromatography on silica gel (DCM/MeOH = 100:1 to 50:1) to give product **17** (15.9 mg, 72% yield); R_f = 0.34 (DCM/MeOH = 20:1); [α]20 D = + 29 (c = 1.0 in MeOH), (Lit. [α]20 D= + 32 (c = 1.1, MeOH))[1]; IR (neat): ν_{max} = 3288, 2925, 1462, 1342, 1192, 1130, 985, 909 cm^{-1}; ^{1}H NMR (400 MHz, $CDCl_3$): δ = 7.75 (s, 1H), 7.47 (d, *J* = 7.2 Hz, 1H), 7.24 (s, 1H), 7.10 (dd, *J* = 7.1, 1.2 Hz, 2H), 3.70 – 3.57 (m, 1H), 3.37 (td, *J* = 16.1, 14.6, 4.3 Hz, 2H), 3.17 (dd, *J* = 19.4, 9.0 Hz, 1H), 3.06 (d, *J* = 9.2 Hz, 1H), 2.93 – 2.77 (m, 2H), 2.75 – 2.62 (m, 1H), 2.21 – 2.07 (m, 1H), 1.95 (s, 1H), 1.84 – 1.71 (m, 3H), 1.63 – 1.54 (m, 2H),1.31 (s, 1H), 0.94 (t, *J* = 7.4 Hz, 3H) ppm; ^{13}C NMR (100

MHz, $CDCl_3$):δ = 141.8, 134.6, 129.7, 121.0, 119.1, 117.8, 110.2, 109.4, 75.2, 61.5, 54.0, 48.2, 40.9, 34.1, 34.0, 33.8, 27.4, 20.8, 7.2 ppm; HRMS (ESI): *m/z* calcd for $C_{19}H_{25}N_2O^+$ $[M + H]^+$: 297.1961, found 297.1961.

Synthesis of compound **18**:

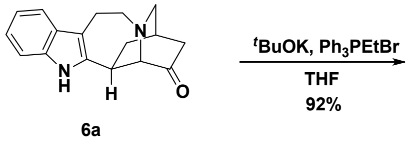

To a solution of ethyltriphenylphosphonium bromide (417 mg, 1.12 mmol) in THF (1 mL) was added potassium tert-butanolate (417 mg, 1.12 mmol) at room temperature, the resultant mixture was stirred at the same temperature for about 1h and the color of the solution turned into orange. After addition of a solution of compound **6a** (60.0 mg, 0.22 mmol) in THF (1 mL) to the above prepared reaction mixture, the resultant mixture was stirred for 2h at room temperature, and quenched with water (4 mL). The mixture was extracted with CH_2Cl_2 (3×5 mL).The combined organic layers were dried over Na_2SO_4. The solvent was removed under vacuum, and the residue was purified by flash chromatography on silica gel (DCM/MeOH =50:1 to 20:1) to give product **18** (57.6 mg, 92% yield) as a brown oil; R_f = 0.35 (DCM/MeOH = 20:1); [α]20 D = + 53(c = 0.4 in $CHCl_3$); IR (neat): ν_{max} = 3191, 2931, 2563, 1462, 1340, 1011, 827, 743 cm^{-1}; ^1H NMR (400 MHz, $CDCl_3$): δ = 7.89 (s, 1H), 7.49 (d, *J* = 7.6 Hz, 1H), 7.28 (d, *J* = 7.7 Hz, 1H), 7.19 – 7.08 (m, 2H), 5.34 (d, *J* = 6.5 Hz, 1H), 4.00 (s, 1H), 3.58 – 3.49 (m, 1H), 3.49 – 3.25 (m, 3H), 3.18 (d, *J* = 10.2 Hz, 1H), 3.16 – 3.06 (m, 1H), 2.90 (d, *J* = 15.8 Hz, 1H), 2.60 (d, *J* = 16.3 Hz, 1H), 2.38 (d, *J* = 16.1 Hz, 1H), 2.23 – 2.10 (m, 2H), 1.79 – 1.60 (m, 4H) ppm; ^{13}C NMR (100 MHz, $CDCl_3$): δ = 139.8, 135.2, 134.8, 128.8, 121.5, 119.8, 119.4, 117.8, 110.8, 108.3, 55.7, 54.7, 50.0, 37.5, 33.8, 32.8, 26.5, 19.7, 13.4 ppm; HRMS (ESI): *m/z* calcd for $C_{19}H_{23}N_2^+$ $[M]^+$: 279.1856, found 279.1854.

Synthesis of compound **19**:

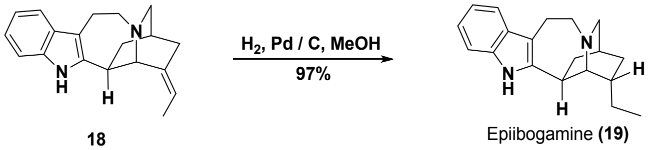

To a solution of compound **18** (20.0 mg, 0.072 mmol) in methanol (0.7 mL) was added 10% Pd/C (20 mg) at room temperature. The resultant mixture was first degassed with hydrogen, and then stirred at room temperature for 2h. The reaction was quenched by filtration of the mixture through a pad of celite, and the celite was washed with MeOH (3 × 5 mL).The solvent was removed under vacuum, and the residue was purified by flash chromatography on silica gel (DCM/MeOH =15:1) to give product **19** (19.6 mg, 97% yield) as a light yellow oil while it turned into solid upon standing; R_f = 0.35 (DCM/MeOH = 10:1); [α]20 D = + 33.6 (c = 0.3 in $CHCl_3$); MP: 177 °C; IR (neat): ν_{max} = 3053, 2911, 2848, 2247, 1455, 1362, 1342, 1250, 1202, 1145, 1105, 1010, 907, 728, 659, 514 cm^{-1}; ^1H NMR (400 MHz, $CDCl_3$):δ = 7.73 (s, 1H), 7.48 (dd, *J* = 6.1, 2.6 Hz, 1H), 7.25 (d, *J* = 7.2 Hz, 1H), 7.17 – 7.00 (m, 2H), 3.44 – 3.26 (m, 3H), 3.13 (s, 2H), 3.06 (dd, *J* = 11.5, 4.9 Hz, 1H), 2.88 (s, 1H), 2.72 – 2.61 (m, 1H), 2.08 – 1.95 (m, 3H), 1.91 – 1.86 (m, 1H), 1.70 – 1.61 (m, 1H), 1.39 (p, *J* = 7.3 Hz, 2H), 1.08 (,d, *J* = 8.8 Hz, 1H), 0.93 (t, *J* = 7.3 Hz, 3H) ppm; ^{13}C NMR (100 MHz, $CDCl_3$): δ = 141.9, 134.3, 129.6, 121.0, 119.2, 117.8, 110.2, 110.0, 57.2, 54.6, 49.5, 42.0, 35.0, 34.2, 31.7, 28.4, 26.3, 20.2, 12.1 ppm; HRMS (ESI): *m/z* calcd for $C_{19}H_{25}N_2^+$ $[M]^+$: 281.2012, found 281.2008.

Synthesis of compounds **1** and **19**:

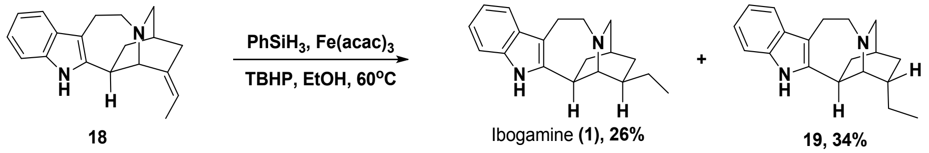

To a solution of compound **18** (100 mg, 0.36 mmol) in ethanol (3.5 mL) was added $Fe(acac)_3$ (101.3 mg, 0.29 mmol) and phenylsilane (110 µL, 0.89 mmol) at room temperature. The resultant mixture was first degassed with nitrogen, and then added tert-butyl hydroperoxide (5.0M-6.0M in decane, 98 µL, 0.54 mmol).Then the resultant mixture was degassed with nitrogen again, warmed to 60 °C and stirred at that temperature for 6 h.The reaction mixture was diluted with DCM, quenched with water (3 mL) and extracted with DCM/MeOH (10:1, 3×5 mL). The combined organic layer was dried over Na_2SO_4. The solvent was removed under vacuum, and the residue was purified by flash chromatography on silica gel (pure DCM to DCM/MeOH = 20:1) to obtain the mixture of **1** and **19**. This mixture was further separated by PTLC (DCM/MeOH/aqueous ammonia = 15:1:0.045) to give product **1** (26.1 mg, 26% yield) as a light yellow oil, and **19** (34.3 mg, 34% yield) as a light yellow oil; compound **1**: R_f = 0.45 (DCM/MeOH = 15:1); [α]20 D = + 32 (c = 0.2 in EtOH), (Lit. [α]22 D= + 42.2 (c = 0.29, EtOH))[2]; IR (neat): ν_{max} = 3221, 2924, 2856, 1965, 1633, 1462, 1162, 742 cm^{-1}; ^{1}H NMR (400 MHz, $CDCl_3$): δ = 7.63 (s, 1H), 7.49 (d, *J* = 6.9 Hz, 1H), 7.26 (dd, *J* = 7.2, 2.4 Hz, 1H), 7.18 – 7.00 (m, 2H), 3.45 – 3.32 (m, 2H), 3.22 – 3.05 (m, 2H), 3.03 – 2.90 (m, 2H), 2.87 (s, 1H), 2.74 – 2.63 (m, 1H), 2.11 – 1.99 (m, 1H), 1.90 – 1.77 (m, 2H), 1.66 (dq, *J* = 13.2, 3.4 Hz, 1H), 1.60 – 1.53 (m, 2H), 1.48 (td, *J* = 7.6, 7.1, 2.0 Hz, 1H), 1.23 (ddt, *J* = 12.6, 4.9, 2.1 Hz, 1H), 0.92 (t, *J* = 7.1 Hz, 3H) ppm; ^{13}C NMR (100 MHz, $CDCl_3$): δ = 140.8, 133.6, 128.7, 119.9, 118.1, 116.9, 109.1, 108.2, 56.6, 53.2, 48.9, 41.0, 40.4, 33.2, 31.1, 26.8, 25.5, 19.6, 10.9 ppm; HRMS (ESI): *m/z* calcd for $C_{19}H_{25}N_2^+$ $[M + H]^+$: 281.2012, found 281.2015.

Synthesis of compounds **20** and **21**:

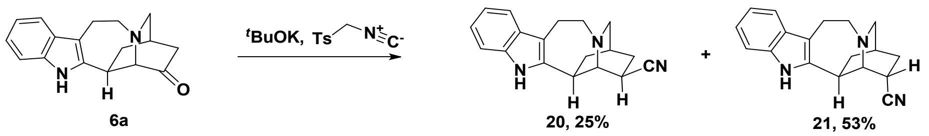

To a solution of compound **6a** (20 mg, 0.075 mmol) in 1,2-dimethoxyethane (0.35 mL) was added tosylmethylisocyanide (19 mg, 0.097 mmol), potassium tert-butanolate (21 mg, 0.187 mmol) and ethanol (7 µL, 0.127 mmol) at room temperature sequentially. The resultant mixture was first degassed with nitrogen, and then stirred at r.t. for 12 h. The solvent was removed under vacuum, and the residue was purified by flash chromatography on silica gel (petroleum ether/ethyl acetate = 4:1 to 1:1) to give product **20** (5.2 mg, 25% yield) as a light yellow solid, and product **21** (11.1 mg, 54% yield) as a light yellow solid; compound **20**: R_f = 0.45 (petroleum ether/ethyl acetate = 1:1); [α]20 D = + 3 (c = 0.7 in $CHCl_3$); MP: 215 °C; IR (neat): ν_{max} = 3332, 3052, 2924, 2849, 2240, 1674, 1618, 1563, 1488, 1461, 1362, 1342, 1333, 1264, 1254, 1164, 1018, 978, 814, 803, 736, 502 cm^{-1}; ^{1}H NMR (400 MHz, $CDCl_3$): δ = 7.63 (s, 1H), 7.48 (d, *J* = 7.1 Hz, 1H), 7.26 (d, *J* = 7.3 Hz, 1H), 7.12 (dtd, *J* = 17.1, 7.1, 1.3 Hz, 2H), 3.49 – 3.41 (m, 1H), 3.38 – 3.34 (m, 1H), 3.31 (dd, *J* = 8.0, 2.8 Hz, 1H), 3.28 – 3.22 (m, 1H), 3.22 – 3.11 (m, 2H), 3.02 (ddd, *J* = 11.5, 4.1, 2.0 Hz, 1H), 2.86 (ddd, *J* = 9.4, 7.3, 1.9 Hz, 1H), 2.79 – 2.71 (m, 1H), 2.09 – 1.96 (m, 4H), 1.80 – 1.70 (m, 1H) ppm; ^{13}C NMR (100 MHz, $CDCl_3$): δ =139.3, 134.8, 129.5, 123.2, 121.6, 119.5,

118.2, 110.2, 109.7, 56.5, 53.7, 48.8, 39.3, 33.5, 31.7, 28.8, 25.2, 20.5 ppm; HRMS(ESI): *m/z* calcd for $C_{18}H_{20}N_3^+$ $[M + H]^+$: 278.1652, found 278.1652. compound **21**: R_f = 0.15 (petroleum ether/ethyl acetate = 1:1); [α]20 D = + 44(c = 0.4 in $CHCl_3$); MP: 215 °C; IR (neat): ν_{max} = 3310, 3198, 2921, 2845, 2245, 1659, 1462, 1363, 1243, 1251, 1144, 1125, 1018, 989, 908, 824, 742, 648, 630, 503 cm^{-1};^1H NMR (400 MHz, $CDCl_3$): δ = 7.79 (s, 1H), 7.47 (d, *J* = 7.5 Hz, 1H), 7.28 (dd, *J* = 7.6, 1.2 Hz, 1H), 7.12 (dtd, *J* = 18.8, 7.1, 1.3 Hz, 2H), 3.51 (ddd, *J* = 11.7, 4.6, 1.8 Hz, 1H), 3.39 – 3.28 (m, 3H), 3.28 – 3.10 (m, 3H), 3.04 (dt, *J* = 9.8, 2.9 Hz, 1H), 2.77 – 2.65 (m, 1H), 2.23 (dtt, *J* = 14.3, 11.5, 2.8 Hz, 2H), 2.02 (p, *J* = 2.9 Hz, 1H), 1.88 (ddt, *J* = 13.5, 5.1, 2.6 Hz, 1H), 1.83 – 1.73 (m, 1H) ppm; ^{13}C NMR (100 MHz, $CDCl_3$): δ = 139.7, 134.7, 129.4, 122.4, 121.5, 119.4, 117.9, 110.4, 109.6, 55.4, 53.9, 48.5, 35.7, 34.0, 30.6, 28.8, 25.2, 20.2 ppm; HRMS (ESI): *m/z* calcd for $C_{18}H_{20}N_3^+$ $[M + H]^+$: 278.1652, found 278.1652.

Synthesis of compound **S3**:

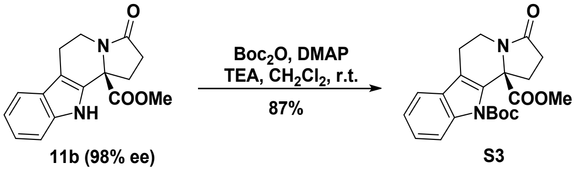

To a solution of compound **11b** (3.0 g, 10.6 mmol) in CH_2Cl_2 (52 mL) was added di-tert-butyl dicarbonate (6 mL, 25.4 mmol) at r.t. then trimethylamine (1.8 mL, 12.7 mmol) and DMAP (513 mg, 4.2 mmol) were added, respectively. The resultant mixture was stirred at r.t. for 14 h. The reaction mixture was quenched with a saturated solution of NH_4Cl (20 mL), and extracted with CH_2Cl_2 (3× 50 mL). The combined organic layers were washed with brine (100 mL) and dried over Na_2SO_4. The solvent was removed under vacuum, and the residue was purified by flash chromatography on silica gel (petroleum ether/ethyl acetate = 2:1) to give product **S3** (3.3 g, 87% yield) as a transparent oil; R_f = 0.64 (pure ethyl acetate); [α]20 D = + 284 (c = 0.6 in $CHCl_3$); IR (neat): ν_{max} = 2931, 1725, 1695, 1478, 1455, 1432, 1404, 1367, 1337, 1320, 1304, 1289, 1257, 1225, 1159, 1143, 1090, 1058, 1018, 860, 841, 803, 747 cm^{-1}; ^1H NMR (400 MHz, $CDCl_3$): δ = 7.96 (d, *J* = 8.3 Hz, 1H), 7.46 (d, *J* = 7.4 Hz, 1H), 7.36 – 7.30 (m, 1H), 7.30 – 7.23 (m, 1H), 4.54 – 4.41 (m, 1H), 3.71 (s, 3H), 3.32 (ddd, *J* = 13.1, 9.5, 1.4 Hz, 1H), 3.17 – 3.07 (m, 1H), 3.04 – 2.89 (m, 1H), 2.84 – 2.74 (m, 2H), 2.42 (ddd, *J* = 16.7, 10.0, 1.4 Hz, 1H), 2.03 (dt, *J* = 13.2, 10.4 Hz, 1H), 1.67 (s, 9H) ppm; ^{13}C NMR (100 MHz, $CDCl_3$): δ = 174.2, 170.8, 150.1, 135.3, 133.8, 128.8, 125.0, 123.0, 118.8, 116.4, 115.8, 84.8, 67.1, 52.6, 35.2, 31.0, 30.4, 28.1, 21.4 ppm; HRMS (ESI): *m/z* calcd for $C_{21}H_{25}N_2O_5^+$ $[M + H]^+$: 385.1758, found 385.1759.

Synthesis of compounds **S4** and **13b**:

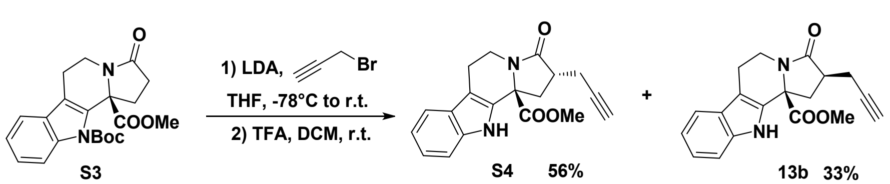

To a solution of compound **S3** (3.3 g, 8.6 mmol) in THF (43 mL) was added slowly lithium diisopropylamide (2.0 M solution in THF, 5.1 mL, 10.2 mmol) at - 78 °C, and the resultant mixture was stirred at the same temperature for 0.5 h. To this solution was added 3-bromopropyne dropwise, and the resultant mixture was warmed

up to room temperature slowly. After stirring for 12 h, the reaction mixture was quenched with a saturated solution of NH_4Cl (30 mL), and extracted with ethyl acetate (2 × 20 mL) and CH_2Cl_2 (2 ×20 mL). The combined organic layers were dried over Na_2SO_4. The solvent was removed under vacuum to afford the crude products that were used direct in the next step.

The residue obtained from last step was dissolved in CH_2Cl_2 (43 mL), and added trifluoroacetic acid (5.3 mL, 68.8 mmol) dropwise at room temperature. The resultant mixture was stirred at the same temperature for 16 h. Then the solution was evaporated *in vacuo* and the residue was purified by flash chromatography on silica gel (petroleum ether/ethyl acetate = 8:1 to 4:1) to give product **13b** (924.1 mg, 33% yield) as a light yellow oil, and **S4** (1.5 g, 56% yield) as a light yellow oil; compound **S4**: R_f = 0.45 (petroleum ether/ethyl acetate = 2:1); [α]20 D = + 73.4 (c = 1.0 in $CHCl_3$); IR (neat): ν_{max} = 3282, 3008, 2951, 1736, 1681, 1451, 1422, 1350, 1299, 1283, 1256, 1215, 1167, 1040, 746, 645 cm^{-1}; ^1H NMR (400 MHz, $CDCl_3$): δ = 8.52 (s, 1H), 7.51 (d, *J* = 7.8 Hz, 1H), 7.39 (d, *J* = 8.1 Hz, 1H), 7.24 (t, *J* = 7.7 Hz, 1H), 7.14 (t, *J* = 7.4 Hz, 1H), 4.59 (d, *J* = 12.9 Hz, 1H), 3.81 (s, 3H), 3.32 – 3.15 (m, 1H), 3.08 (dd, *J* = 12.5, 7.7 Hz, 1H), 2.98 – 2.79 (m, 3H), 2.72 (d, *J* = 17.0 Hz, 1H), 2.52 – 2.36 (m, 1H), 2.21 (t, *J* = 12.0 Hz, 1H), 1.91 (s, 1H) ppm; ^{13}C NMR (125 MHz, $CDCl_3$): δ = 172.7, 172.1, 136.7, 130.3, 126.3, 123.0, 120.0, 118.8, 111.4, 109.6, 80.6, 70.5, 63.6, 53.3, 40.5, 38.2, 36.9, 21.1, 19.6 ppm; HRMS (ESI): *m/z* calcd for $C_{19}H_{19}N_2O_3^+$ $[M + H]^+$: 323.1390, found 323.1391. compound **13b**: R_f = 0.38 (petroleum ether/ethyl acetate = 2:1); [α]20 D = + 93.3(c = 1.1 in $CHCl_3$); IR (neat): ν_{max} = 3282, 2923, 2850, 1737, 1678, 1428, 1349, 1258, 1171, 1069, 1025, 740, 653 cm^{-1}; ^1H NMR (400 MHz, $CDCl_3$): δ = 8.30 (s, 1H), 7.49 (d, *J* = 7.8 Hz, 1H), 7.38 (d, *J* = 8.1 Hz, 1H), 7.27 – 7.20 (m, 1H), 7.17 – 7.10 (m, 1H), 4.56 (dd, *J* = 13.3, 6.0 Hz, 1H), 3.80 (s, 3H), 3.37 (ddd, *J* = 13.3, 11.7, 5.1 Hz, 1H), 2.90 (ddd, *J* = 15.8, 11.6, 6.3 Hz, 1H), 2.82 – 2.64 (m, 5H), 2.44 (ddd, *J* = 17.0, 8.7, 2.6 Hz, 1H), 2.00 (t, *J* = 2.6 Hz, 1H) ppm; ^{13}C NMR (100 MHz, $CDCl_3$): δ = 174.0, 172.4, 136.6, 130.4, 126.3, 123.1, 120.1, 118.8, 111.3, 110.2, 80.9, 70.2, 64.3, 53.2, 41.1, 37.4, 35.5, 20.6, 20.6 ppm; HRMS (ESI): *m/z* calcd for $C_{19}H_{19}N_2O_3^+$ $[M + H]^+$: 323.1390, found 323.1391.

Converting compound **S4** to the desired diastereomer **13b**:

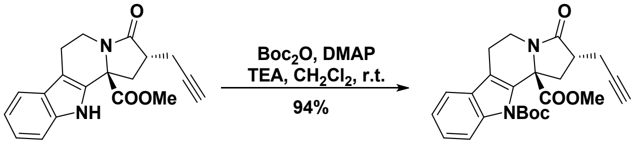

To a solution of compound **S4** (500 mg, 1.55 mmol) in CH_2Cl_2 (8 mL) was added di-tert-butyl dicarbonate (0.86 mL, 3.72 mmol) at r.t. then trimethylamine (0.30 mL, 2.17 mmol) and DMAP (57 mg, 0.46 mmol) were added, respectively. The resultant mixture was stirred at r.t. for 14 h. The reaction mixture was quenched with a saturated solution of NH_4Cl (5 mL), and extracted with CH_2Cl_2 (3 × 10 mL). The combined organic layers were washed with brine (30 mL) and dried over Na_2SO_4. The solvent was removed under vacuum, and the residue was purified by flash chromatography on silica gel (petroleum ether/ethyl acetate = 8:1) to give product **S5** (615.6 mg, 94% yield) as a transparent oil; R_f = 0.50 (petroleum ether/ethyl acetate = 4:1); [α]20 D = + 148 (c = 0.6 in $CHCl_3$); IR (neat): ν_{max} =3306, 2980, 2935, 1724, 1690, 1455, 1407, 1366, 1316, 1225, 1144, 907, 726, 646 cm^{-1}; ^1H NMR (400 MHz, $CDCl_3$): δ = 7.97 (d, *J* = 8.3 Hz, 1H), 7.46 (d, *J* = 7.5 Hz, 1H), 7.37 – 7.31 (m, 1H), 7.30 – 7.23 (m, 1H), 4.49 (dt, *J* = 13.2, 2.8 Hz, 1H), 3.70 (s, 3H), 3.53 (dd, *J* = 13.1, 8.6 Hz, 1H), 3.34 – 3.23 (m, 1H), 3.19 – 3.08 (m, 1H), 2.79 (dd, *J* = 8.9, 4.3 Hz, 2H), 2.60 (dt, *J* = 16.9, 3.5 Hz, 1H), 2.51 (ddd, *J* = 17.1, 7.1, 2.5 Hz, 1H), 2.01 (dd, *J* = 13.1, 10.6

Hz, 1H), 1.83 (s, 1H), 1.69 (s, 9H) ppm; ^{13}C NMR (100 MHz, $CDCl_3$): δ = 173.8, 170.8, 150.2, 135.3, 133.6, 128.8, 125.0, 123.0, 118.8, 116.4, 115.8, 84.9, 80.8, 70.2, 65.4, 52.6, 40.5, 36.2, 35.6, 28.2, 21.6, 20.1 ppm; HRMS (ESI): *m/z* calcd for $C_{24}H_{27}N_2O_5^+$ $[M + H]^+$: 423.1914, found 423.1918.

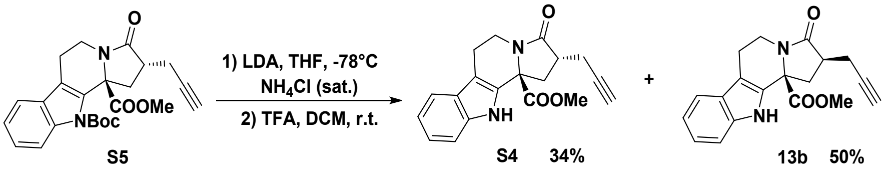

To a solution of compound **S5** (40 mg, 0.094 mmol) in THF (1 mL) was added slowly lithium diisopropylamide (2.0 M solution in THF, 0.24 mL, 0.48 mmol) at - 78 °C, and the resultant mixture was stirred at the same temperature for 45 min. To this solution was added a saturated solution of NH_4Cl (2 mL), and the resultant mixture was warmed up to room temperature. The reaction mixture was extracted with ethyl acetate (2 × 5 mL) and CH_2Cl_2 (2 × 5 mL). The combined organic layers were dried over Na_2SO_4. The solvent was removed under vacuum to afford the crude products that were used direct in the next step.

The residue obtained from last step was dissolved in CH_2Cl_2 (1.2 mL), and added trifluoroacetic acid (45 µL, 0.59 mmol) dropwise at room temperature. The resultant mixture was stirred at the same temperature for 16 h. The solution was quenched with saturated solution of $NaHCO_3$ (2 mL) and then extracted with CH_2Cl_2 (3 × 5 mL). The combined organic layers were dried over Na_2SO_4. The solvent was removed under vacuum, and the residue was purified by flash column chromatography on silica gel (petroleum ether/ethyl acetate = 8:1 to 4:1) to give product **13b** (15.2 mg, 50% yield) as a light yellow oil, and **S4** (10.3 mg, 34% yield) as a light yellow oil.

Synthesis of compound **10b**:

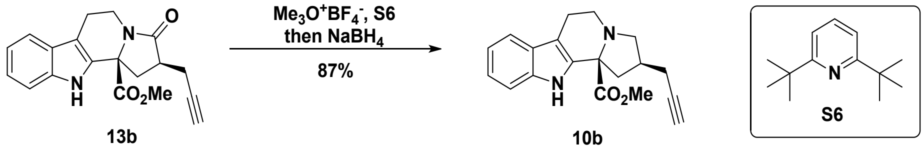

To a solution of compound **13b** (400 mg, 1.2 mmol) in CH_2Cl_2 (12 mL) was added compound **S6** (969 µL, 4.3 mmol) and trimethyloxonium tetrafluoroborate (455 mg, 3.0 mmol) at room temperature sequentially. The resultant mixture was stirred at the same temperature for 12 h. Then the reaction mixture was cooled to 0°C, and MeOH (6 mL) followed by sodium borohydride (18 mg, 0.48mmol) were added into the reaction mixture slowly. The resultant mixture was stirred at the same temperature for 30 min, quenched with a saturated solution of $NaHCO_3$ (8 mL), and extracted with CH_2Cl_2 (3× 15 mL). The combined organic layers were washed with brine (20 mL), and dried over Na_2SO_4. The solvent was removed under vacuum, and the residue was purified by flash chromatography on silica gel (petroleum ether/ethyl acetate = 4:1 to 2:1) to give product **10b** (336.6 mg, 87% yield) as a transparent oil; R_f = 0.21 (petroleum ether/ethyl acetate = 2:1); [α]20 D = + 11.7 (c = 0.5 in $CHCl_3$); IR (neat): ν_{max} = 3392, 3286, 2926, 2848, 1732, 1434, 1347, 1236, 1118, 1027, 1009, 745, 643 cm^{-1}; ^1H NMR (400 MHz, $CDCl_3$): δ = 8.20 (s, 1H), 7.50 (d, *J* = 7.9 Hz, 1H), 7.34 (d, *J* = 8.2 Hz, 1H), 7.18 (ddd, *J* = 8.2, 7.0, 1.2 Hz, 1H), 7.10 (ddd, *J* = 7.9, 7.1, 1.1 Hz, 1H), 3.77 (s, 3H), 3.39 – 3.24 (m, 2H), 3.12 (dd, *J* = 9.6, 7.4 Hz, 1H), 3.06 – 2.90 (m, 2H), 2.63 – 2.44 (m, 2H), 2.42 – 2.14 (m, 4H), 1.94 (t, *J* = 2.6 Hz, 1H) ppm; ^{13}C NMR (100 MHz, $CDCl_3$): δ = 173.9, 136.3, 131.9, 126.8, 122.4, 119.6, 118.5, 111.1, 110.5, 82.7, 69.2, 67.1, 54.1, 52.8, 43.8, 42.6, 35.6, 23.3, 15.8 ppm; HRMS (ESI): *m/z* calcd for

$C_{19}H_{21}N_2O_2^+$ [M]$^+$: 309.1598, found 309.1589.

Synthesis of compound **6b**:

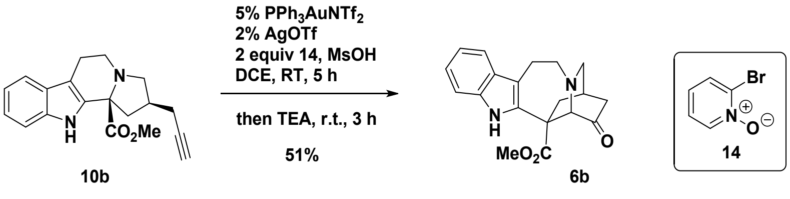

To a solution of compound **10b** (320 mg, 1.04 mmol) in 1,2-dichloroethane (10 mL) was added methanesulfonic acid (202 μL, 3.11 mmol) and compound **14** (362 mg, 2.08 mmol) at room temperature sequentially. The resultant mixture was stirred at the same temperature until all substances were fully dissolved. Then PPh_3AuNTf_2 (38 mg, 0.052 mmol) and AgOTf (3 mg, 0.010 mmol) were added into the reaction mixture, respectively. The resultant mixture was stirred at the same temperature for 5 h until the starting material was reacted completely (monitored by TLC). After the addition of the saturated solution of $NaHCO_3$ (4 mL) and triethylamine (280 μL), the reaction mixture was stirred for 3 h at room temperature, which was monitored by TLC to observe the appearance of **6b**. The reaction mixture was then extracted with CH_2Cl_2 (3 × 10 mL). The combined organic layers were dried over Na_2SO_4. The solvent was removed under vacuum, and the residue was purified by flash chromatography on silica gel (petroleum ether/ethyl acetate = 8:1) to give product **6b** (171.7 mg, 51% yield) as a transparent oil; R_f = 0.74 (petroleum ether/ethyl acetate = 1:1); [α]20 D = + 16.2(c = 0.3 in $CHCl_3$); IR (neat): ν_{max} = 2926, 1727, 1571, 1560, 1448, 1436, 1414, 1354, 1259, 1172, 1104, 1075, 1042, 954, 897, 796, 745, 699, 543, 526 cm^{-1}; ^{1}H NMR (400 MHz, $CDCl_3$): δ = 8.06 (s, 1H), 7.50 (d, J = 7.8 Hz, 1H), 7.28 (d, J = 8.0 Hz, 1H), 7.23 – 7.15 (m, 1H), 7.15 – 7.07 (m, 1H), 4.03 (s, 1H), 3.73 (s, 3H), 3.52 – 3.40 (m, 1H), 3.35 – 3.21 (m, 2H), 3.15 – 3.02 (m, 2H), 2.98 (d, J = 9.0 Hz, 1H), 2.68 – 2.54 (m, 2H), 2.41 – 2.23 (m, 2H), 2.05 (dt, J = 13.9, 3.3 Hz, 1H) ppm;^{13}C NMR (100 MHz, $CDCl_3$): δ = 204.0, 173.7, 135.6, 135.2, 128.6, 122.5, 119.6, 118.6, 110.7, 110.3, 65.4, 53.3, 53.2, 52.2, 49.6, 41.6, 38.1, 28.4, 22.4 ppm; HRMS (ESI): *m/z* calcd for $C_{19}H_{21}N_2O_3^+$ [M]$^+$: 325.1547, found 325.1548.

Synthesis of compound **22**:

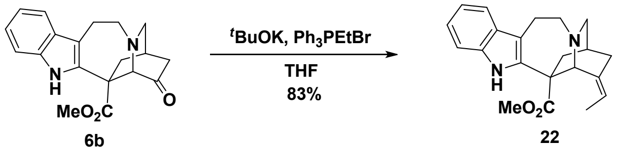

To a solution of ethyltriphenylphosphonium bromide (514 mg, 1.38 mmol) in THF (1.5 mL) was added potassium tert-butanolate (155 mg, 1.38 mmol) at room temperature. The resultant mixture was stirred at the same temperature for about 1h and the color of the solution turned into orange. After addition of a solution of compound **6b** (150 mg, 0.46 mmol) in THF (1 mL) to the above prepared reaction mixture, the resultant mixture was stirred for 2h at room temperature, and quenched with water (3 mL), and extracted with CH_2Cl_2 (3 × 5 mL).The combined organic layers were dried over Na_2SO_4. The solvent was removed under vacuum, and the residue was purified by flash chromatography on silica gel (petroleum ether/ethyl acetate = 8:1 to 1:1) to give product **22** (128.4 mg, 83% yield) as a light yellow oil; R_f = 0.14 (petroleum ether/ethyl acetate = 1:1); [α]20 D = + 24 (c = 0.8 in $CHCl_3$); IR (neat): ν_{max} = 3373, 2930, 2858, 1707, 1460, 1434, 1368, 1344, 1239, 1171, 1126, 1085, 1009, 741 cm^{-1}; ^{1}H NMR

(400 MHz, $CDCl_3$): δ = 7.73 (s, 1H), 7.51 (d, J = 7.7 Hz, 1H), 7.25 (d, J = 5.6 Hz, 1H), 7.14 (dt, J = 21.9, 6.9 Hz, 2H), 5.27 (q, J = 6.8 Hz, 1H), 4.50 (s, 1H), 3.70 (s, 3H), 3.54 (ddd, J = 15.8, 10.7, 4.8 Hz, 1H), 3.42 – 3.24 (m, 2H), 3.11 (dt, J = 9.2, 2.7 Hz, 1H), 3.06 – 2.95 (m, 2H), 2.79 (d, J = 13.6 Hz, 1H), 2.52 – 2.38 (m, 1H), 2.38 – 2.22 (m, 1H), 2.08 (s, 1H), 1.83 (dt, J = 13.6, 3.0 Hz, 1H), 1.65 (d, J = 6.9 Hz, 3H) ppm; ^{13}C NMR (100 MHz, $CDCl_3$): δ = 174.4, 137.3, 135.9, 135.3, 128.9, 122.1, 119.5, 118.4, 118.2, 110.5, 110.5, 56.4, 54.9, 53.2, 52.5, 50.5, 37.2, 33.0, 27.4, 21.6, 13.2 ppm; HRMS(ESI): *m/z* calcd for $C_{21}H_{25}N_2O_2^+$ $[M + H]^+$: 337.1910, found 337.1911.

Synthesis of compound **4**:

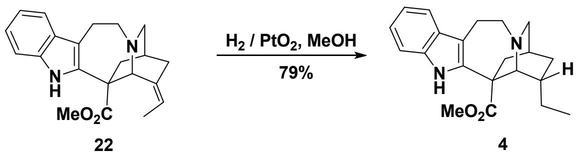

To a solution of compound **22** (20 mg, 0.059 mmol) in methanol (1 mL) was added PtO_2 (10 mg, 0.036 mmol) at room temperature. The resultant mixture was degassed with hydrogen, and then stirred at room temperature for 15h.The reaction was quenched by filtration of the mixture through a pad of celite, and the celite was washed with ethyl acetate (3 × 5 mL). The solvent was removed under vacuum, and the residue was purified by flash chromatography on silica gel (petroleum ether/ethyl acetate = 1:1) to give product **4** (15.9 mg, 79% yield) as a light yellow oil; R_f = 0.20 (petroleum ether/ethyl acetate = 1:1); [α]20 D = + 32.6 (c = 1.1 in $CHCl_3$), (Lit. [α]26 D= + 37 (c = 1.0 in $CHCl_3$))[3]; IR (neat): ν_{max} = 3373, 2930, 2858, 1707, 1460, 1433, 1248, 1171, 1085, 741 cm^{-1}; ^1H NMR (400 MHz, $CDCl_3$): δ = 7.77 (s, 1H), 7.49 (dd, J = 7.7, 1.2 Hz, 1H), 7.27 – 7.22 (m, 1H), 7.15 (ddd, J = 8.0, 7.0, 1.3 Hz, 1H), 7.10 (ddd, J = 8.1, 7.0, 1.2 Hz, 1H), 3.81 (d, J = 3.3 Hz, 1H), 3.66 (s, 3H), 3.63 – 3.51 (m, 1H), 3.20 – 2.97 (m, 4H), 2.82 (dt, J = 8.7, 1.5 Hz, 1H), 2.70 – 2.59 (m, 1H), 2.19 – 2.03 (m, 1H), 2.03 – 1.84 (m, 3H), 1.37 (ddd, J = 12.4, 7.3, 5.0 Hz, 1H), 1.23 – 1.12 (m, 1H), 1.12 – 0.97 (m, 1H), 0.92 (t, J = 7.1 Hz, 3H) ppm; ^{13}C NMR (100 MHz, $CDCl_3$): δ = 175.7, 137.2, 135.4, 128.6, 122.0, 119.3, 118.4, 110.5, 110.4, 56.3, 53.2, 52.4, 52.2, 51.4, 44.0, 37.1, 31.6, 27.4, 27.4, 21.7, 12.6 ppm; HRMS (ESI): *m/z* calcd for $C_{21}H_{27}N_2O_2^+$ $[M + H]^+$: 339.2067, found 339.2063.

Synthesis of compound **5**:

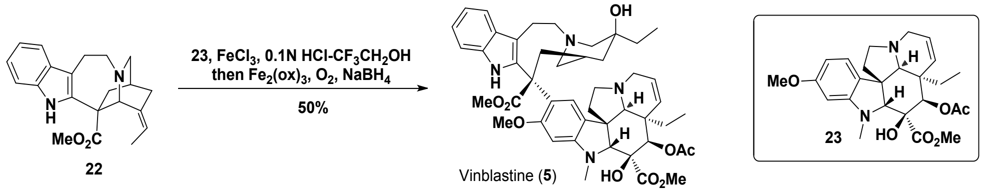

To a solution of compound **22** (20 mg, 0.059 mmol) in 2,2,2-trifluoroethanol (0.2 mL) was added compound **23** (30.5 mg, 0.071 mmol), 0.1 N HCl (1 mL), water (1 mL), and iron(III) chloride hexahydrate (48.7 mg, 0.295 mmol) at 23 °C sequentially under Ar. The resultant mixture was stirred at the same temperature for 2 h. Meanwhile, in a separate flask, a mixture of iron(III) oxalate hexahydrate (700 mg, 1.77 mmol) in H_2O (98 mL) was cooled to 0 °C and oxygen was bubbled through the mixture for 20 min. The vindoline coupling solution was transferred by pipet to this aqueous $Fe_2(ox)_3$ solution and $NaBH_4$ (36 mg, 0.952 mmol) in H_2O (2 mL) was added to the mixture at 0 °C. The resulting mixture was stirred for 30 min before being quenched by the addition of 28-30% aqueous NH_4OH. The mixture was extracted with 10% methanol in CH_2Cl_2 and the organic layers were dried over Na_2SO_4, and concentrated under reduced pressure. The residue was purified by PTLC (SiO_2, Et_3N:MeOH:EtOAc = 6:3:97) to

give product vinblastine (**5**) (24.4 mg, 50% yield) as a white solid; R_f = 0.65 (Et_3N:MeOH:EtOAc = 6:3:97); [α]20 D = + 38 (c = 0.46 in $CHCl_3$), (Lit. [α]23 D= + 40 (c = 0.46 in $CHCl_3$))[4]; IR (neat): ν_{max} = 3467, 2934, 1736, 1614, 1500, 1458, 1432, 1370, 1223, 1037, 1008, 907, 725, 645 cm^{-1}; ^1H NMR (500 MHz, $CDCl_3$): δ = 9.78 (s, 1H), 8.04 (s, 1H), 7.51 (d, *J* = 7.8 Hz, 1H), 7.18 – 7.03 (m, 3H), 6.63 (s, 1H), 6.10 (s, 1H), 5.84 (dd, *J* = 10.1, 4.3 Hz, 1H), 5.46 (s, 1H), 5.29 (d, *J* = 10.2 Hz, 1H), 3.96 (t, *J* = 14.2 Hz, 1H), 3.79 (s, 6H), 3.75 - 3.65(m, 2H), 3.61 (s, 3H), 3.44 – 3.24 (m, 4H), 3.13 (d, *J* = 14.2 Hz, 2H), 2.80 (d, *J* = 11.8 Hz, 3H), 2.70 (s, 3H), 2.66 (s, 1H), 2.46 – 2.37 (m, 2H), 2.27 (d, *J* = 13.0 Hz, 1H), 2.20 – 2.12 (m, 2H), 2.10 (s, 3H), 1.89 – 1.74 (m, 3H), 1.47 (d, *J* = 14.2 Hz, 1H), 1.43 – 1.26 (m, 4H), 0.88 (t, *J* = 7.4 Hz, 3H), 0.81 (t, *J* = 7.2 Hz, 3H) ppm; ^{13}C NMR (125 MHz, $CDCl_3$): δ = 175.0, 171.8, 170.9, 158.1, 152.7, 135.0, 131.5, 130.1, 129.5, 124.5, 123.7, 122.8, 122.2, 121.2, 118.8, 118.5, 117.0, 110.5, 94.3, 83.5, 79.7, 76.5, 69.6, 65.7, 64.4, 55.8 (2C), 55.7, 53.3, 52.4, 52.2, 50.4, 50.4, 48.2, 44.6, 42.8, 41.5, 38.4, 34.5, 34.4, 30.9, 30.2, 28.6, 21.1, 8.4, 6.9 ppm; HRMS (ESI): *m/z* calcd for $C_{46}H_{59}N_4O_9^+$ $[M + H]^+$: 811.4277, found 811.4279.

Synthesis of compound **S7**:

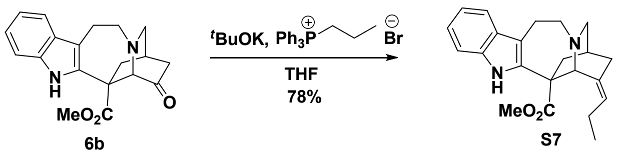

To a solution of propyltriphenylphosphonium bromide (354 mg, 0.92 mmol) in THF (1.0 mL) was added potassium tert-butanolate (104 mg, 0.92 mmol) at room temperature; the resultant mixture was stirred at the same temperature for about 1h and the color of the solution turned into orange. After addition of a solution of compound **6b** (100 mg, 0.31 mmol) in THF (0.5 mL) to the above prepared reaction mixture, the resultant mixture was stirred for 2h at room temperature, and quenched with water (3 mL), and extracted with CH_2Cl_2 (3 × 5 mL).The combined organic layers were dried over Na_2SO_4. The solvent was removed under vacuum, and the residue was purified by flash chromatography on silica gel (petroleum ether/ethyl acetate = 8:1 to 1:1) to give product **S7** (84.7 mg, 78% yield) as a light yellow oil; R_f = 0.34 (petroleum ether/ethyl acetate = 2:1); [α]20 D = + 21 (c = 0.5 in $CHCl_3$); IR (neat): ν_{max} = 3368, 2928, 2845, 1711, 1459, 1432, 1367, 1341, 1277, 1239, 1166, 1126, 1075, 1047, 1019, 997, 967, 907, 853, 809, 662, 584, 540, 504, 449, 436, 403 cm^{-1}; ^1H NMR (400 MHz, $CDCl_3$): δ = 7.65 (s, 1H), 7.49 (d, *J* = 7.7 Hz, 1H), 7.30 – 7.20 (d, 1H), 7.20 – 6.96 (m, 2H), 5.20 – 5.05 (t, 1H), 4.44 (s, 1H), 3.69 (s, 3H), 3.52 (tdd, *J* = 10.8, 5.0, 2.0 Hz, 1H), 3.30 (ttd, *J* = 15.0, 5.3, 1.4 Hz, 2H), 3.10 (dq, *J* = 6.8, 2.4 Hz, 1H), 3.06 – 2.94 (m, 2H), 2.76 (dq, *J* = 13.6, 2.2 Hz, 1H), 2.49 – 2.40 (m, 1H), 2.33 – 2.24 (m, 1H), 2.24 – 2.11 (m, 1H), 2.06 (d, *J* = 9.0 Hz, 1H), 2.03 – 1.91 (m, 1H), 1.84 (dt, *J* = 12.8, 2.5 Hz, 1H), 1.01 – 0.91 (t , 3H) ppm; ^{13}C NMR (100 MHz, $CDCl_3$): δ = 174.3, 137.3, 135.4, 134.3, 128.9, 125.8, 122.1, 119.4, 118.4, 110.5, 110.4, 77.4, 77.2, 77.0, 76.7, 56.6, 55.0, 53.0, 52.7, 50.5, 37.2, 33.1, 27.4, 21.6, 20.7, 14.4 ppm; HRMS(ESI): *m/z* calcd for $C_{22}H_{27}N_2O_2^+$ $[M + H]^+$: 351.2064, found 351.2067

Synthesis of compound **24**:

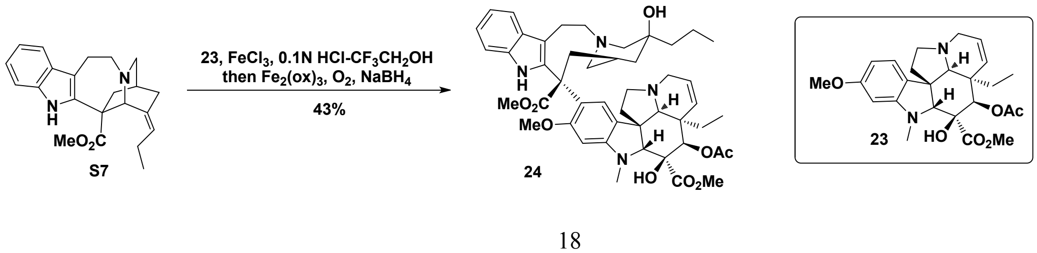

To a solution of compound **S7** (20 mg, 0.057 mmol) in 2,2,2-trifluoroethanol (0.2 mL) was added compound **23** (25.8 mg, 0.060 mmol), 0.1 N HCl (1 mL), water (1 mL), and iron(III) chloride hexahydrate (48.7 mg, 0.285 mmol) at 23 °C sequentially under Ar. The resultant mixture was stirred at the same temperature for 2 h. Meanwhile, in a separate flask, a mixture of iron(III) oxalate hexahydrate (676 mg, 1.71 mmol) in H_2O (98 mL) was cooled to 0 °C and oxygen was bubbled through the mixture for 20 min. The vindoline coupling solution was transferred by pipet to this aqueous $Fe_2(ox)_3$ solution and $NaBH_4$ (35 mg, 0.920 mmol) in H_2O (2 mL) was added to the mixture at 0 °C. The resulting mixture was stirred for 30 min before being quenched by the addition of 28-30% aqueous NH_4OH. The mixture was extracted with 10% methanol in CH_2Cl_2 and the organic layers were dried over Na_2SO_4, and concentrated under reduced pressure. The residue was purified by PTLC (SiO_2, Et_3N:MeOH:EtOAc = 6:3:97) to give product **24** (20.2 mg, 43% yield) as a white solid; R_f = 0.63 (Et_3N:MeOH:EtOAc = 6:3:97); [α]20 D = -3.8 (c = 1.0 in $CHCl_3$), IR (neat): ν_{max} = 3467, 2953, 2360, 2341, 2242, 1737, 1614, 1500, 1458, 1431, 1370, 1332, 1294, 1223, 1144, 1039, 1008, 908, 819, 668, 646, 587, 459 cm^{-1}; ^{1}H NMR (500 MHz, $CDCl_3$): δ = 9.86 (s, 1H), 8.03 (s, 1H), 7.53 (d, *J* = 7.6 Hz, 1H), 7.20 – 7.03 (m, 3H), 6.64 (s, 1H), 6.10 (s, 1H), 5.85 (dd, *J* = 10.5, 4.6 Hz, 1H), 5.47 (s, 1H), 5.30 (d, *J* = 10.2 Hz, 1H), 3.96 (t, *J* = 14.1 Hz, 1H), 3.79 (s, 5H), 3.61 (s, 3H), 3.46 – 3.22 (m, 4H), 3.17 – 3.03 (m, 2H), 2.83 (d, *J* = 16.0 Hz, 3H), 2.72 (d, *J* = 11.2 Hz, 3H), 2.67 (s, 1H), 2.49 – 2.37 (m, 2H), 2.34 – 2.22 (m, 1H), 2.09 (d, *J* = 14.5 Hz, 4H), 1.91 – 1.72 (m, 2H), 1.46 – 1.23 (m, 10H), 0.90 (t, *J* = 7.1 Hz, 3H), 0.82 (t, *J* = 7.3 Hz, 3H) ppm; ^{13}C NMR (125 MHz, $CDCl_3$): δ = 174.9, 171.8, 170.9, 158.2, 152.7, 135.0, 131.6, 130.1, 129.6, 124.5, 123.7, 122.8, 122.2, 121.3, 118.8, 118.5, 117.0, 110.5, 94.3, 83.5, 79.7, 76.5, 69.8, 65.7, 64.8, 55.8, 55.7, 53.3, 52.3, 52.2, 50.5, 50.4, 48.4, 44.6, 44.4, 42.8, 42.2, 38.4, 34.5, 30.9, 30.4, 28.9, 27.9, 21.1, 15.9, 14.8, 8.4 ppm; HRMS (ESI): *m/z* calcd for $C_{47}H_{61}N_4O_9^+$ $[M + H]^+$: 825.4425, found 825.4433.

Synthesis of compound **S8**:

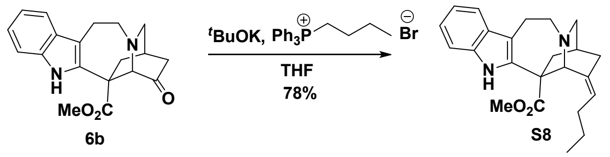

To a solution of butyltriphenylphosphonium bromide (367 mg, 0.92 mmol) in THF (1.0 mL) was added potassium tert-butanolate (104 mg, 0.92 mmol) at room temperature; the resultant mixture was stirred at the same temperature for about 1h and the color of the solution turned into orange. After addition of a solution of compound **6b** (100 mg, 0.31 mmol) in THF (0.5 mL) to the above prepared reaction mixture, the resultant mixture was stirred for 2h at room temperature, and quenched with water (3 mL), and extracted with CH_2Cl_2 (3 × 5 mL).The combined organic layers were dried over Na_2SO_4. The solvent was removed under vacuum, and the residue was purified by flash chromatography on silica gel (petroleum ether/ethyl acetate = 8:1 to 1:1) to give product **S8** (84.7 mg, 78% yield) as a light yellow oil; R_f = 0.43 (petroleum ether/ethyl acetate = 2:1); [α]20 D = +16 (c = 0.3 in $CHCl_3$); IR (neat): ν_{max} = 3368, 2953, 2926, 2858, 1710, 1459, 1432, 1367, 1278, 1239, 1167, 1126, 1076, 1046, 906, 686, 503, 450 cm^{-1}; ^{1}H NMR (400 MHz, $CDCl_3$): δ = 7.63 (s, 1H), 7.49 (dd, *J* = 7.7, 1.2 Hz, 1H), 7.29 – 7.21 (m, 1H), 7.13 (dtd, *J* = 22.1, 7.1, 1.2 Hz, 2H), 5.17 (td, *J* = 7.1, 2.2 Hz, 1H), 4.44 (s, 1H), 3.69 (s, 3H), 3.51 (ddd, *J* = 15.4, 10.2, 4.9 Hz, 1H), 3.30 (tt, *J* = 15.3, 5.2 Hz, 2H), 3.10 (dt, *J* = 9.2, 2.9 Hz, 1H), 3.07 – 2.92 (m, 2H), 2.76 (dt, *J* = 13.6, 2.5 Hz, 1H), 2.44 (dq, *J* = 16.2, 2.4 Hz, 1H), 2.28 (ddt, *J* = 16.2, 3.8, 1.9 Hz, 1H), 1.93 (dtt, *J* = 14.6, 8.3, 1.8 Hz, 1H), 1.83 (dt, *J* = 13.5, 3.2 Hz, 1H), 1.47 – 1.28 (m, 2H), 0.92 (t, *J* = 7.4 Hz, 3H) ppm; ^{13}C NMR (100 MHz, $CDCl_3$): δ = 174.4, 137.3, 135.4, 134.98, 128.9, 123.8, 122.1, 119.4, 118.4, 110.5, 110.4, 77.4, 77.2, 77.0, 76.7, 56.7, 55.0, 53.0,

52.6, 50.5, 37.2, 33.1, 29.6, 27.4, 23.1, 21.7, 14.0 ppm; HRMS(ESI): *m/z* calcd for $C_{23}H_{29}N_2O_2^+$ $[M + H]^+$: 365.2228, found 365.2224

Synthesis of compound **25**:

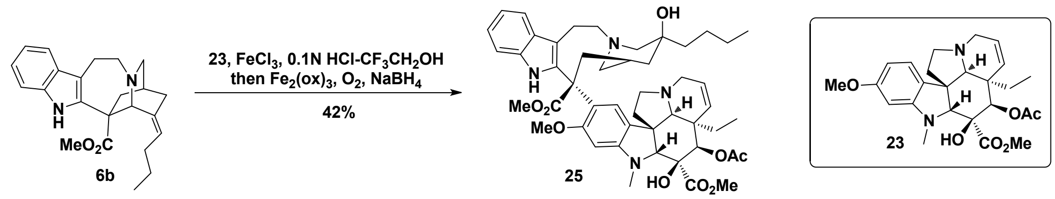

To a solution of compound **6b** (20 mg, 0.055 mmol) in 2,2,2-trifluoroethanol (0.2 mL) was added compound **23** (25.8 mg, 0.060 mmol), 0.1 N HCl (1 mL), water (1 mL), and iron(III) chloride hexahydrate (48.7 mg, 0.285 mmol) at 23 °C sequentially under Ar. The resultant mixture was stirred at the same temperature for 2 h. Meanwhile, in a separate flask, a mixture of iron(III) oxalate hexahydrate (676 mg, 1.71 mmol) in H_2O (98 mL) was cooled to 0 °C and oxygen was bubbled through the mixture for 20 min. The vindoline coupling solution was transferred by pipet to this aqueous $Fe_2(ox)_3$ solution and $NaBH_4$ (35 mg, 0.920 mmol) in H_2O (2 mL) was added to the mixture at 0 °C. The resulting mixture was stirred for 30 min before being quenched by the addition of 28-30% aqueous NH_4OH. The mixture was extracted with 10% methanol in CH_2Cl_2 and the organic layers were dried over Na_2SO_4, and concentrated under reduced pressure. The residue was purified by PTLC (SiO_2, Et_3N:MeOH:EtOAc = 6:3:97) to give product **25** (19.4 mg, 42% yield) as a white solid; R_f = 0.64 (Et_3N:MeOH:EtOAc = 6:3:97); [α]20 D = -4.9 (c = 1.2 in $CHCl_3$), IR (neat): ν_{max} = 3467, 2929, 2242, 1737, 1613, 1499, 1457, 1431, 1369, 1331, 1295, 1223, 1143, 1128, 1039, 1008, 908, 818, 729, 645, 587, 543, 483 cm^{-1}; ^1H NMR (500 MHz, $CDCl_3$): δ = 9.87 (s, 1H), 8.04 (s, 1H), 7.53 (d, *J* = 7.7 Hz, 1H), 7.24 – 7.01 (m, 3H), 6.64 (s, 1H), 6.10 (s, 1H), 5.85 (dd, *J* = 10.5, 4.4 Hz, 1H), 5.47 (s, 1H), 5.30 (d, *J* = 10.2 Hz, 1H), 3.96 (t, *J* = 14.1 Hz, 1H), 3.79 (s, 3H), 3.67 (d, *J* = 47.7 Hz, 3H), 3.46 – 3.20 (m, 3H), 3.12 (d, *J* = 13.6 Hz, 2H), 2.83 (d, *J* = 15.9 Hz, 3H), 2.72 (d, *J* = 12.0 Hz, 3H), 2.67 (s, 1H), 2.43 (dt, *J* = 15.9, 9.9 Hz, 2H), 2.28 (d, *J* = 13.4 Hz, 1H), 2.23 – 2.03 (m, 4H), 1.90 – 1.66 (m, 1H), 1.61 (s, 1H), 1.53 – 1.19 (m, 9H), 0.89 (t, *J* = 6.8 Hz, 2H), 0.82 (t, *J* = 7.3 Hz, 2H) ppm; ^{13}C NMR (125 MHz, $CDCl_3$): δ = 174.9, 171.8, 170.9, 158.2, 152.7, 135.0, 131.6, 130.1, 129.6, 124.5, 123.7, 122.8, 122.2, 121.3, 118.8, 118.5, 117.0, 110.5, 94.3, 83.5, 79.7, 76.5, 69.8, 65.7, 64.8, 55.9, 55.8, 55.7, 53.3, 52.4, 52.2, 50.5, 50.4, 48.4, 44.6, 42.8, 42.2, 41.7, 34.5, 30.9, 30.4, 29.7, 28.9, 24.8, 23.3, 21.1, 14.1, 8.4 ppm; HRMS (ESI): *m/z* calcd for $C_{48}H_{63}N_4O_9^+$ $[M + H]^+$: 839.4573, found 839.4589.

Synthesis of compound **27**:

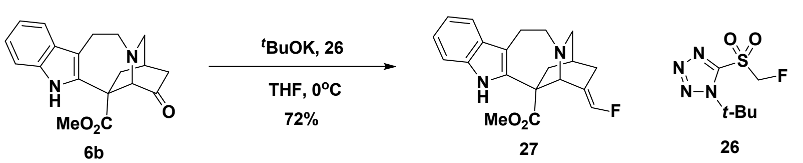

To the solution of **26** (116 mg, 0.52 mmol) and ketone **6b** (100 mg, 0.31 mmol) in THF (1.6 mL) at 0°C were added dropwise a solution ^{t}BuOK (70 mg, 0.62 mmol) in THF (0.8 mL). The mixture was stirred at 0°C for 3h until TLC indicated that the reaction was completed, then quenched with a saturated aqueous solution of NH_4Cl, and extracted with EtOAc. The combined organic layers were washed with brine, dried over Na_2SO_4, and concentrated. The residue was purified by flash chromatography on silica gel (petroleum ether/ethyl acetate = 4:1 to 1:1) to give product **27** (76 mg, 72% yield) as a light yellow oil. R_f = 0.21 (petroleum ether/ethyl acetate = 1:1); [α]20 D = + 119

(c = 0.5 in $CHCl_3$); IR (neat): ν_{max} = 3466, 2960, 1737, 1592, 1501, 1460, 1424, 1369, 1341, 1301, 1219, 1146, 1129, 1092, 1038, 958, 746, 664, 617, 588, 540, 483 cm^{-1}; ^1H NMR (500 MHz, $CDCl_3$): δ = 7.74 (s, 1H), 7.49 (d, *J* = 7.8 Hz, 1H), 7.31 – 7.22 (m, 2H), 7.14 (dddd, *J* = 30.4, 8.0, 7.0, 1.1 Hz, 2H), 6.65 (t, *J* = 2.7 Hz, 1H), 6.48 (t, *J* = 2.7 Hz, 1H), 4.06 (d, *J* = 2.5 Hz, 1H), 3.75 (s, 3H), 3.51 (ddd, *J* = 15.4, 10.2, 4.8 Hz, 1H), 3.29 (tt, *J* = 15.3, 5.1 Hz, 2H), 3.14 (dt, *J* = 9.6, 2.9 Hz, 1H), 3.00 (dd, *J* = 11.2, 4.8 Hz, 2H), 2.76 (dt, *J* = 13.8, 2.6 Hz, 1H), 2.45 (q, *J* = 2.7 Hz, 2H), 2.17 (q, *J* = 3.0 Hz, 1H), 1.92 (dt, *J* = 13.5, 2.5 Hz, 1H), 1.67 (s, 13H) ppm; ^{13}C NMR (126 MHz, $CDCl_3$): δ=174.1, 153.2, 144.4, 142.4, 136.2, 135.3, 128.7, 122.3, 119.6, 118.4, 110.6, 110.5, 77.3, 77.2, 77.2, 77.0, 76.8, 59.2, 56.5, 56.4, 55.2, 52.9, 52.9, 50.3, 37.5, 27.7, 26.5, 26.2, 26.2, 21.4 ppm; HRMS(ESI): *m/z* calcd for $C_{20}H_{22}FN_2O_2^+$ $[M + H]^+$: 341.1665, found 341.1660.

Synthesis of compound **28**:

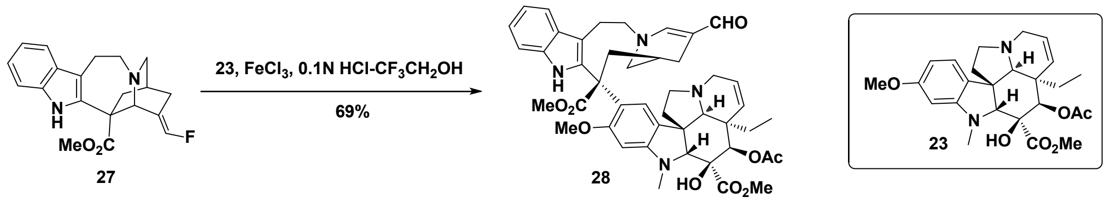

A possible mechanism for the formation compound **28**:[6]

Figure S1.

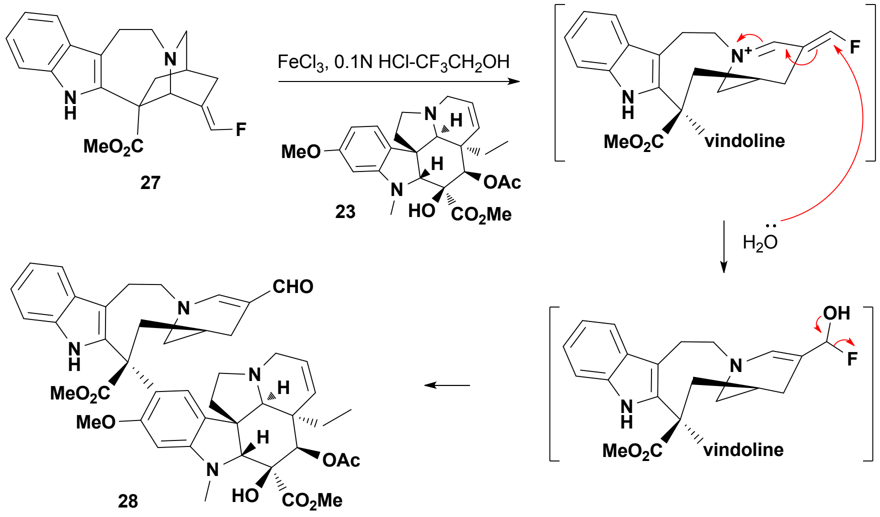

To a solution of compound **27** (20 mg, 0.055 mmol) in 2,2,2-trifluoroethanol (0.2 mL) was added compound **23** (25.8 mg, 0.060 mmol), 0.1 N HCl (1 mL), water (1 mL), and iron(III) chloride hexahydrate (48.7 mg, 0.285 mmol) at 23 °C sequentially under Ar. The resultant mixture was stirred at the same temperature for 4 h. After completion of the reaction the mixture was extracted with 10% methanol in CH_2Cl_2 and the organic layers were dried over Na_2SO_4, and concentrated under reduced pressure. The residue was purified by flash chromatography on silica gel (petroleum ether/ethyl acetate = 4:1 to 1:1) to give product **28** (31 mg, 69% yield) as white powder; R_f = 0.57 (Et_3N:MeOH:EtOAc = 6:3:97); [α]20 D = +49.7 (c = 1.0 in $CHCl_3$), IR (neat): ν_{max} = 3289, 2935, 2927, 1708, 1459, 1434, 1398, 1369, 1342, 1253, 1235, 1166, 1089, 1024, 968, 805, 736, 702, 672, 613, 538, 517, 458, 439 cm^{-1}; ^1H NMR (500 MHz, $CDCl_3$): δ = 9.70 (s, 1H), 9.04 (s, 1H), 8.06 (s, 1H), 7.51 (d, *J* = 7.7 Hz, 1H), 7.24 – 7.12 (m, 3H),

7.05 (s, 1H), 6.73 (s, 1H), 6.15 (s, 1H), 5.96 – 5.78 (m, 1H), 5.51 (s, 1H), 5.33 (d, *J* = 10.3 Hz, 1H), 3.80 (d, *J* = 14.6 Hz, 7H), 3.69 (dd, *J* = 13.3, 4.7 Hz, 1H), 3.62 (d, *J* = 13.2 Hz, 1H), 3.56 (s, 3H), 3.52 – 3.39 (m, 2H), 3.37 – 3.30 (m, 2H), 3.20 (dd, *J* = 15.4, 11.3 Hz, 1H), 3.02 (d, *J* = 12.8 Hz, 2H), 2.91 – 2.73 (m, 5H), 2.68 (s, 1H), 2.44 (td, *J* = 10.4, 6.8 Hz, 1H), 2.30 (ddd, *J* = 13.7, 9.1, 6.8 Hz, 1H), 2.19 – 2.00 (m, 6H), 1.85 (dt, *J* = 13.2, 8.1 Hz, 2H), 1.36 (dt, *J* = 13.4, 6.8 Hz, 4H), 0.86 (t, *J* = 7.4 Hz, 3H) ppm; ^{13}C NMR (126 MHz, $CDCl_3$): δ = 187.8, 173.8, 171.7, 171.0, 158.2, 154.2, 153.3, 134.8, 132.4, 130.0, 128.9, 124.5, 123.2, 123.1, 122.7, 120.6, 119.5, 117.9, 114.2, 112.5, 110.84, 94.4, 83.4, 79.6, 77.3, 77.2, 77.2, 77.0, 76.9, 76.8, 76.4, 66.3, 55.9, 55.8, 55.5, 53.3, 52.5, 52.4, 50.8, 50.6, 49.6, 45.8, 44.7, 42.8, 38.2, 33.4, 30.9, 28.4, 27.1, 26.2, 21.2, 8.6 ppm; HRMS (ESI): *m/z* calcd for $C_{45}H_{53}N_4O_9^+$ $[M + H]^+$: 793.3819, found 793.3807.

Synthesis of compound **29**:

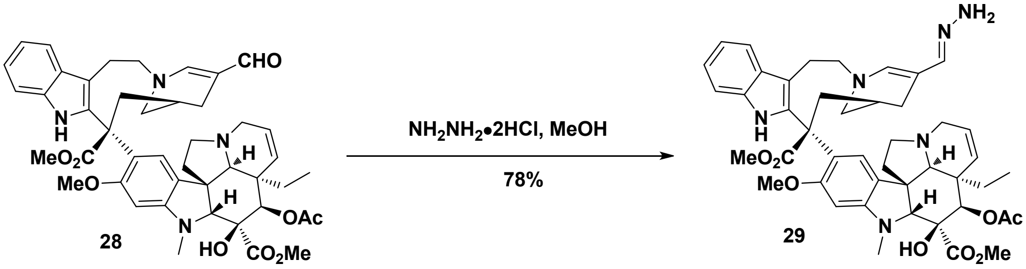

The aldehyde **28** (38mg, 0.048mmol) dissolved in MeOH (0.5mL) was added hydrazine dihydrochloride (5mg, 0.048mmol) at room temperature and stirred at 40 °C for 4 hours and the reaction was monitored by TLC. Then the mixture was filtered through syringe Millipore filter and the liquid was concentrated to provide crude hydrazone **29** (30mg, 78% yield) as yellow solid. Further purification by prep-TLC afforded pure hydrazine **29** (12 mg, 31% yield); R_f = 0.21 (MeOH:DCM = 1:10); [α]20 D = -31 (c = 0.11 in $CHCl_3$), IR (neat): ν_{max} = 3466, 2923, 2876, 2848, 2574, 2242, 1736, 1611, 1589, 1500, 1456, 1431, 1409, 1367, 1325, 1216, 1147, 1108, 1038, 1019, 908, 822, 726, 645, 617, 586, 483 cm^{-1}; ^{1}H NMR (500 MHz, $CDCl_3$): δ = 9.73 (s, 1H), 8.04 (s, 0H), 7.57 – 7.47 (m, 1H), 7.22 – 7.06 (m, 3H), 6.74 (s, 0H), 6.15 (s, 1H), 5.87 (dd, *J* = 9.8, 4.2 Hz, 1H), 5.52 (s, 1H), 5.32 (dd, *J* = 10.3, 2.2 Hz, 1H), 3.80 (dt, *J* = 9.8, 3.3 Hz, 6H), 3.57 (d, *J* = 3.1 Hz, 3H), 3.47 – 3.30 (m, 2H), 3.30 – 3.13 (m, 1H), 3.09 – 2.96 (m, 1H), 2.88 – 2.64 (m, 3H), 2.45 (td, *J* = 10.5, 6.8 Hz, 1H), 2.34 – 2.21 (m, 1H), 2.19 – 2.02 (m, 4H), 1.94 – 1.78 (m, 2H), 1.38 (tt, *J* = 12.8, 6.5 Hz, 1H), 0.95 – 0.74 (m, 3H) ppm; ^{13}C NMR (126 MHz, $CDCl_3$): δ = 174.0, 171.7, 170.1, 161.1, 158.2, 153.1, 144.3, 134.7, 132.0, 130.0, 129.0, 124.5, 123.3, 123.1, 122.5, 121.0, 119.3, 118.0, 115.1, 110.7, 106.4, 83.5, 79.6, 76.4, 66.2, 55.9, 55.6, 55.2, 53.3, 52.3, 50.7, 50.5, 48.6, 44.7, 42.8, 38.3, 33.9, 30.9, 29.7, 29.4, 28.1, 26.9, 25.2, 21.2, 8.6 ppm; HRMS (ESI): *m/z* calcd for $C_{45}H_{54}N_6O_8^+$ $[M + H]^+$: 806.4013, found 806.3998.

III：Cell Growth Inhibition Assay

The in vitro cytotoxicity was assayed in tumor cells by a tetrazolium-based colorimetric assay (Promega), which takes advantage of the metabolic conversion of MTS (3-(4,5-dimethylthiazol-2-yl)-5-(3-carboxymethoxyphenyl)-2-(4-sulfenyl)-2H-tetrazolium, inner salt) to a reduced form that absorbs light at 490 nm (ref). Compounds were tested for their cell growth inhibition of HCT116 (ATCC #CCL-247, human colorectal carcinoma) cells in culture alongside vinblastine as a control.

A population of cells (~ 1 x 10^6 cells/mL as determined with a hemocytometer) was diluted with an appropriate amount of Dulbecco-modified Eagle Medium (DMEM, Gibco) containing 10% fetal bovine serum (FBS, Gibco) to a final concentration of 30,000 cells/mL. To each well of a 96-well plate (Corning Costar), 100 µL of the cell-media solution was added. The cultures were incubated at 37 °C in an atmosphere of 5% CO_2 and 95% humidified air for 24 h. Then the cultures were removed and the test compounds were added to the plates as follows: test substances were dissolved in DMSO as the stocks and 4-fold or 2-fold serial dilutions were performed. Fresh culture medium containing 2% FBS was used to dilute the stocks to their working concentrations (for vinblastine **5**: 10.2 µM, 2.6 µM, 640 nM, 160 nM, 40 nM, 10 nM, 2.5 nM, 625 pM, 156 pM, 0 pM; for **24** and **25**: 128 µM, 64 µM, 32 µM, 16 µM, 8 µM, 4 µM, 2 µM, 1 µM, 0.5 µM, 0 µM; for **28**: 50 µM, 25 µM, 12.5 µM, 6.3 µM, 3.1 µM, 1.6 µM, 0.78 µM, 0.39 µM, 0 µM; for **29**: 20 µM, 5 µM, 1.25 µM, 0.31 µM, 78 nM, 20 nM, 5 nM, 1.25 nM, 0 nM). 100 µL of the solutions with desired concentrations above were added. Then, cultures were incubated for additional 72 h. After the given incubation time, 20 µL of the MTS assay solution (Promega) was added to each well. The plates were incubated at 37 °C for another 3 h, and the absorbency of the medium at 490 nm was measured with a spectrophotometer to obtain the number of surviving cells relative to blank control groups (no cell, 0 µM compounds, with MTS solution). Compounds were tested in triplicate (n = 2-8 times) and the results are expressed as mean cytotoxic contrations (IC_{50}s).

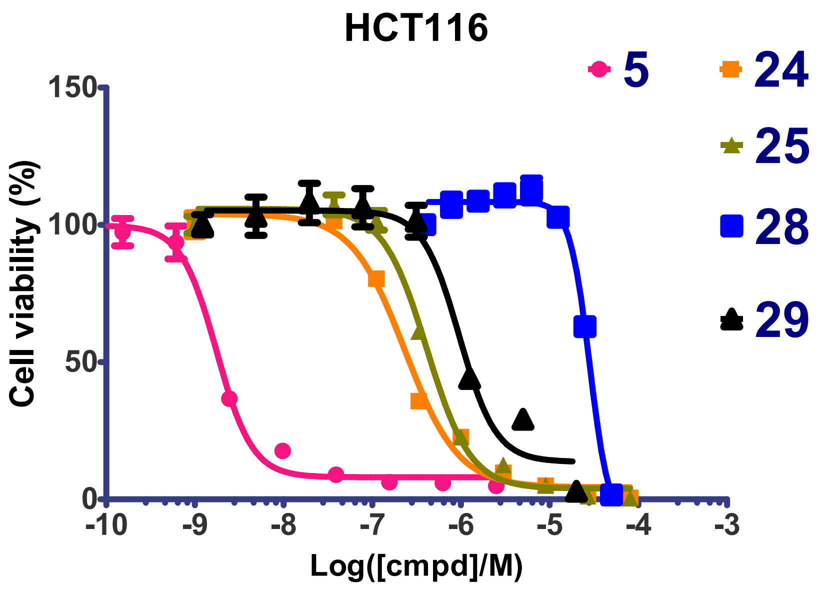

IV：Chiral HPLC Traces for Measuring Enantiomeric Excess

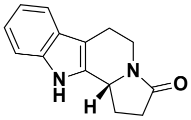

11a

A racemic sample of compound **11a** was obtained through the Pictet–Spengler reaction. The racemic and optically active **11a** were analyzed with HPLC (CHIRALPAK IA column, iPrOH : hexane = 10 : 90, 1.0 mL/min) and a 220nm UV detector to determine the retention time and enantiomeric excess. For compound **11a**, e.e. = 95%.

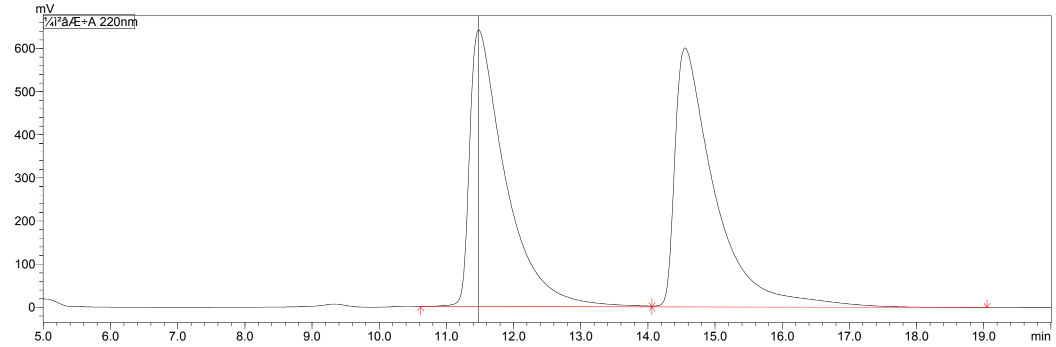

No.	Ret. Time	Area	Height	Rel. Area
1	11.482	23799869	641260	48.982
2	14.553	24789094	600408	51.018
Total:		48588963	1241668	100.000

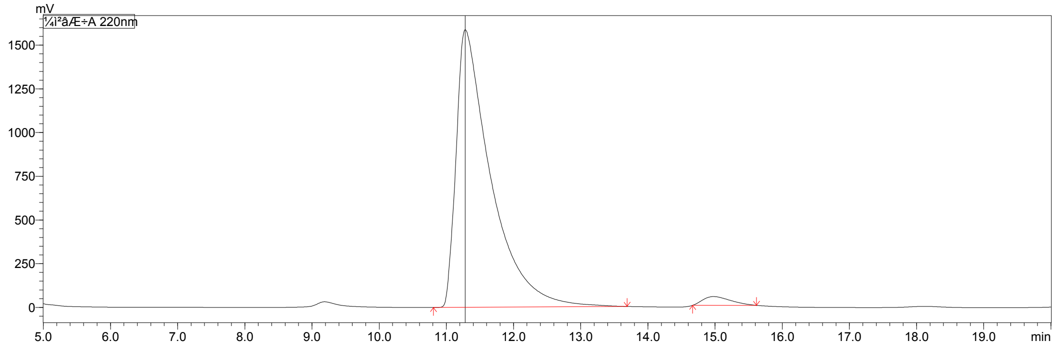

No.	Rel. Time	Area	Height	Rel. Area
1	11.281	56856676	1587685	97.535
2	14.977	1437043	50028	2.465
Total:		58293718	1637714	100.000

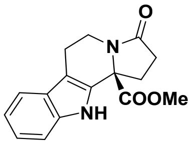

11b

A racemic sample of compound **11b** was obtained through the Pictet–Spengler reaction. The racemic and optically active **11b** were analyzed with HPLC (CHIRALPAK IB column, iPrOH : hexane = 30 : 70, 1.0 mL/min) and a 281nm UV detector to determine the retention time and enantiomeric excess. For compound **11b**, e.e. = 98%.

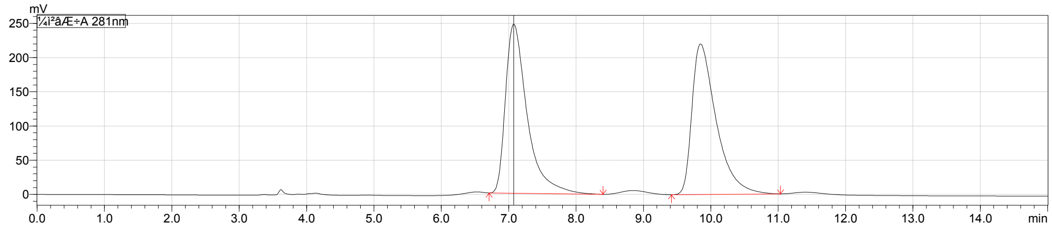

No.	Rel. Time	Area	Height	Rel. Area
1	7.075	5523336	247134	50.095
2	9.847	5502316	220102	49.905
Total:		11025652	467236	100.000

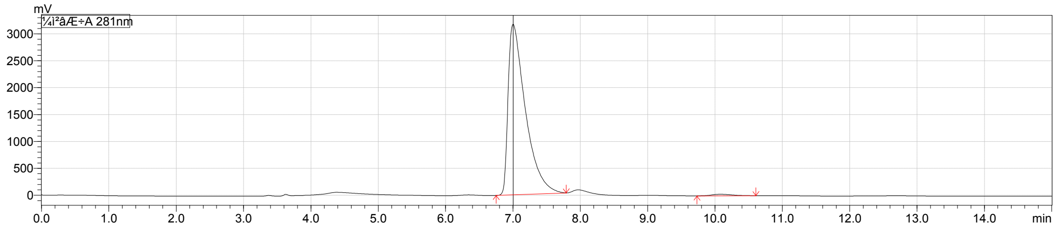

No.	Rel. Time	Area	Height	Rel. Area
1	7.002	56662060	3174185	98.909
2	10.081	625093	29753	1.091
Total:		57287153	3203938	100.000

V：^{1}H and ^{13}C NMR Spectra of Compounds

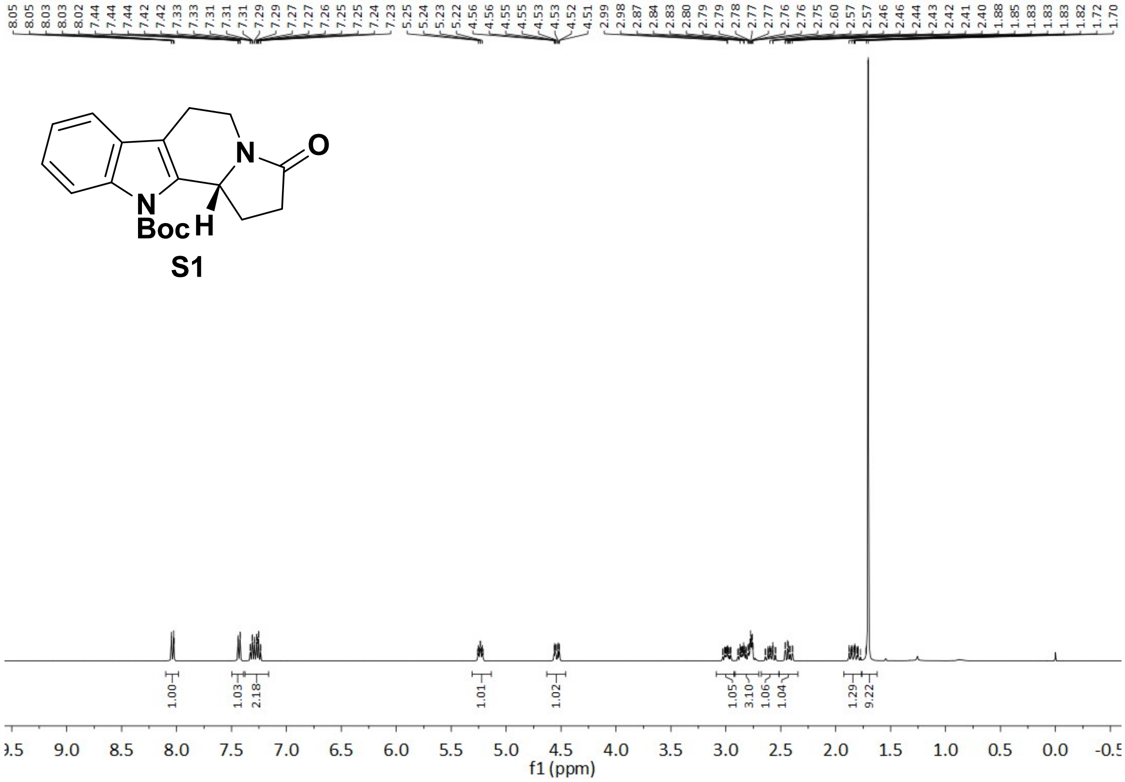

^{1}H NMR spectrum of compound **S1**

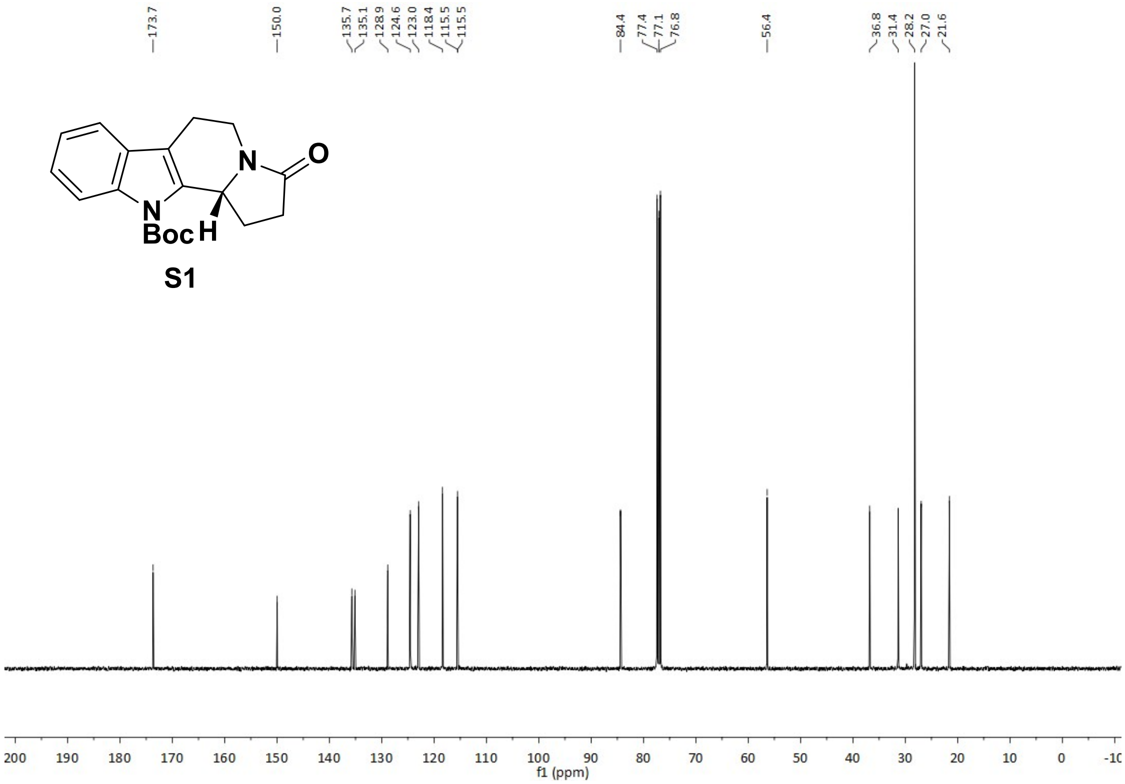

^{13}C NMR spectrum of compound **S1**

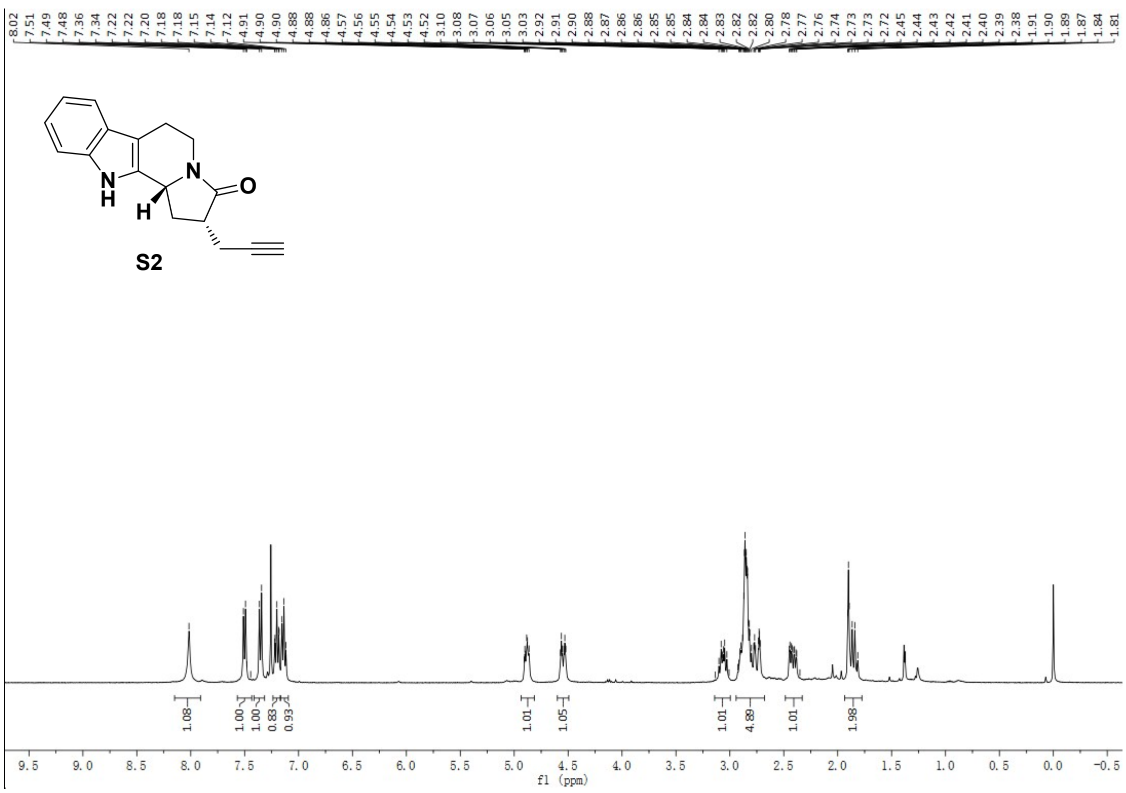

^{1}H NMR spectrum of compound **S2**

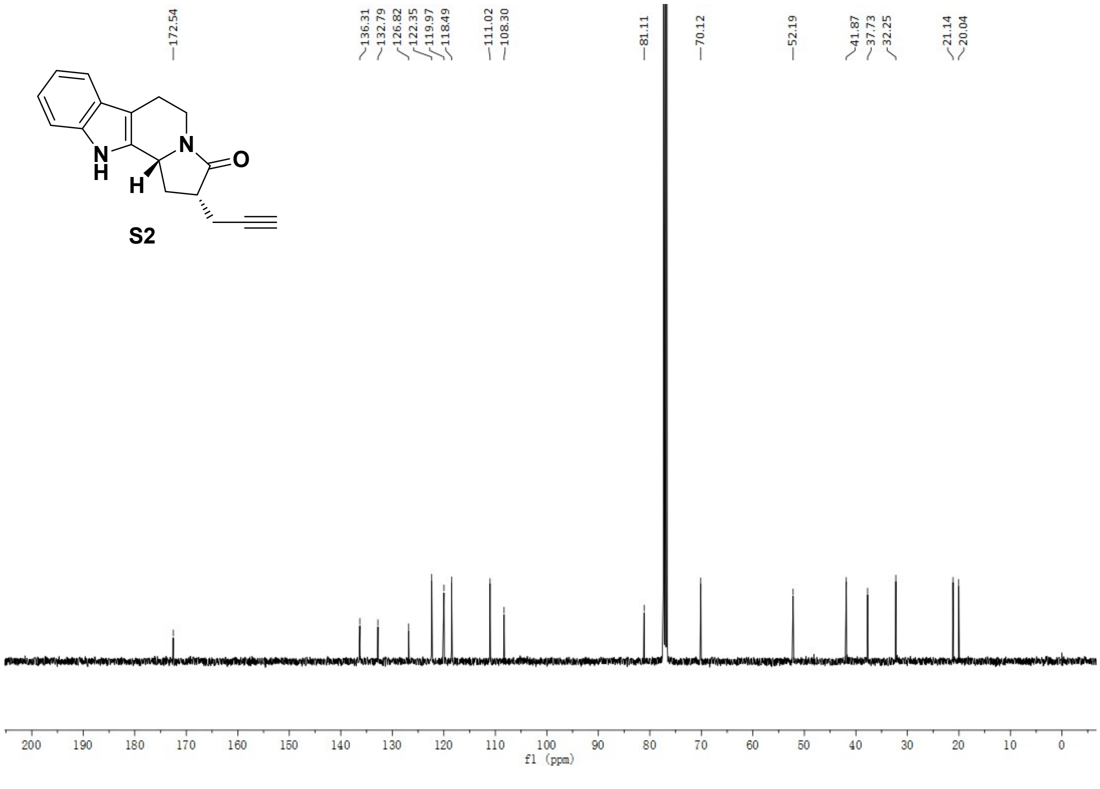

^{13}C NMR spectrum of compound **S2**

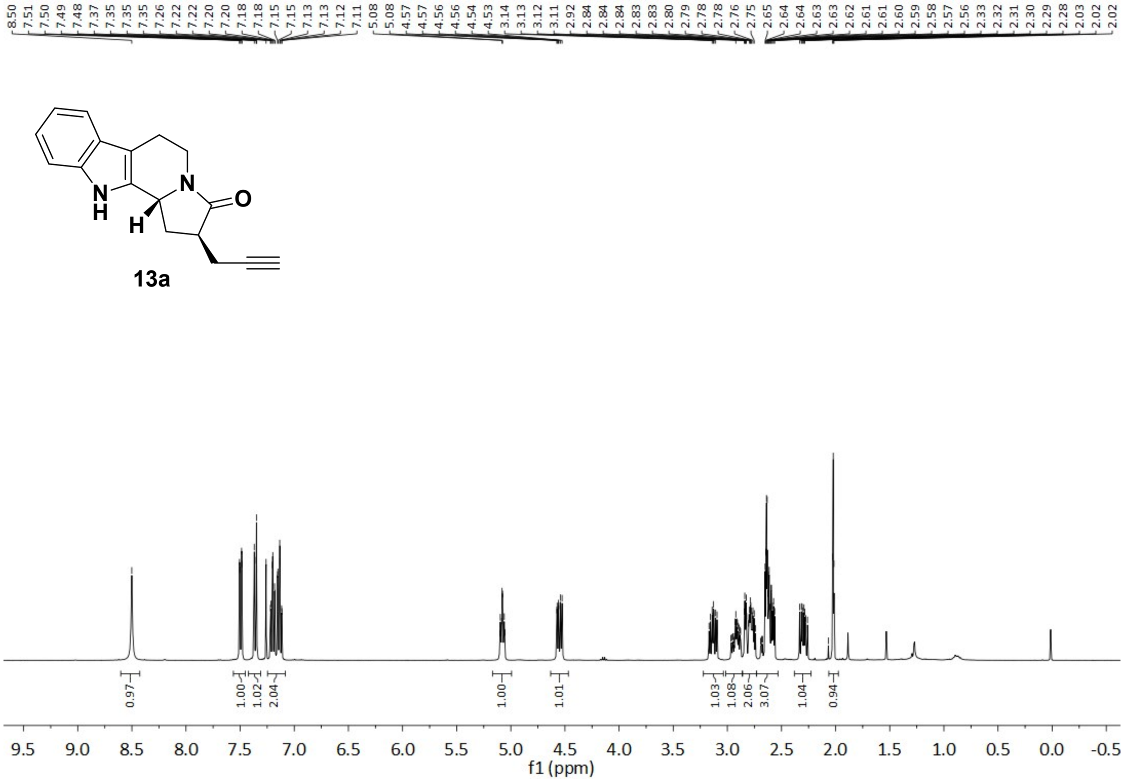

^{1}H NMR spectrum of compound **13a**

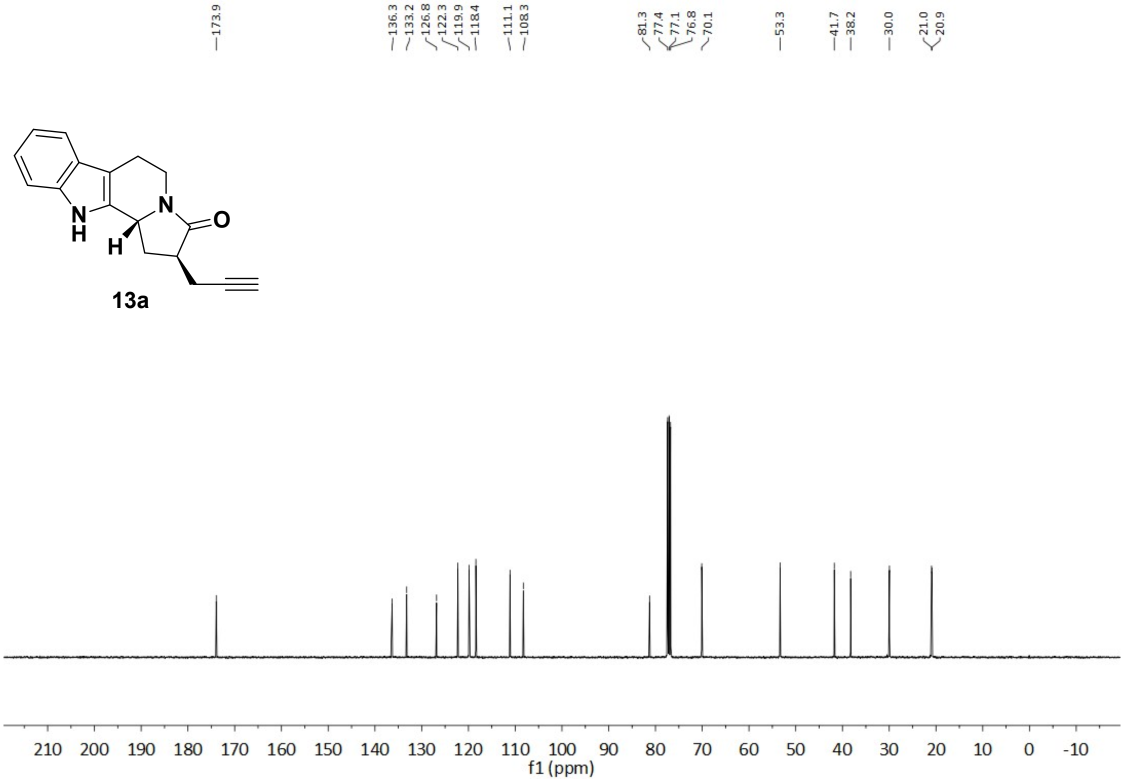

^{13}C NMR spectrum of compound **13a**

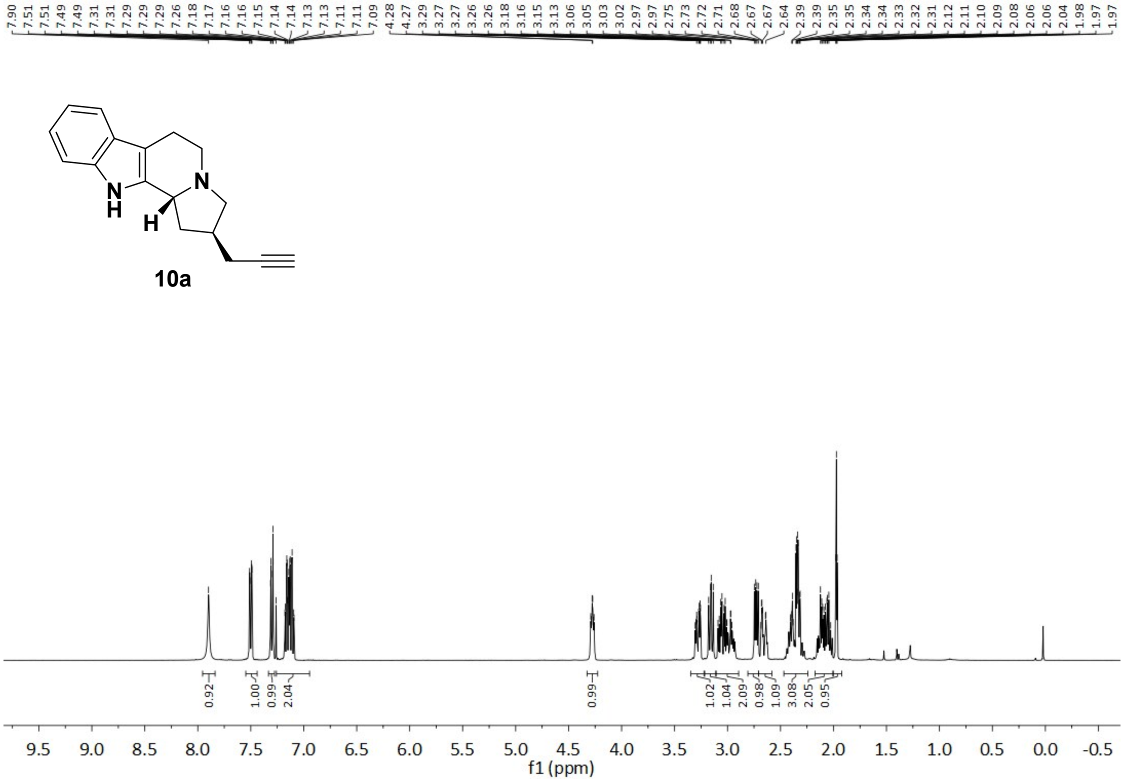

^{1}H NMR spectrum of compound **10a**

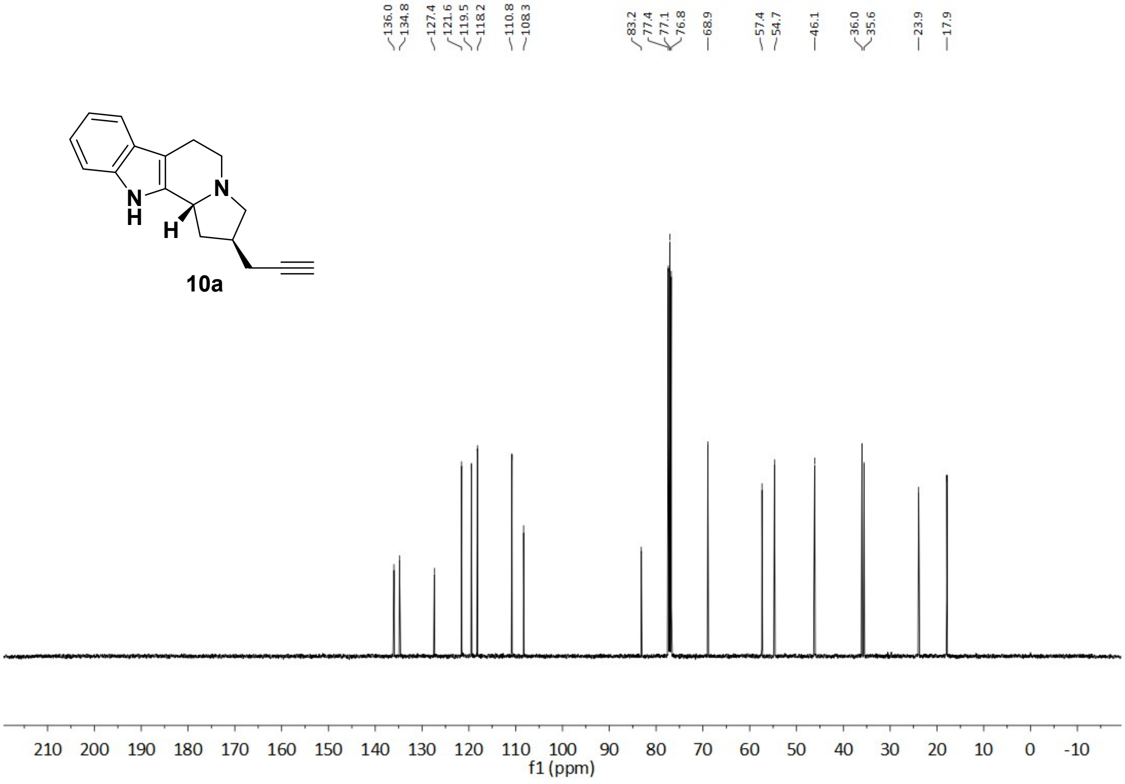

^{13}C NMR spectrum of compound **10a**

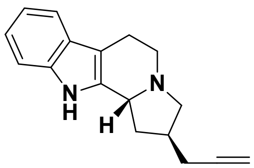

10a

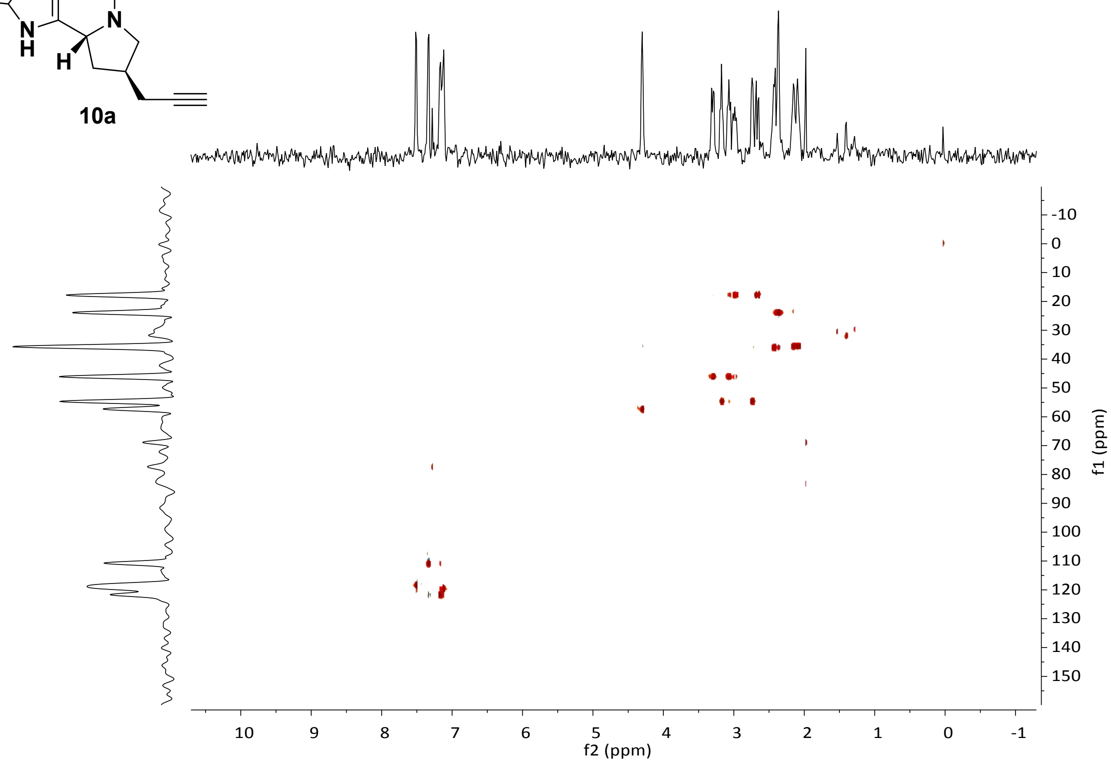

HMQC spectrum of compound **10a**

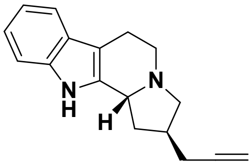

10a

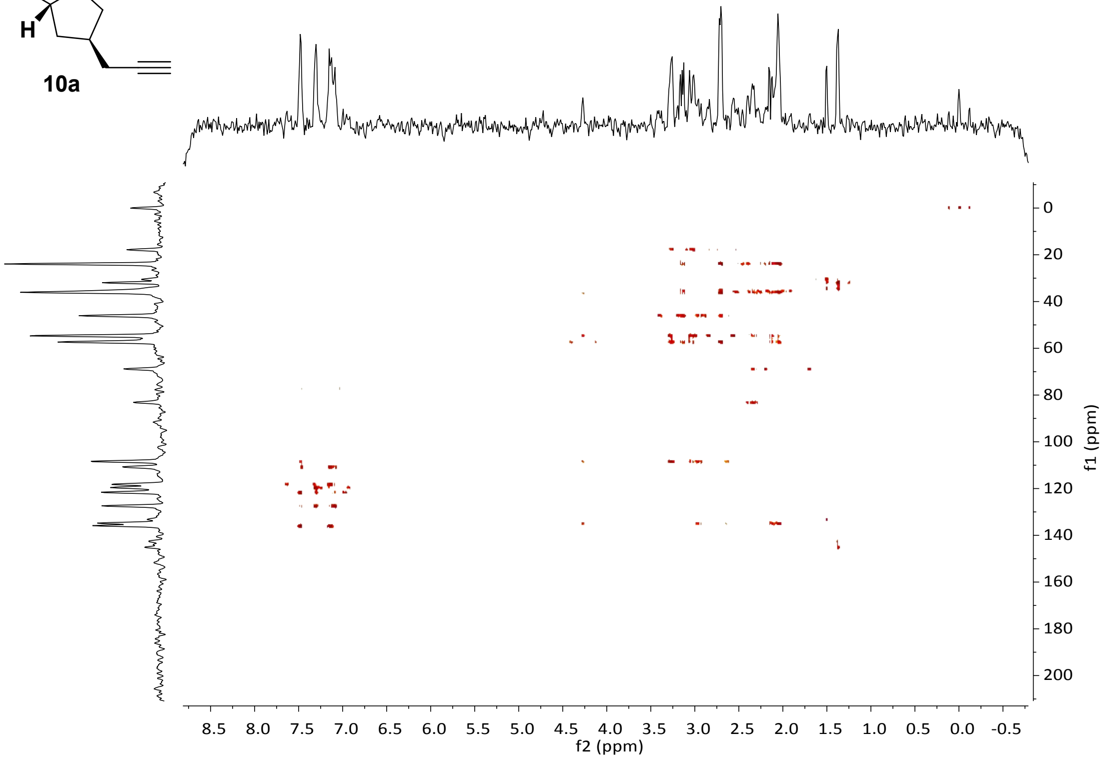

HMQC spectrum of compound **10a**

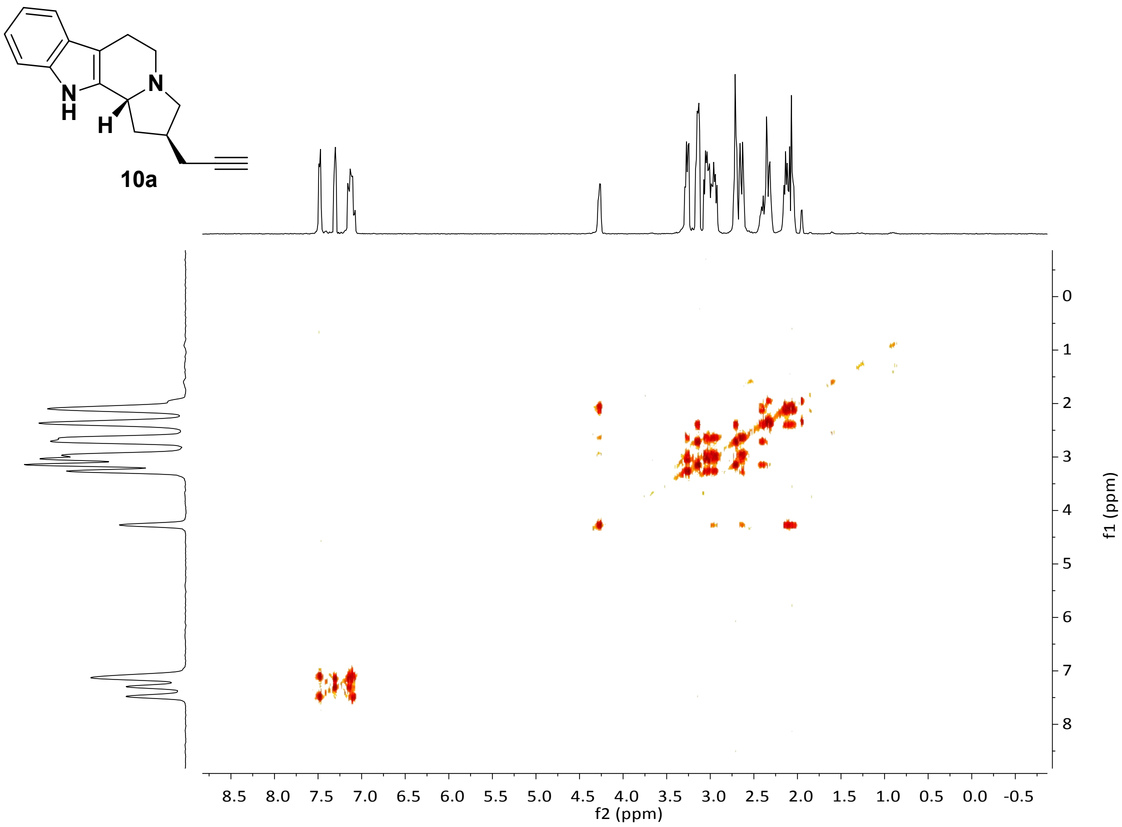

COSY spectrum of compound **10a**

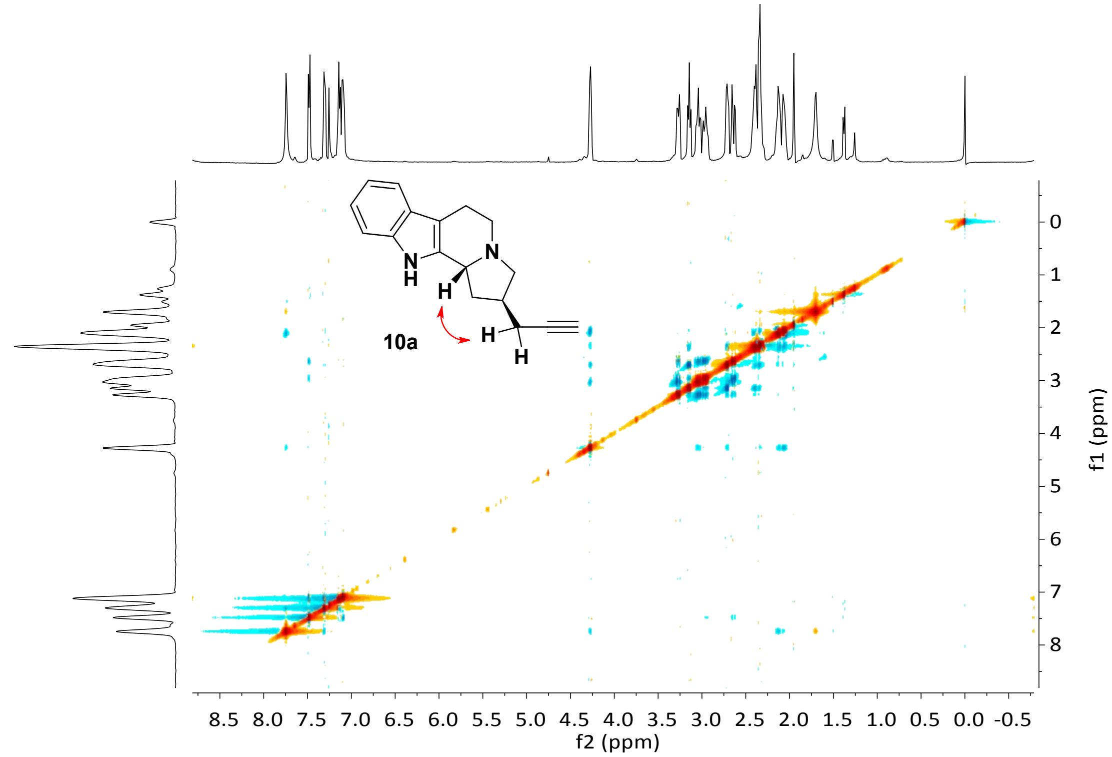

NOESY spectrum of compound **10a**

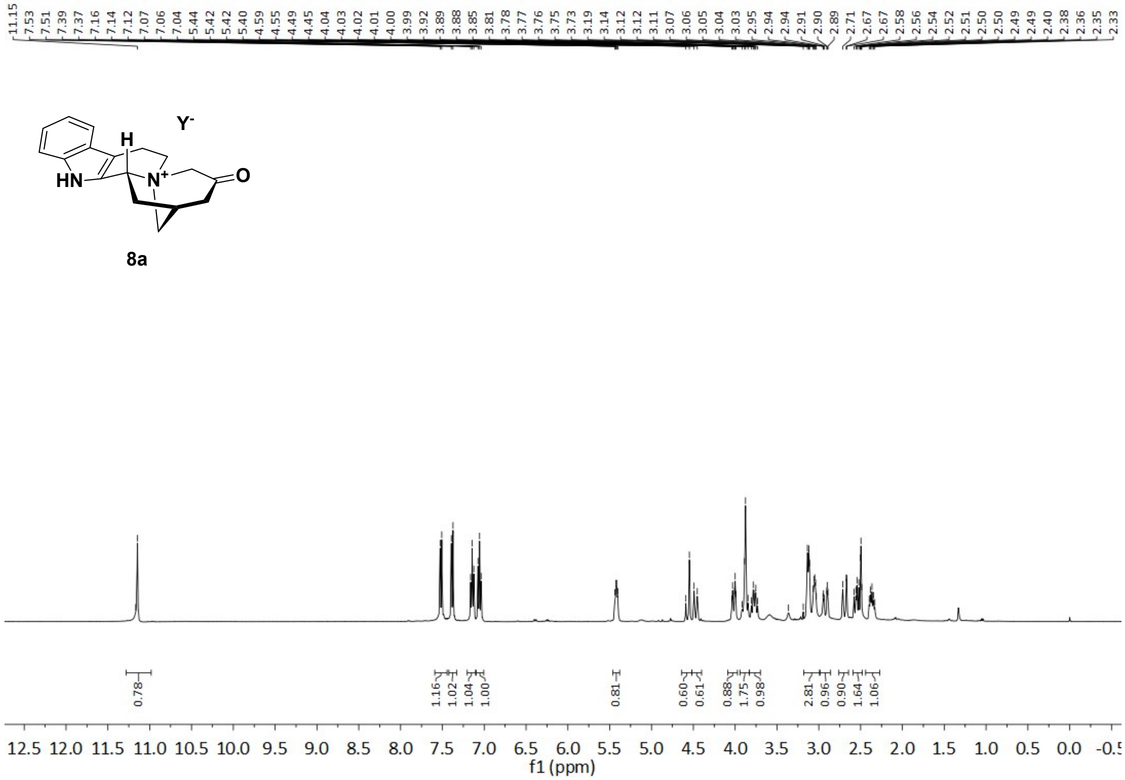

^{1}H NMR spectrum of compound **8a**

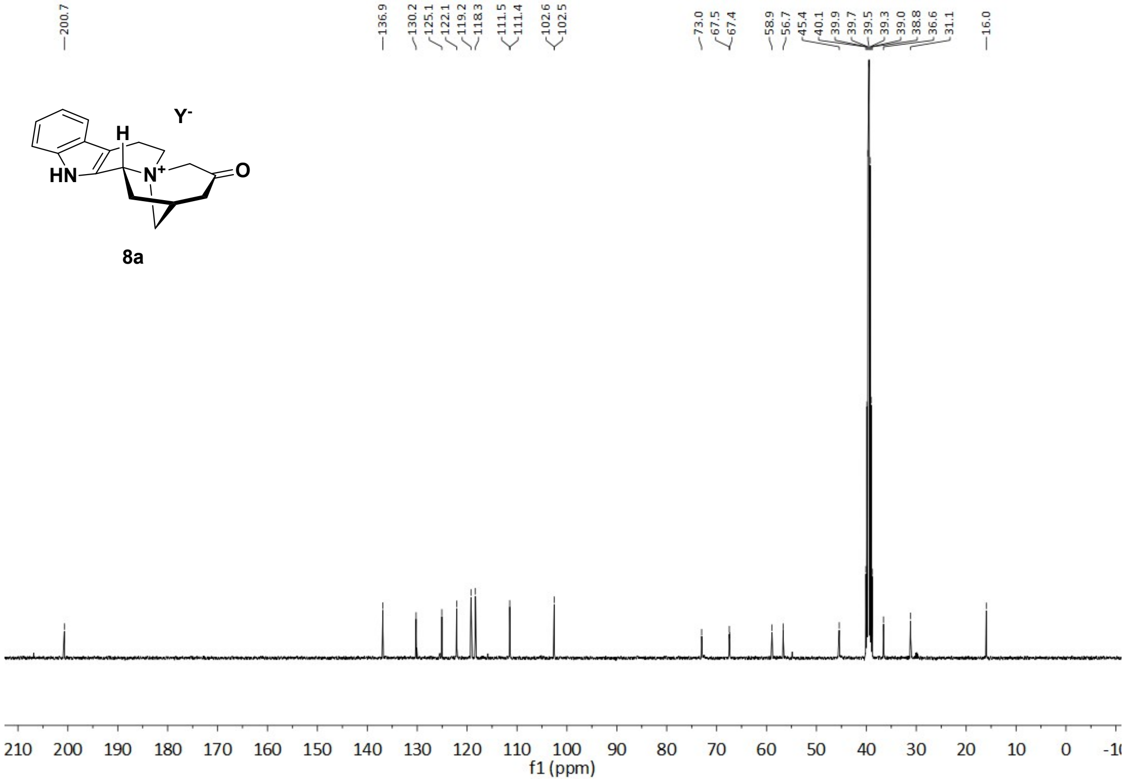

^{13}C NMR spectrum of compound **8a**

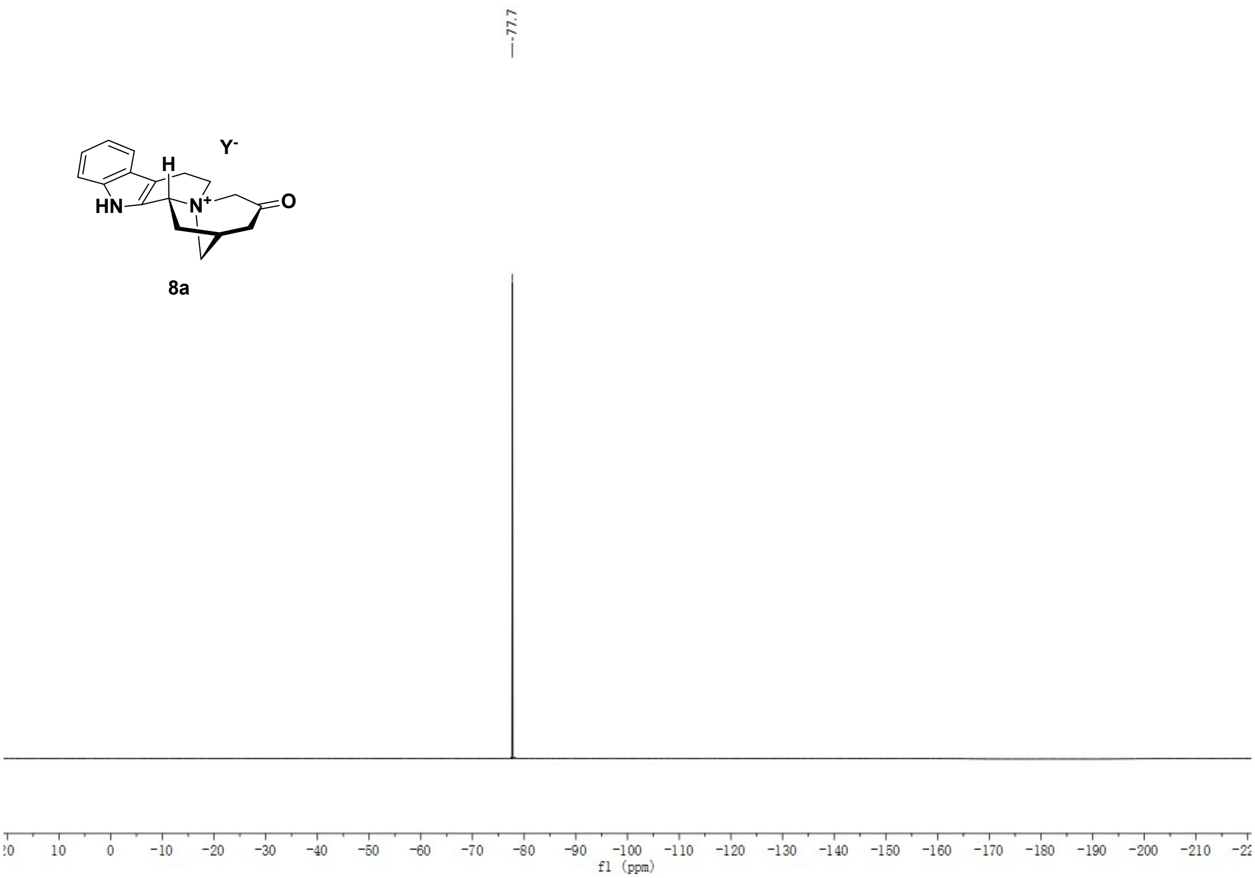

^{19}F NMR spectrum of compound **8a**

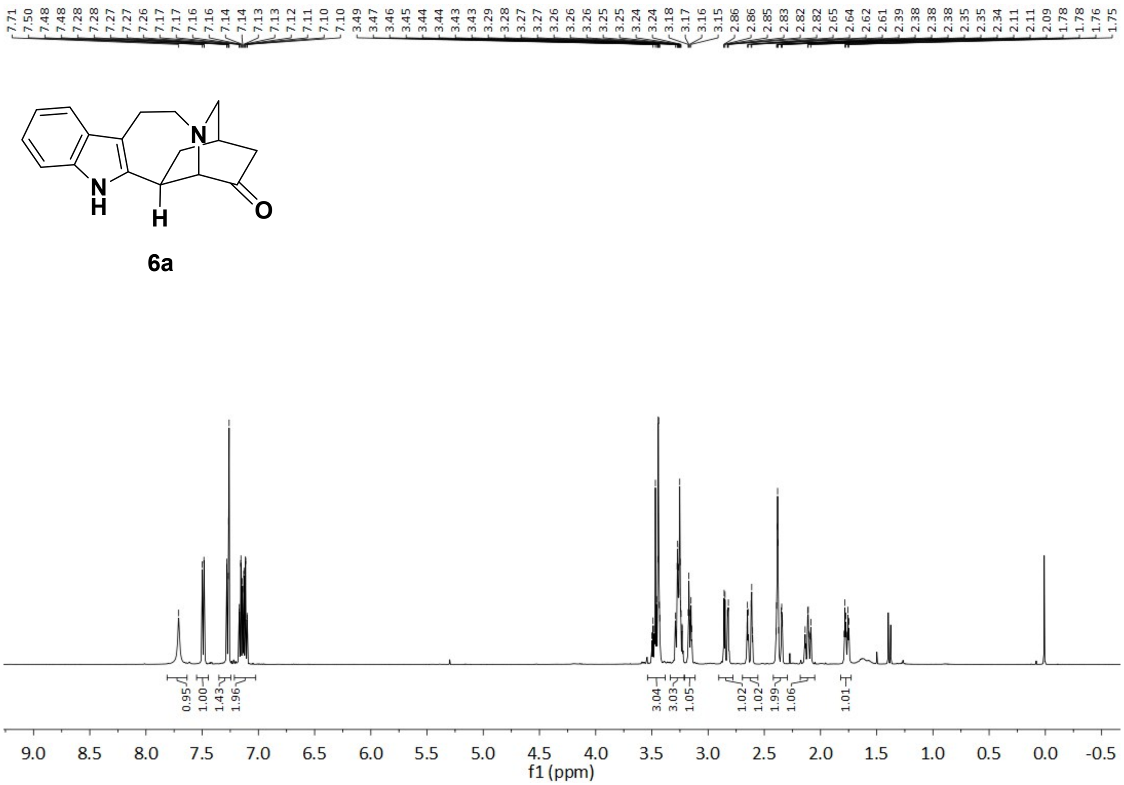

^{1}H NMR spectrum of compound **6a**

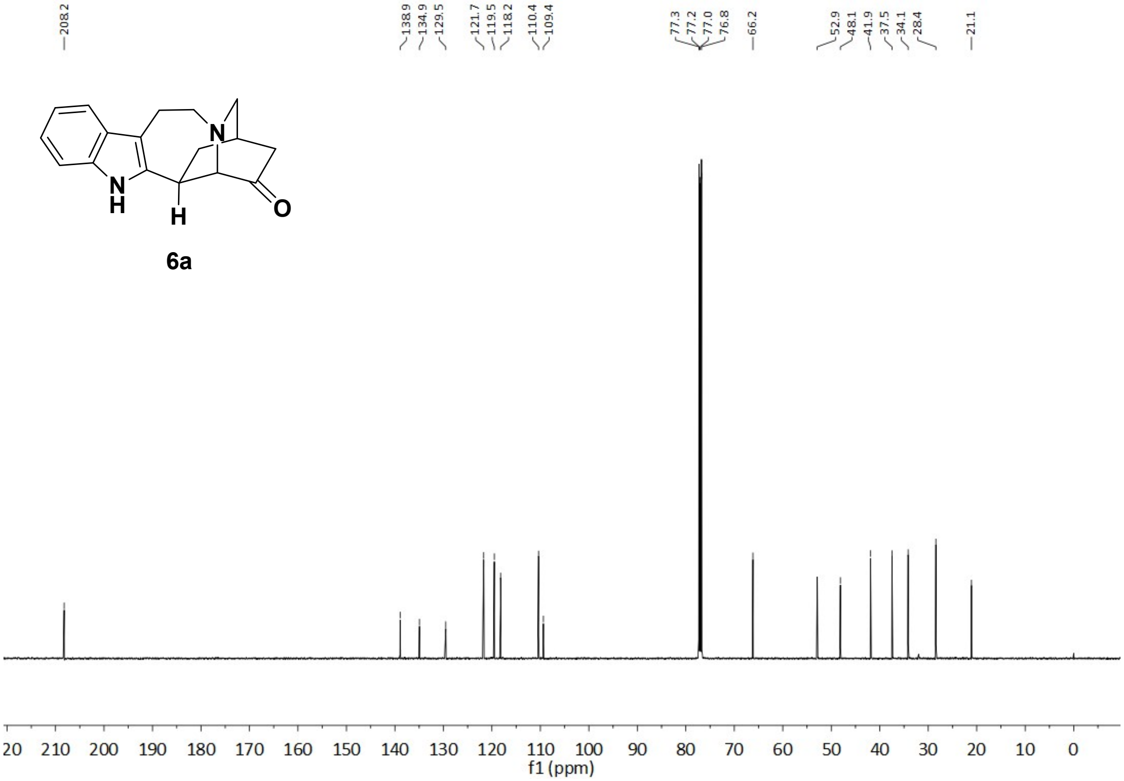

^{13}C NMR spectrum of compound **6a**

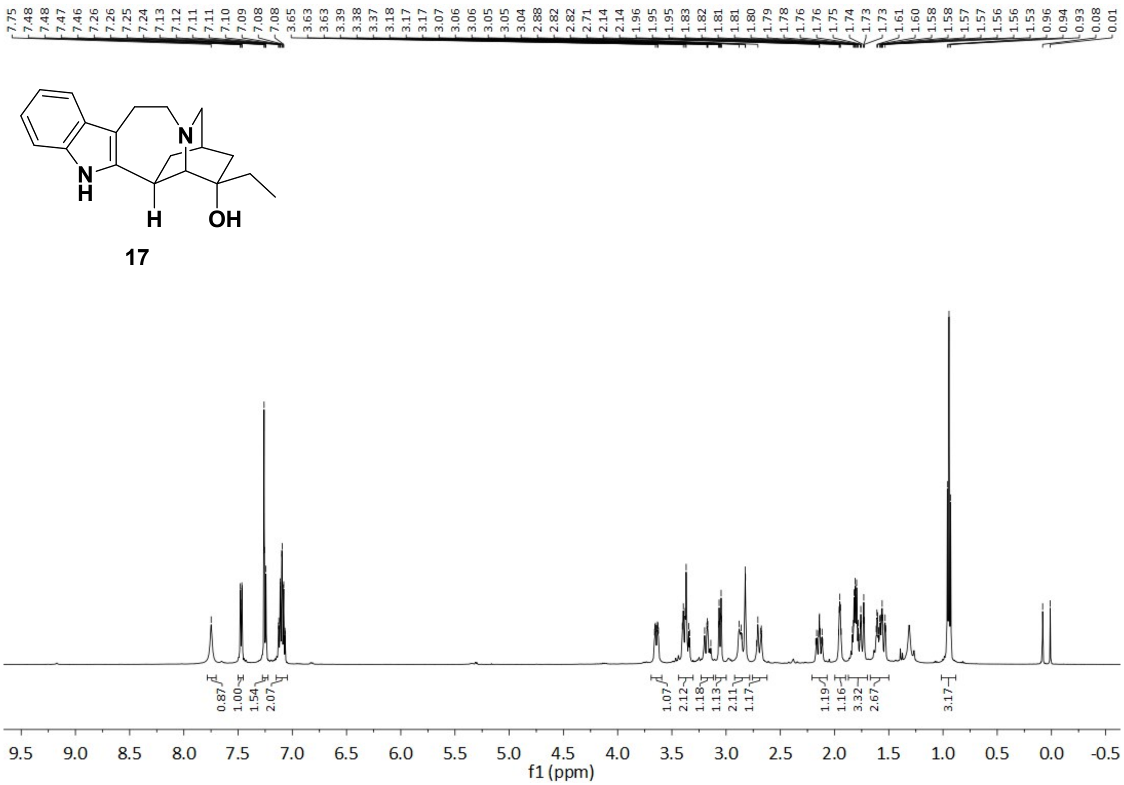

^{1}H NMR spectrum of compound **17**

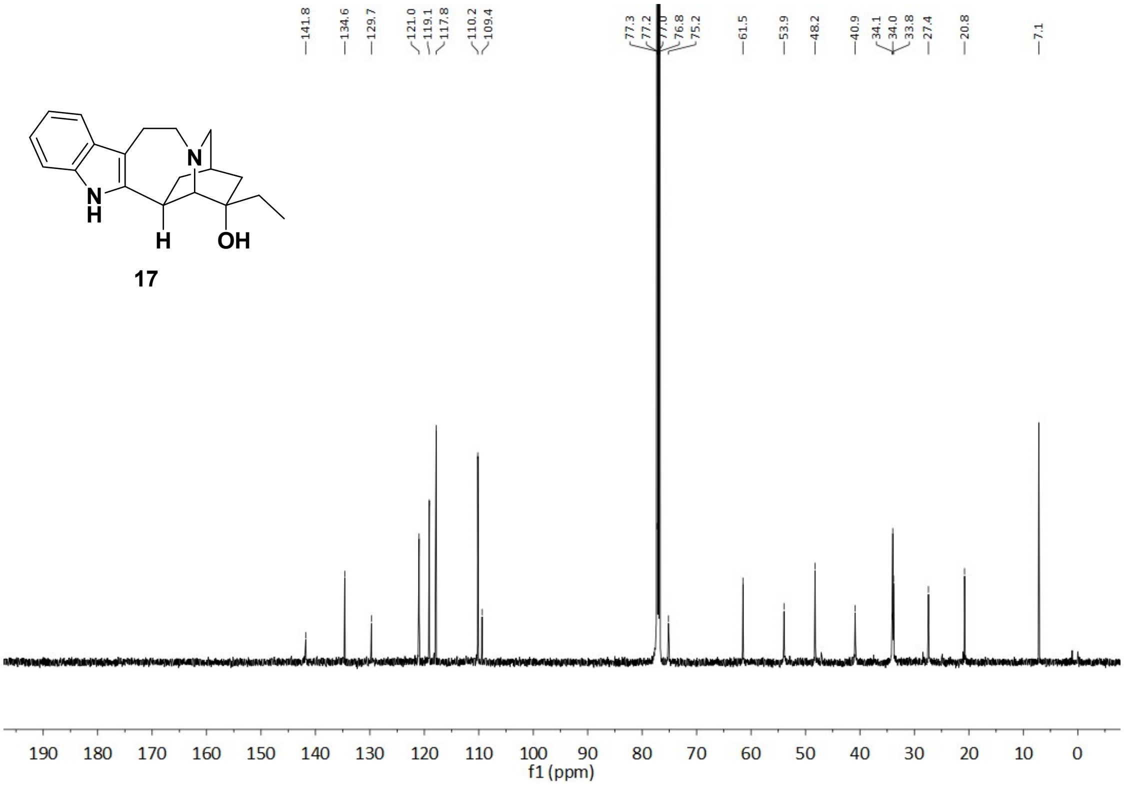

^{13}C NMR spectrum of compound **17**

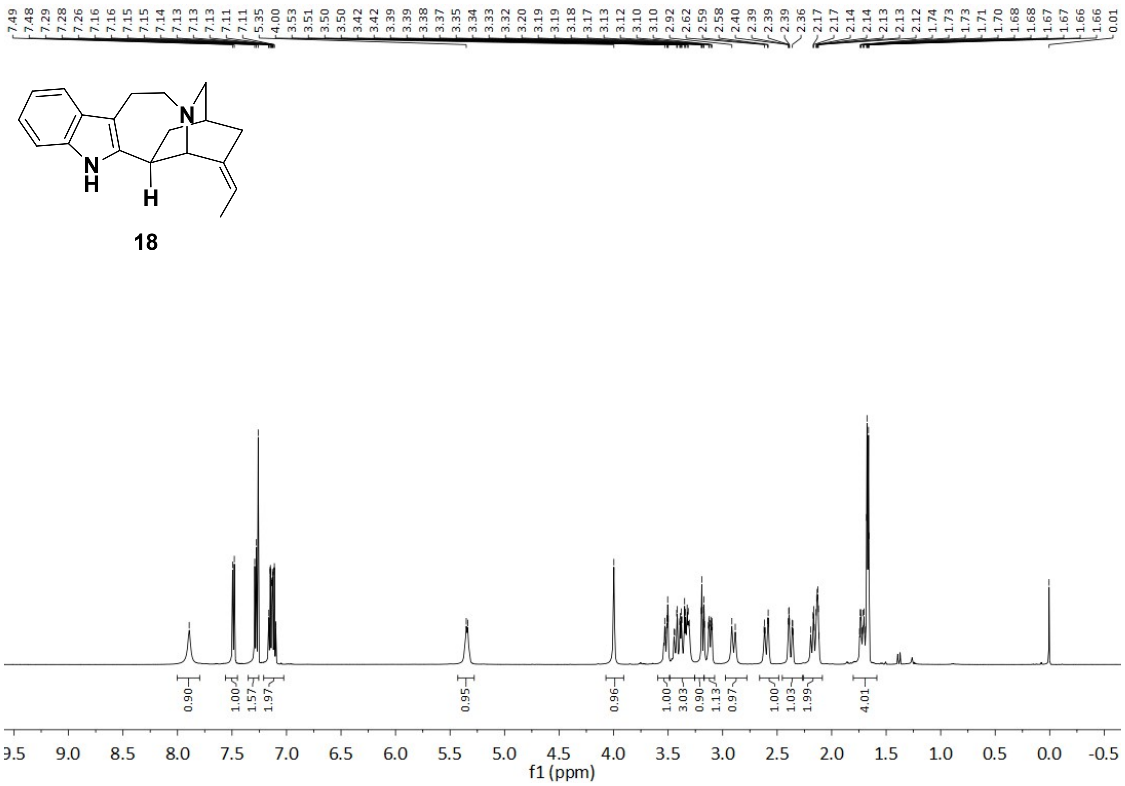

^{1}H NMR spectrum of compound **18**

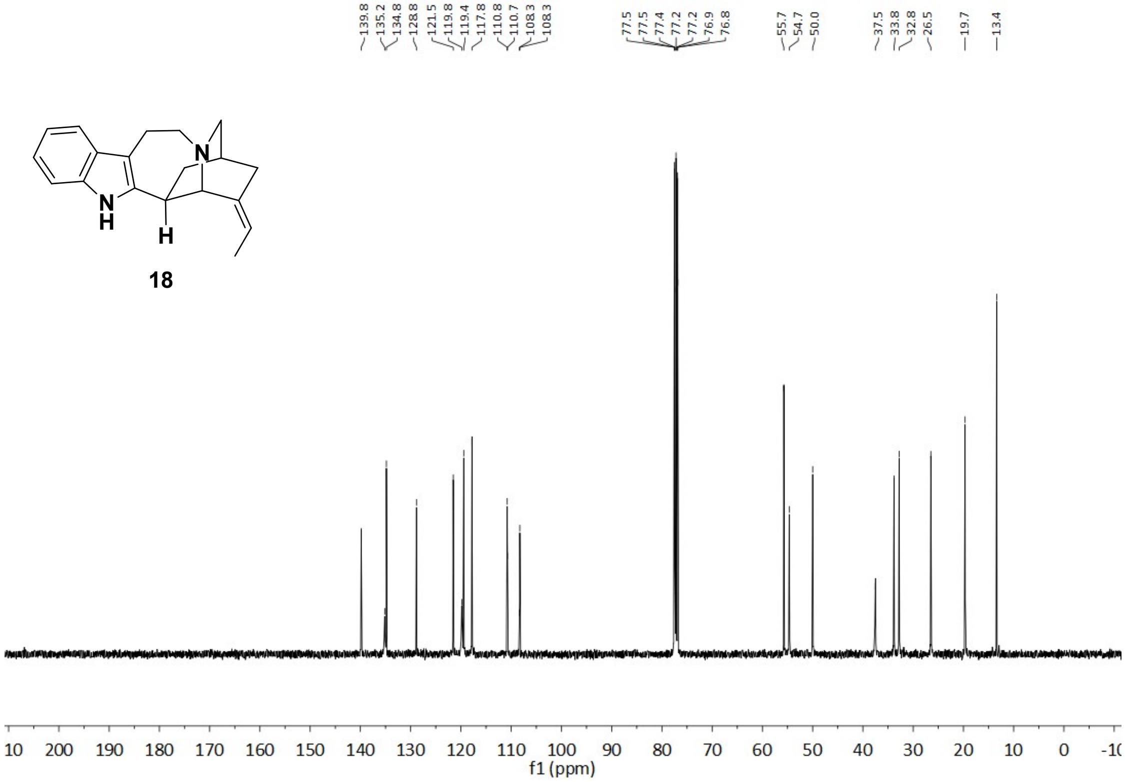

^{13}C NMR spectrum of compound **18**

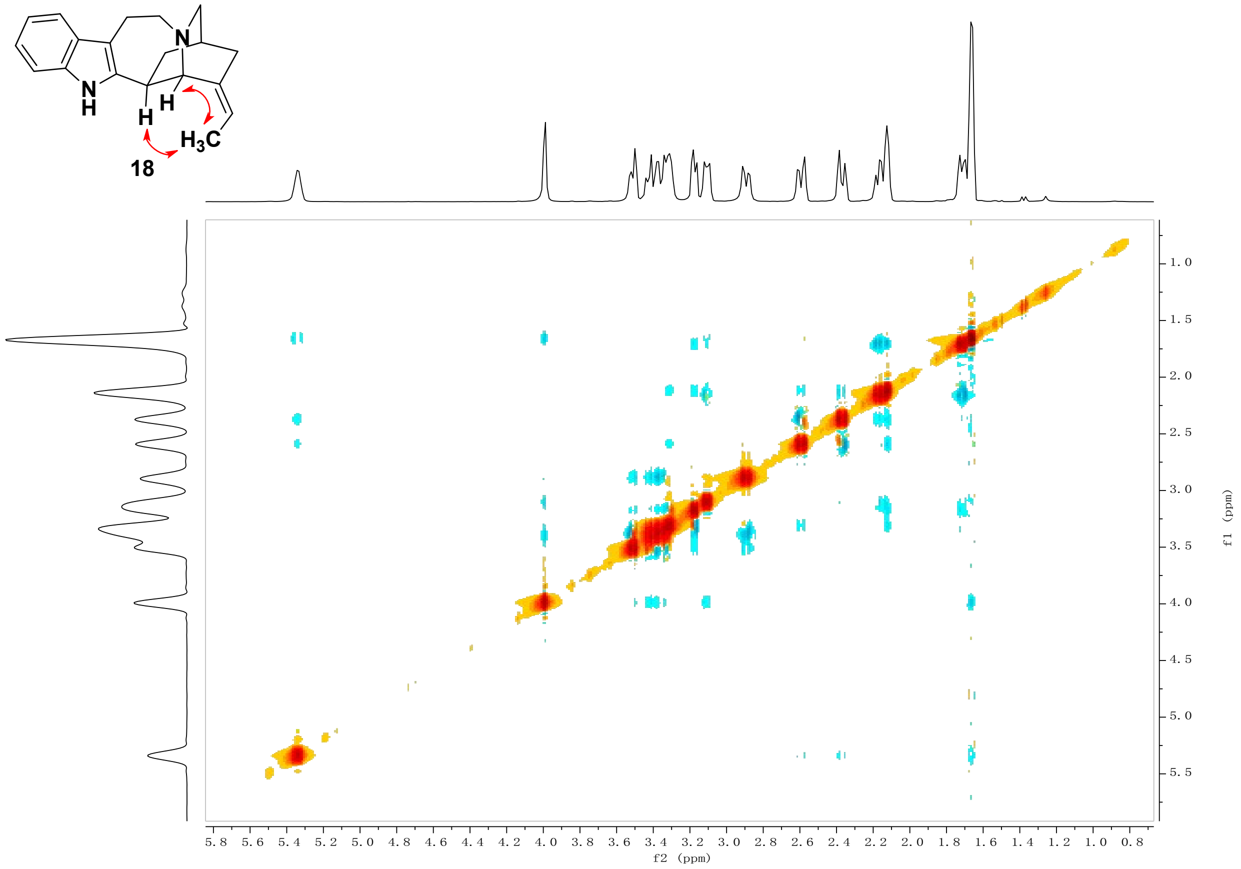

^{1}H-^{1}H NOESY spectrum of compound **18**

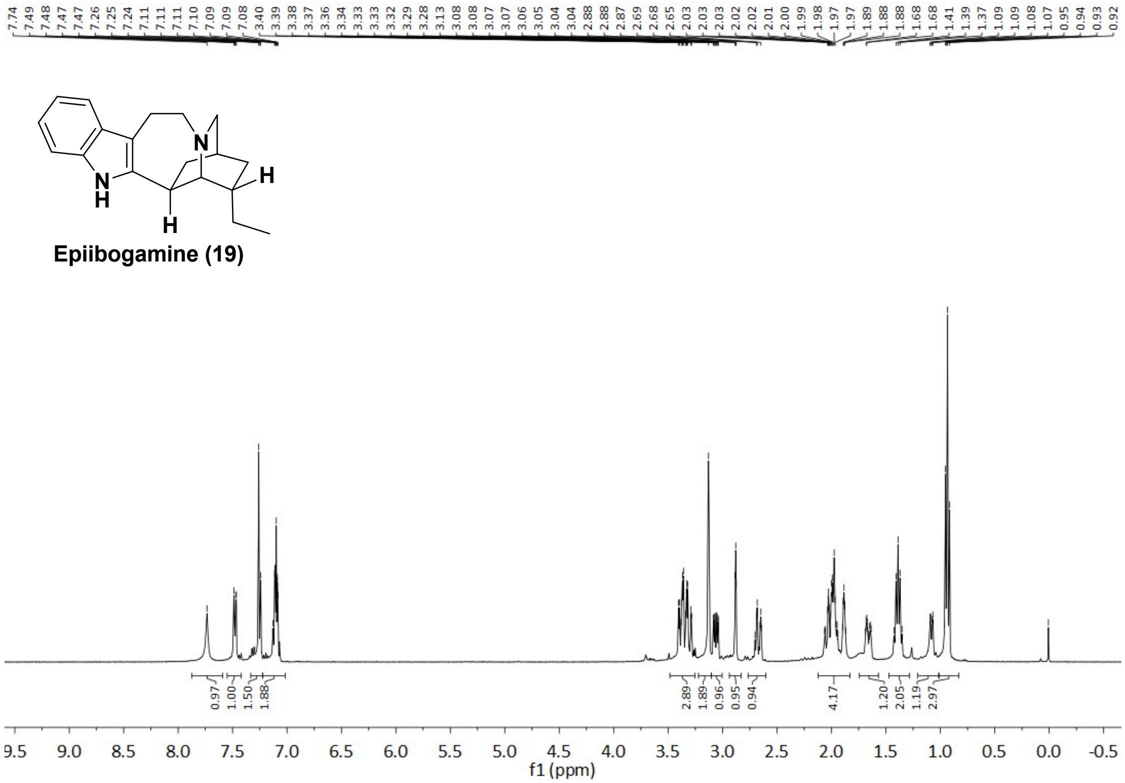

^{1}H NMR spectrum of compound **19**

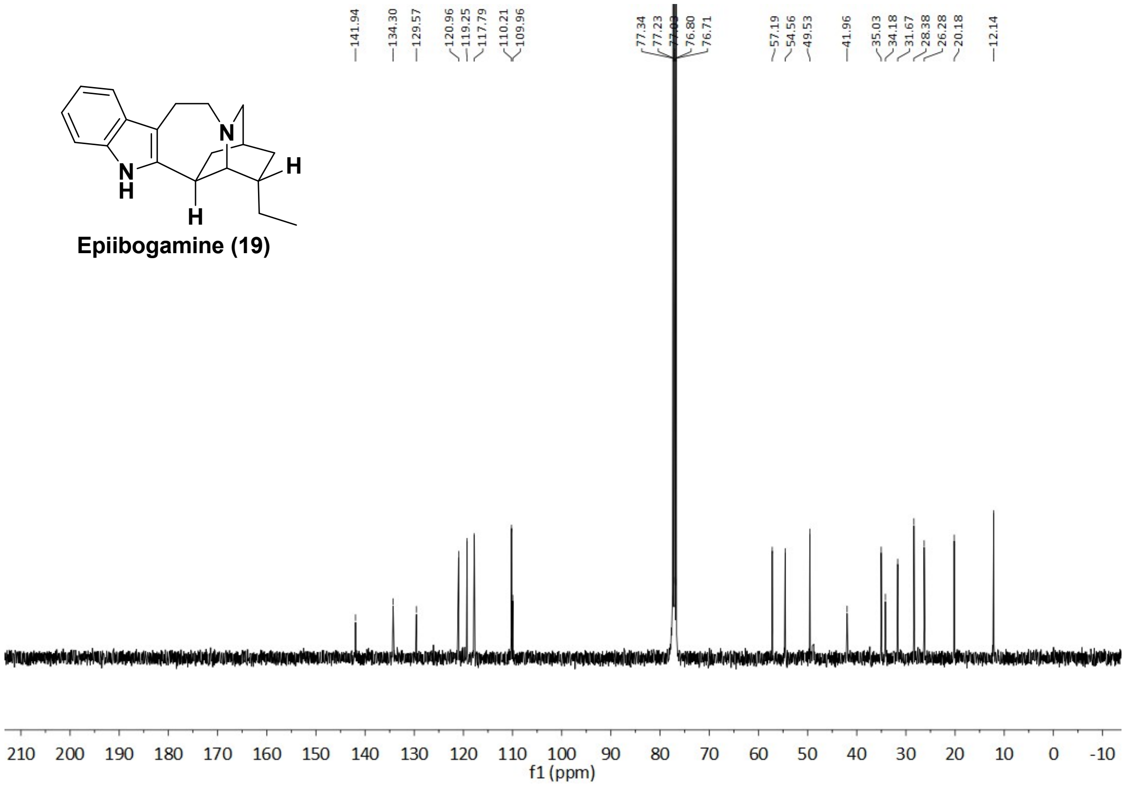

^{13}C NMR spectrum of compound **19**

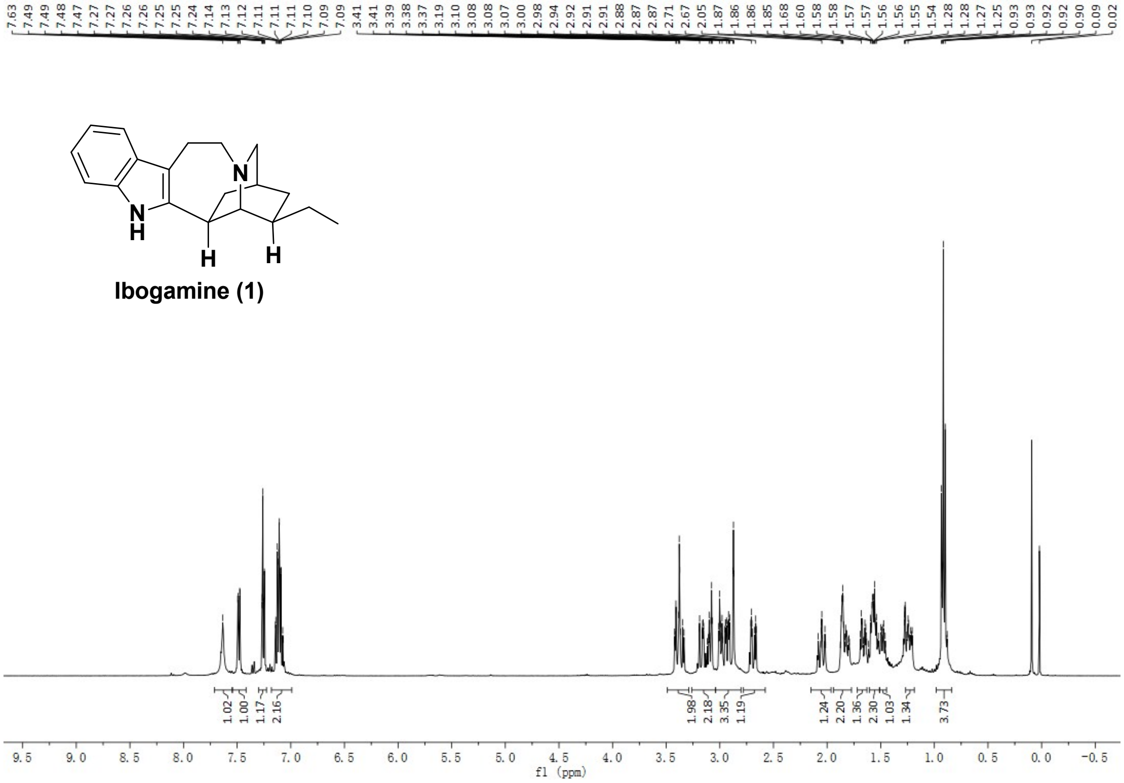

^{1}H NMR spectrum of compound **1**

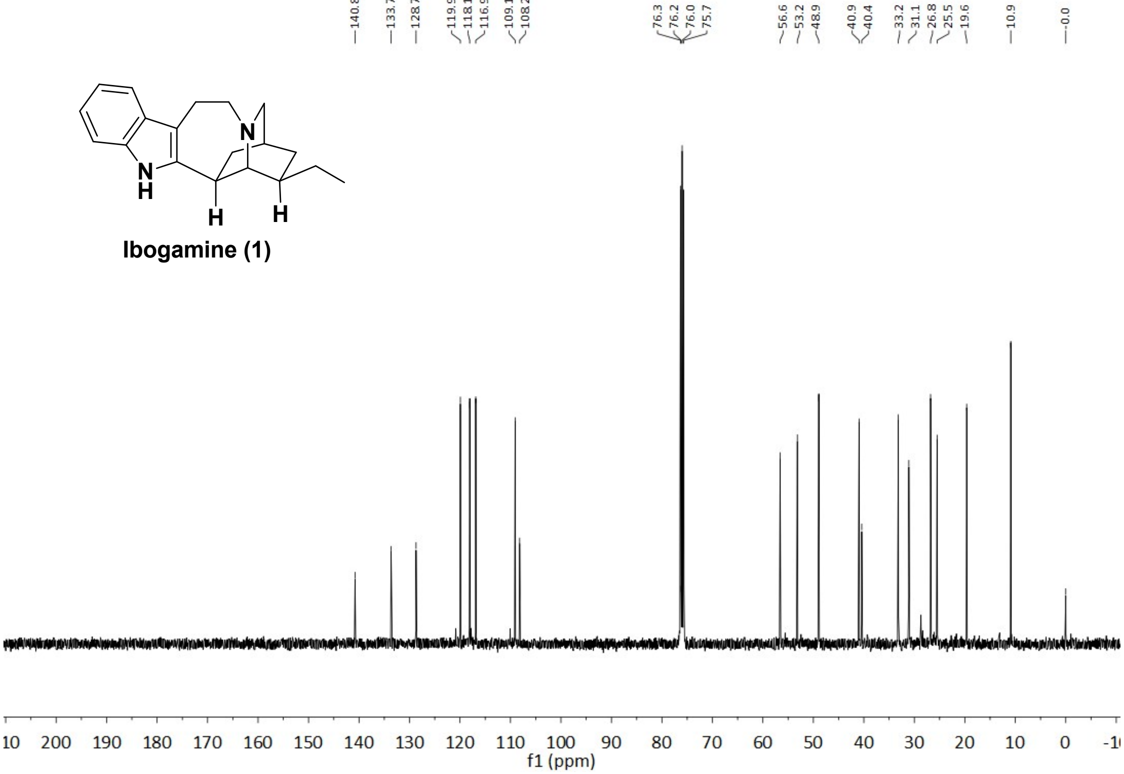

^{13}C NMR spectrum of compound **1**

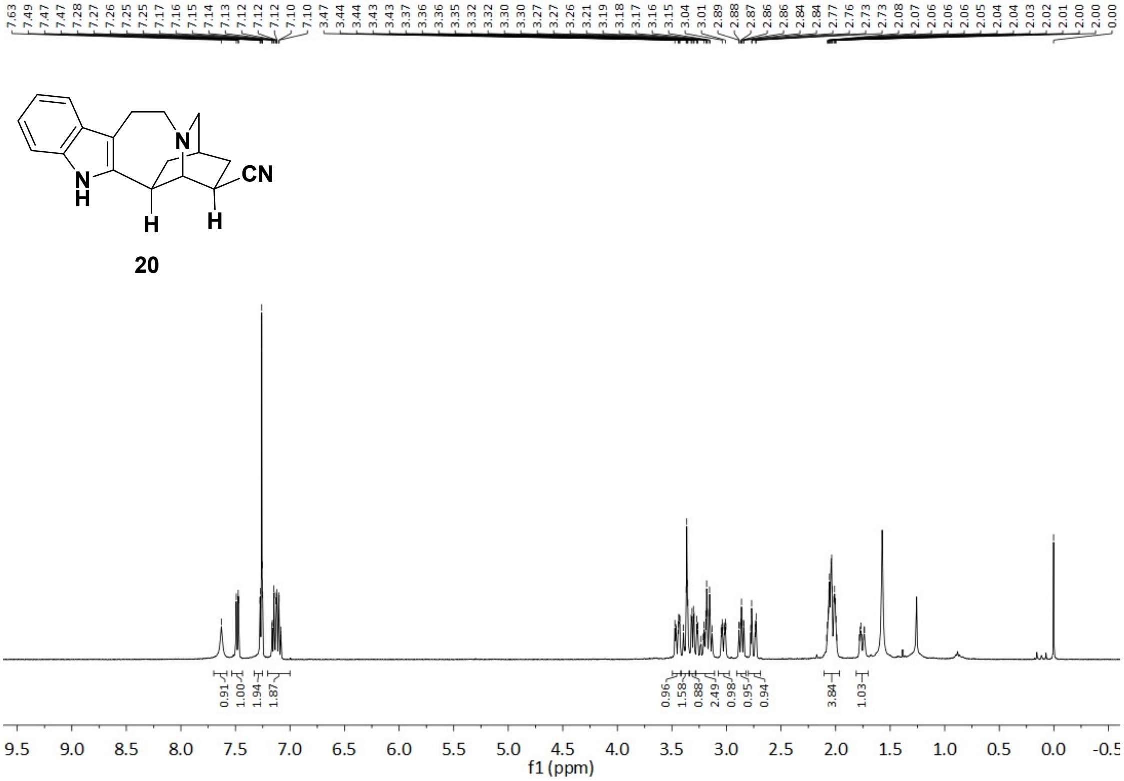

^{1}H NMR spectrum of compound **20**

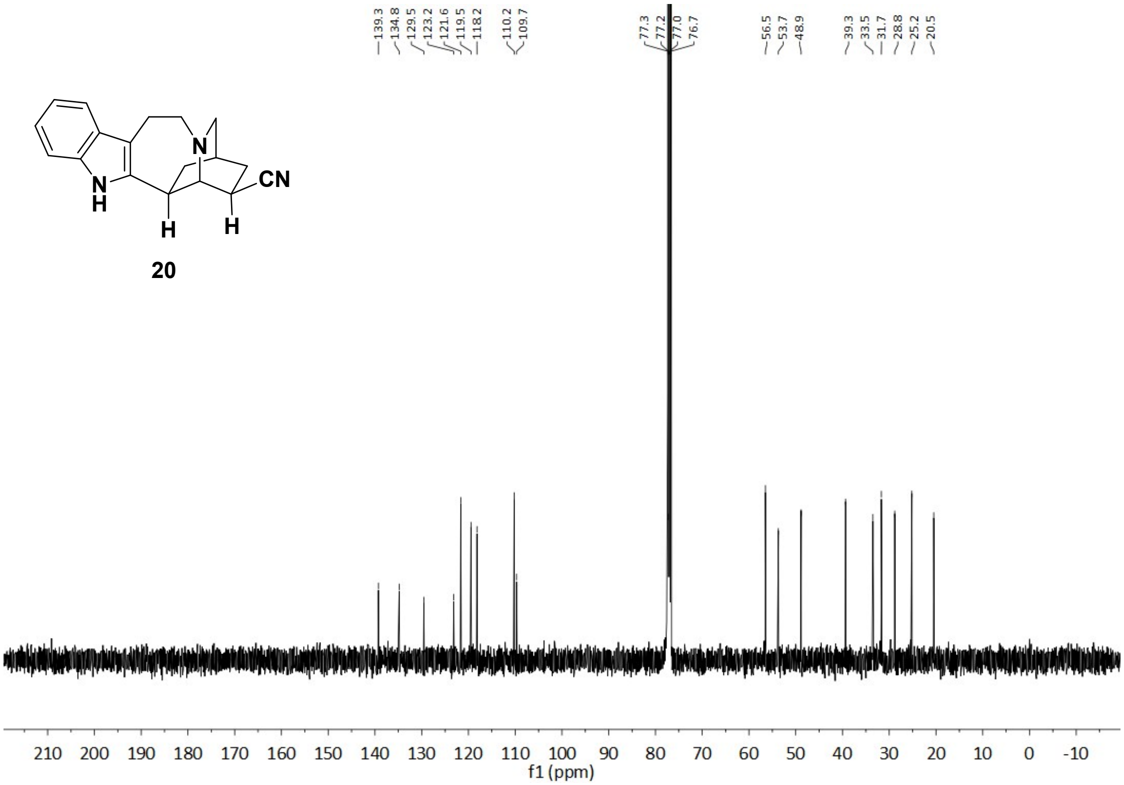

^{13}C NMR spectrum of compound **20**

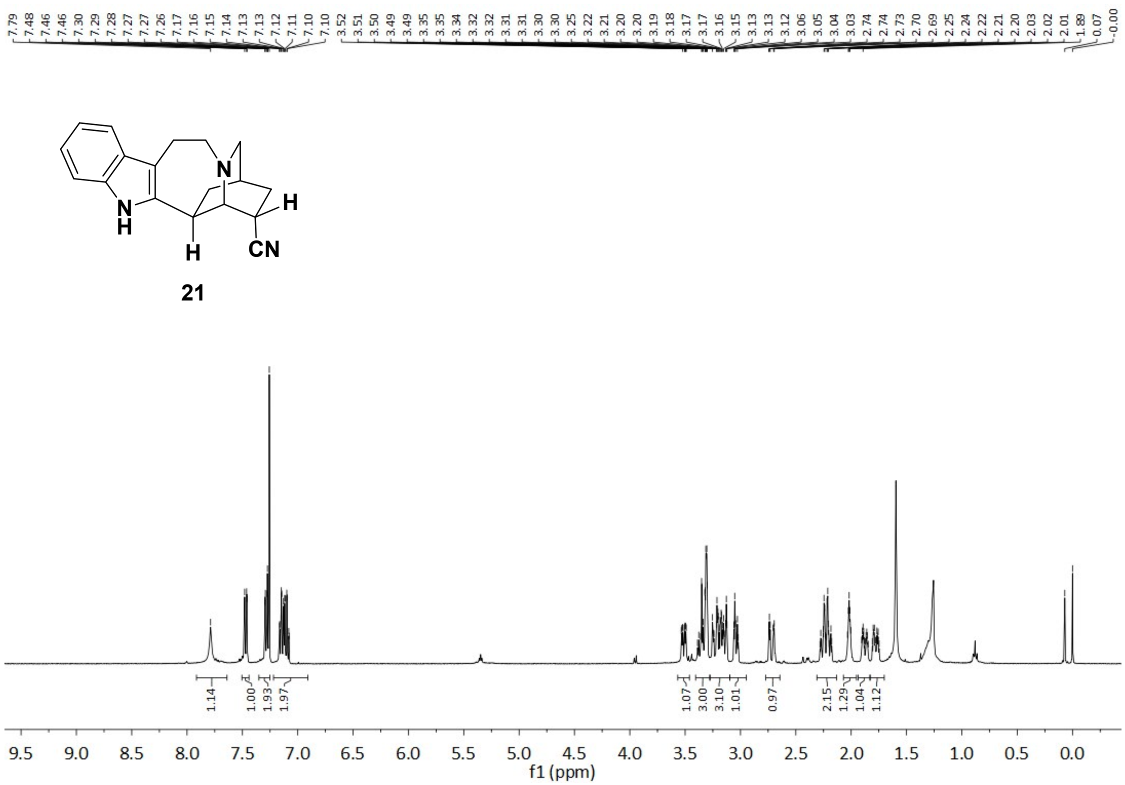

^{1}H NMR spectrum of compound **21**

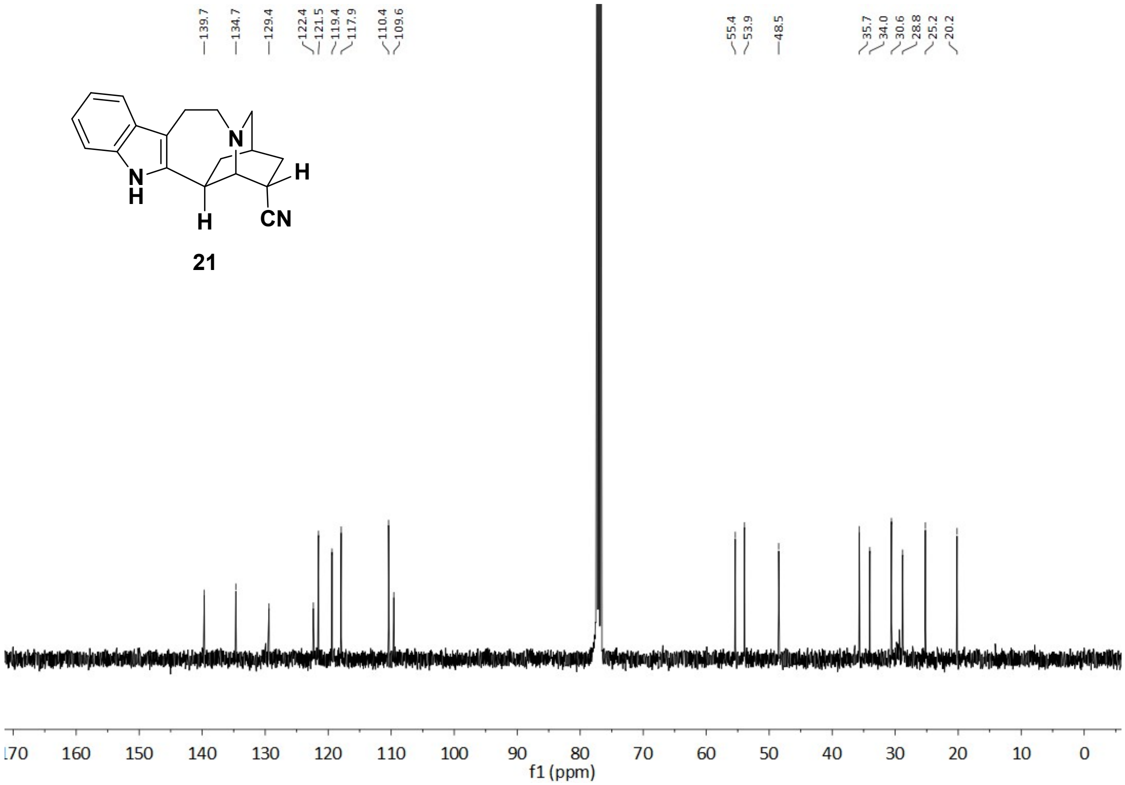

^{13}C NMR spectrum of compound **21**

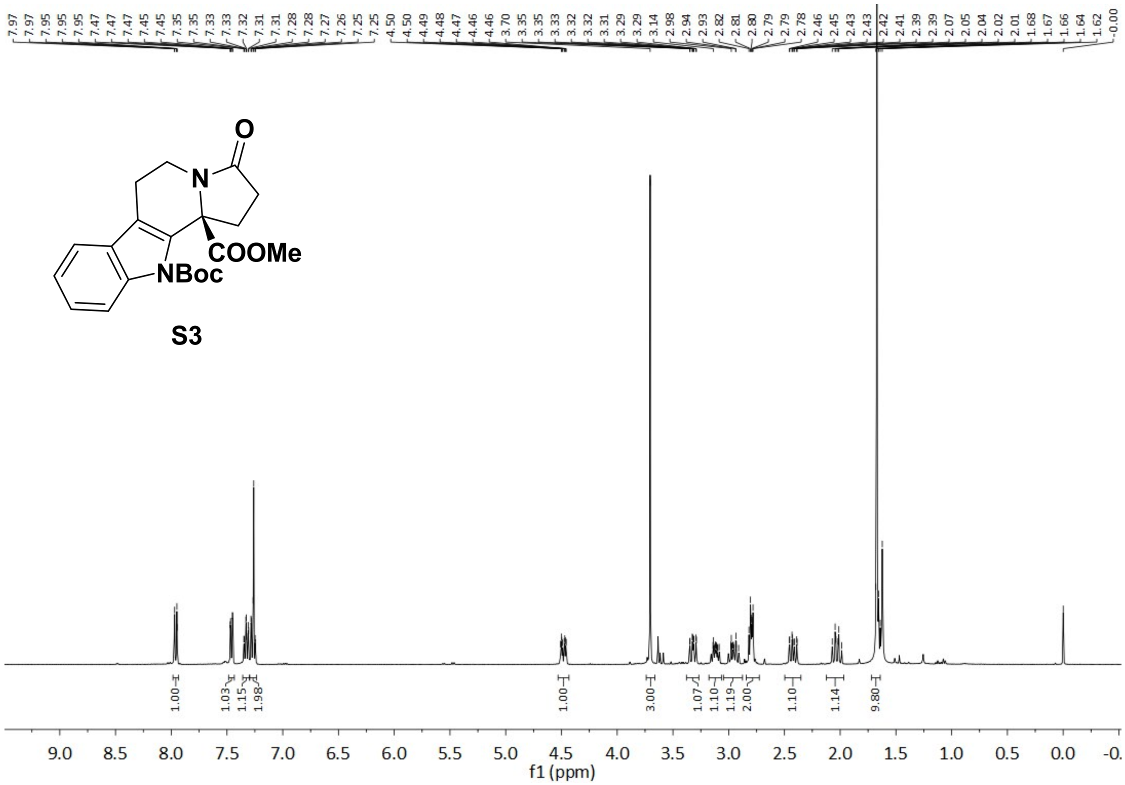

^{1}H NMR spectrum of compound **S3**

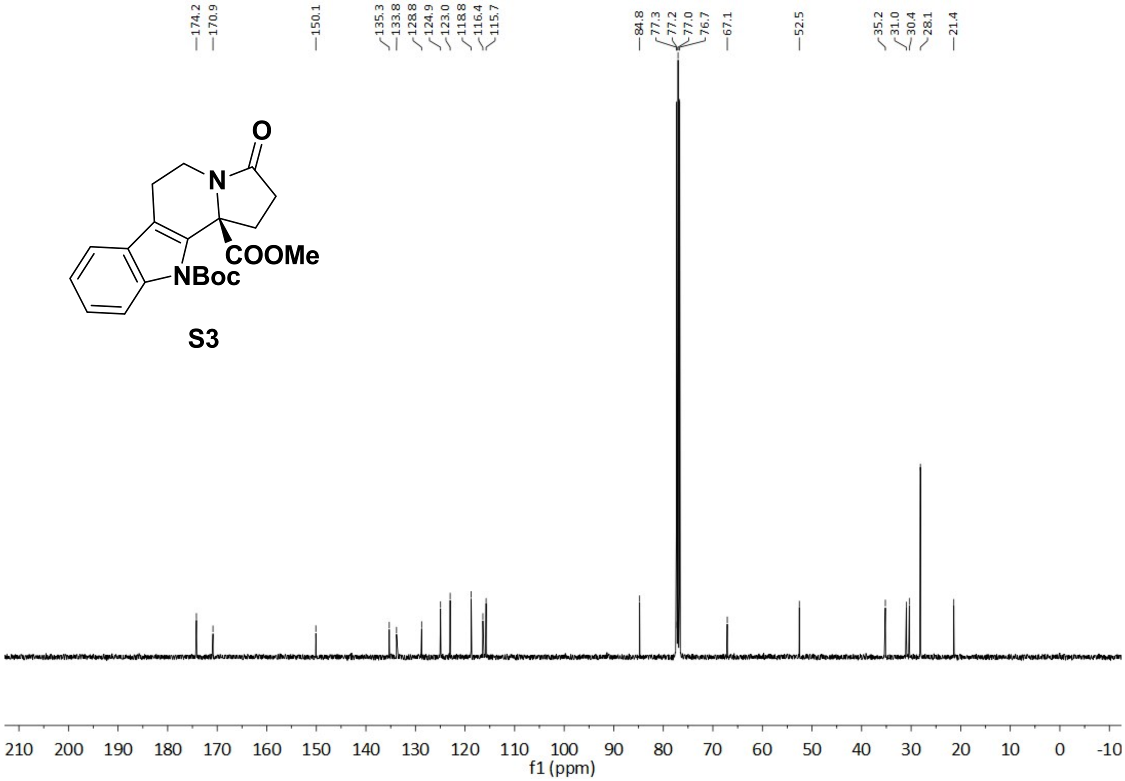

^{13}C NMR spectrum of compound **S3**

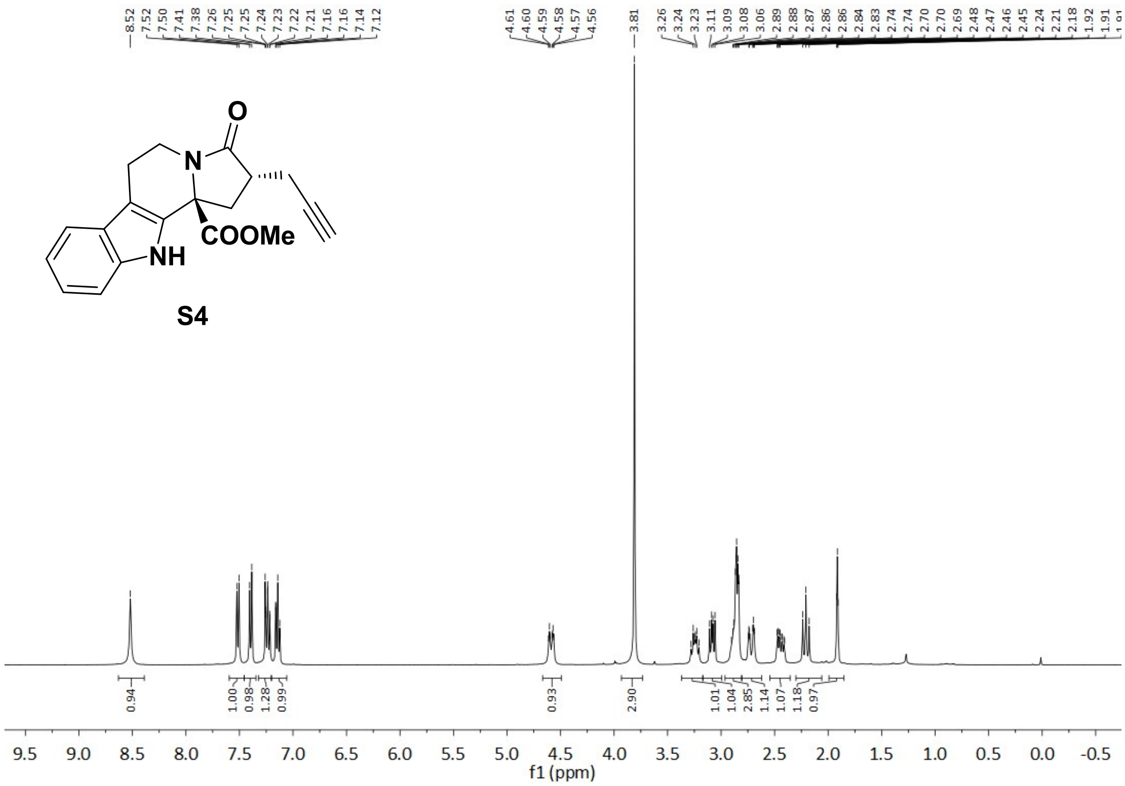

^{1}H NMR spectrum of compound **S4**

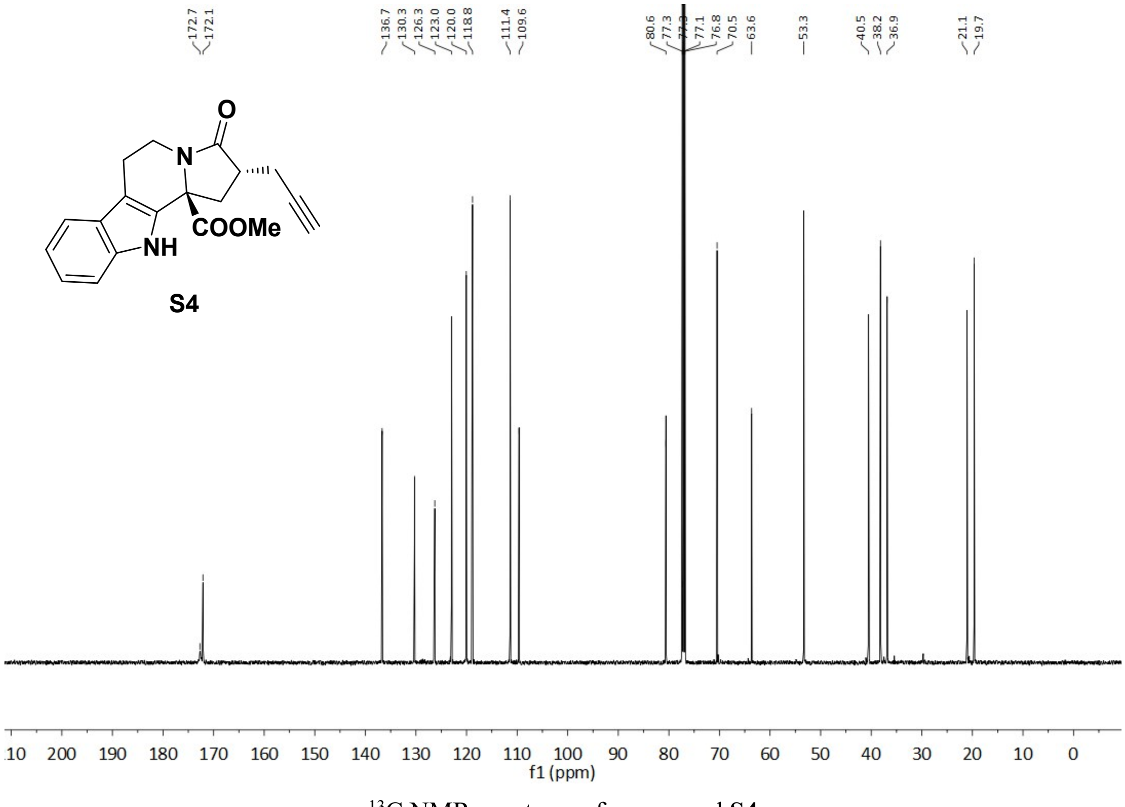

^{13}C NMR spectrum of compound **S4**

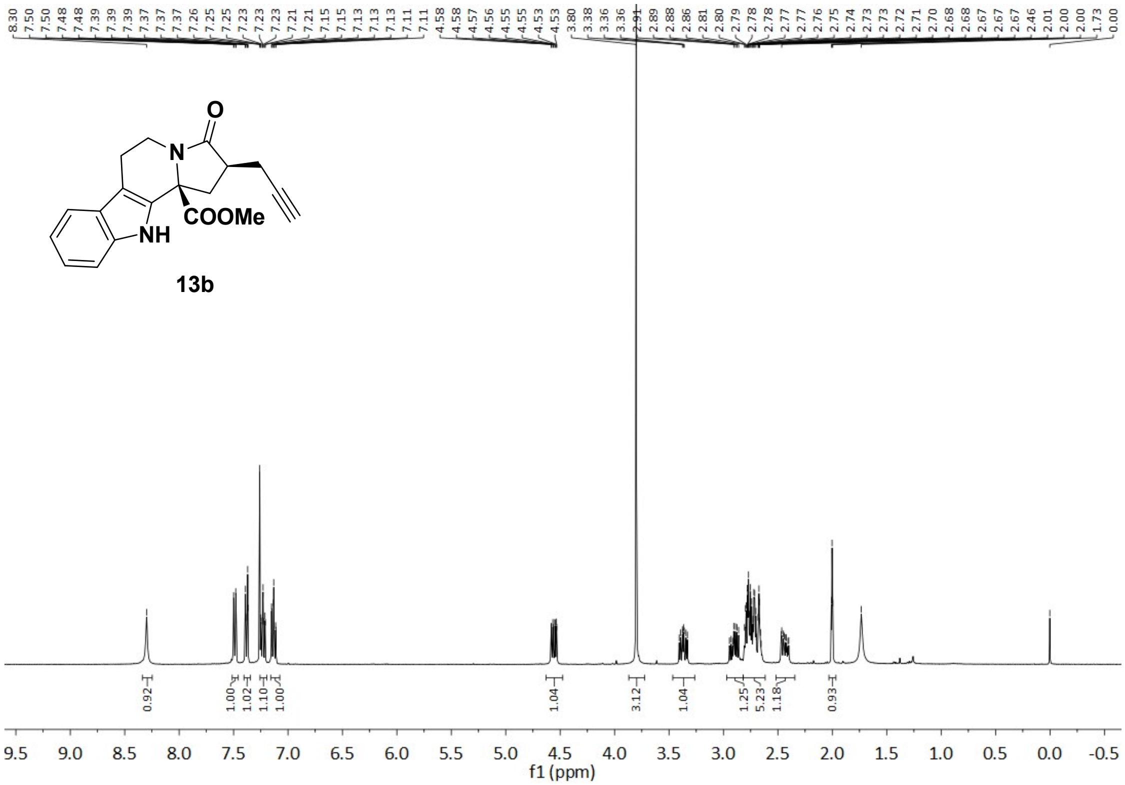

^{1}H NMR spectrum of compound **13b**

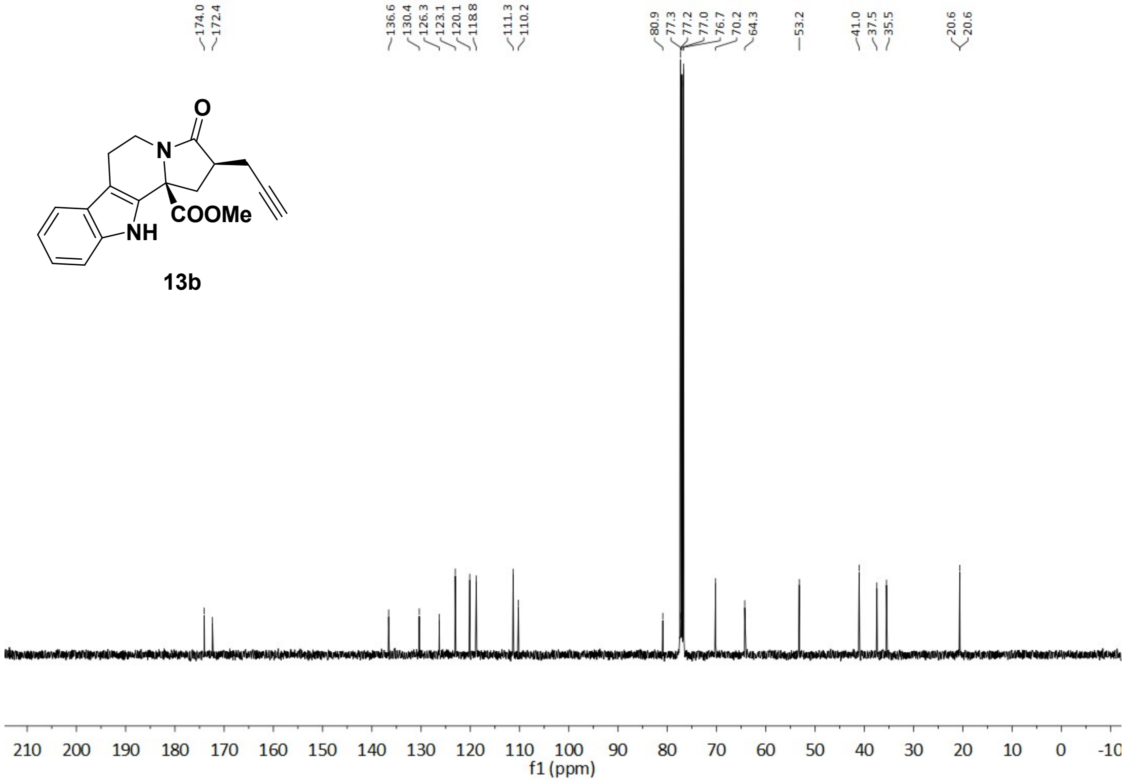

^{13}C NMR spectrum of compound **13b**

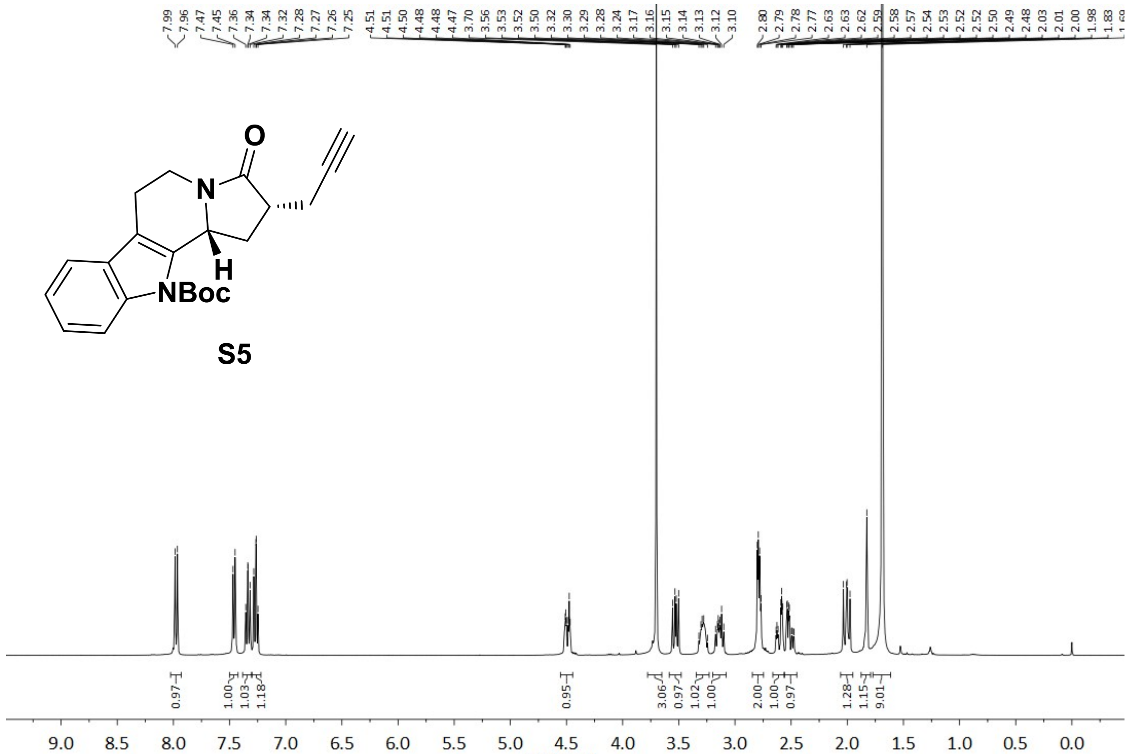

^{1}H NMR spectrum of compound **S5**

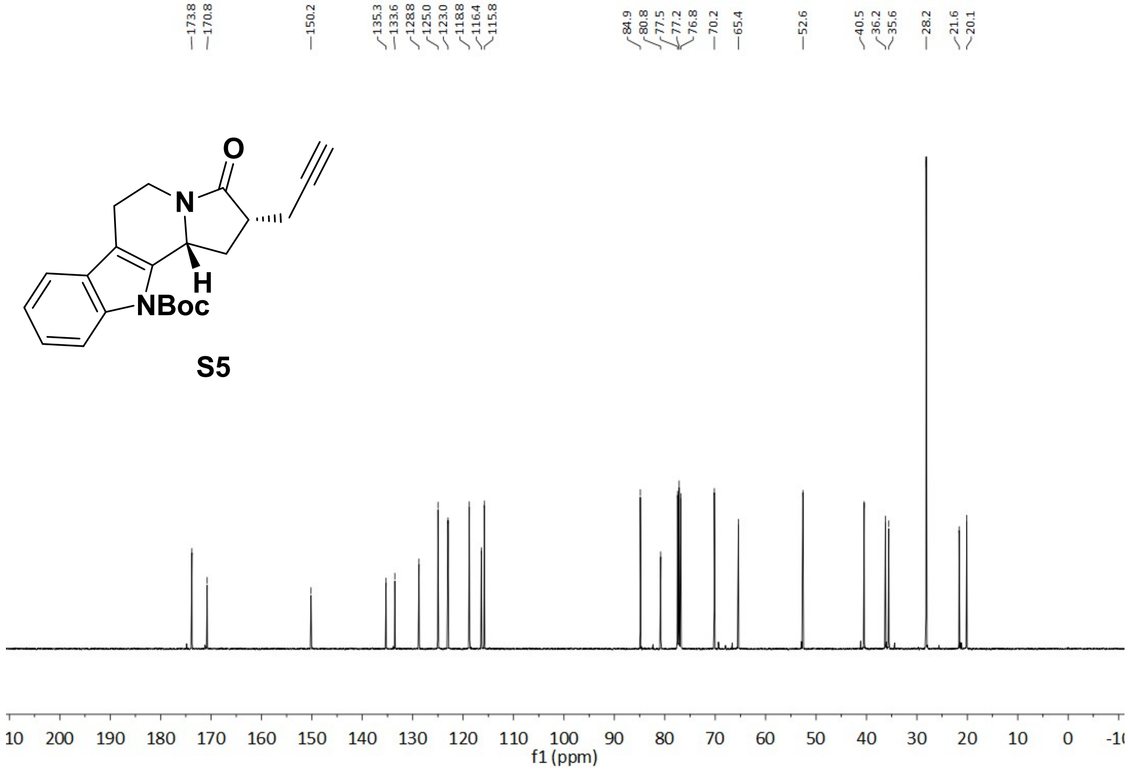

^{13}C NMR spectrum of compound **S5**

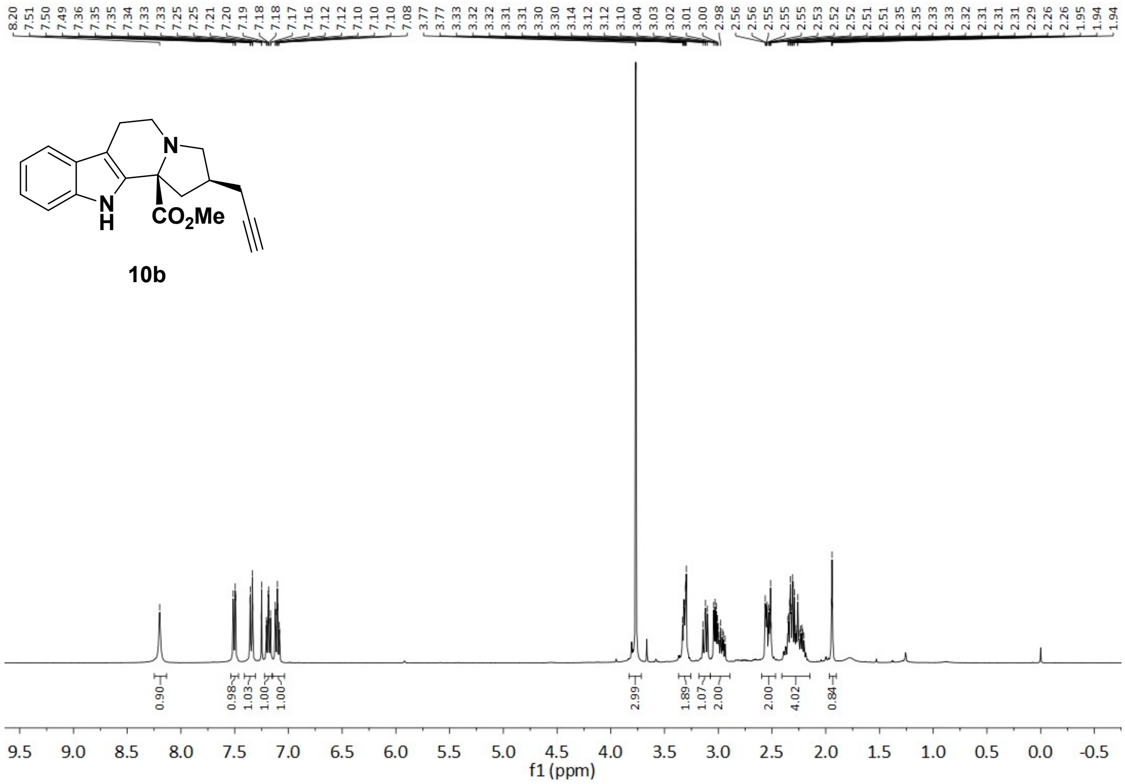

^{1}H NMR spectrum of compound **10b**

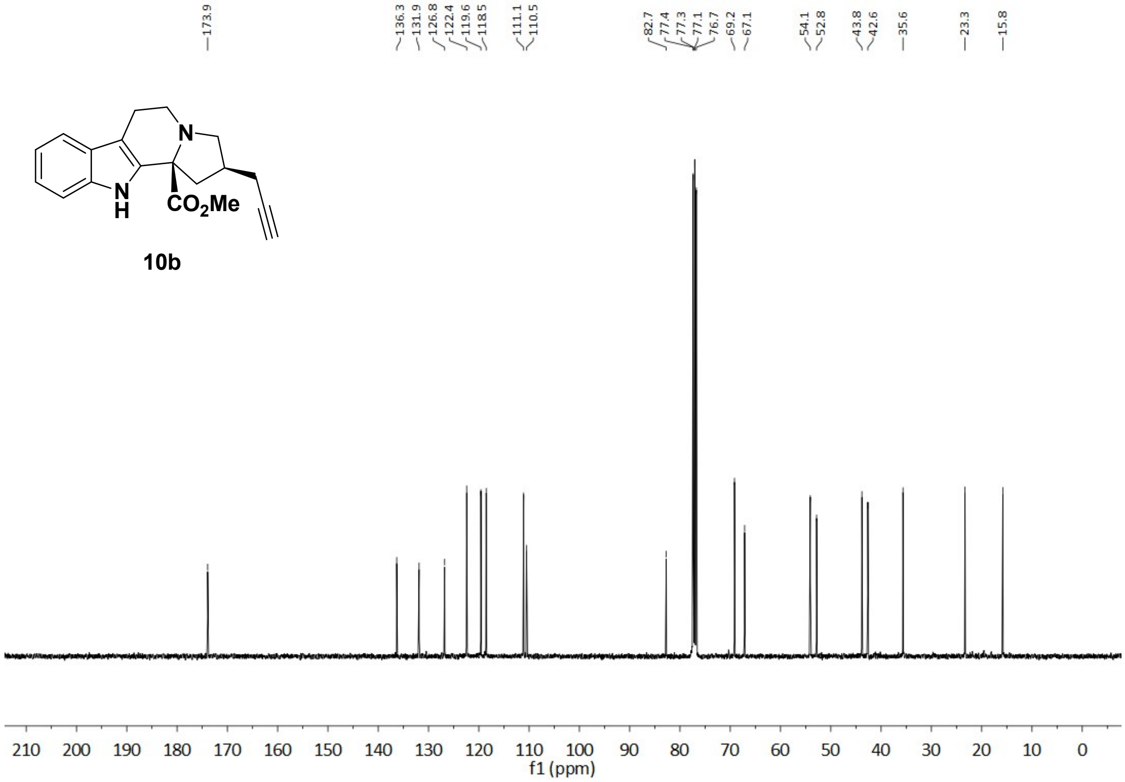

^{13}C NMR spectrum of compound **10b**

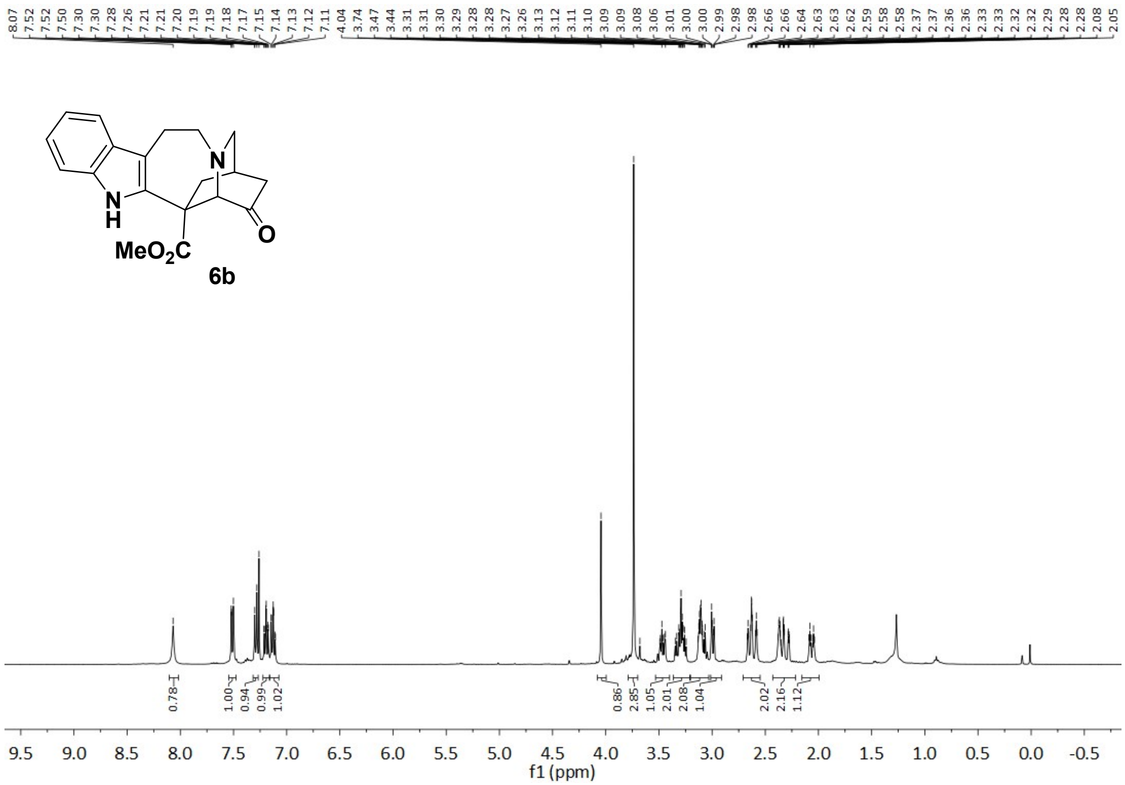

^{1}H NMR spectrum of compound **6b**

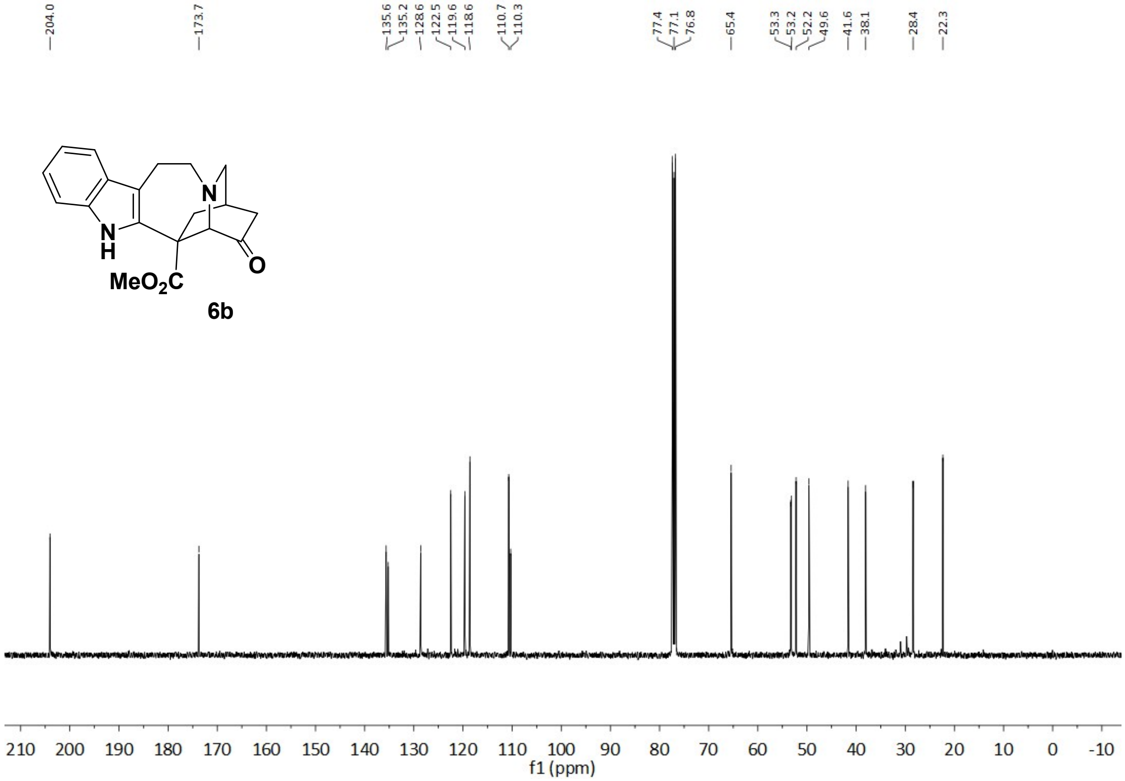

^{13}C NMR spectrum of compound **6b**

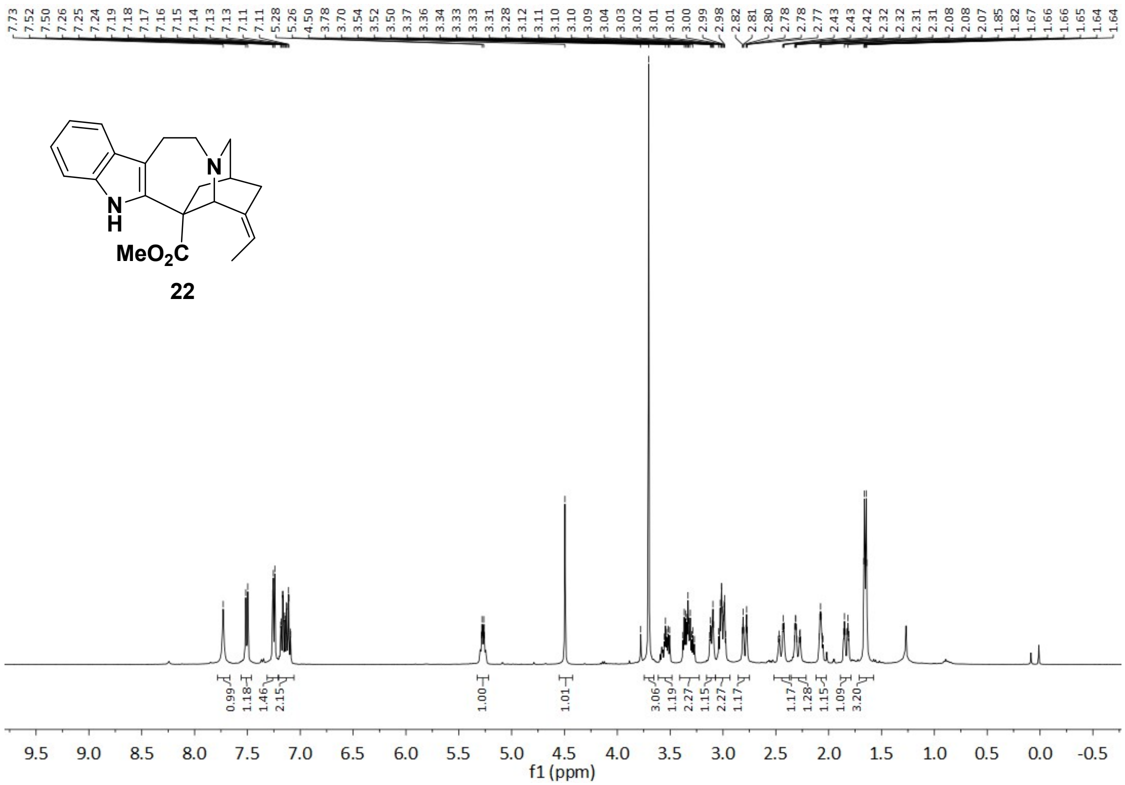

^{1}H NMR spectrum of compound **22**

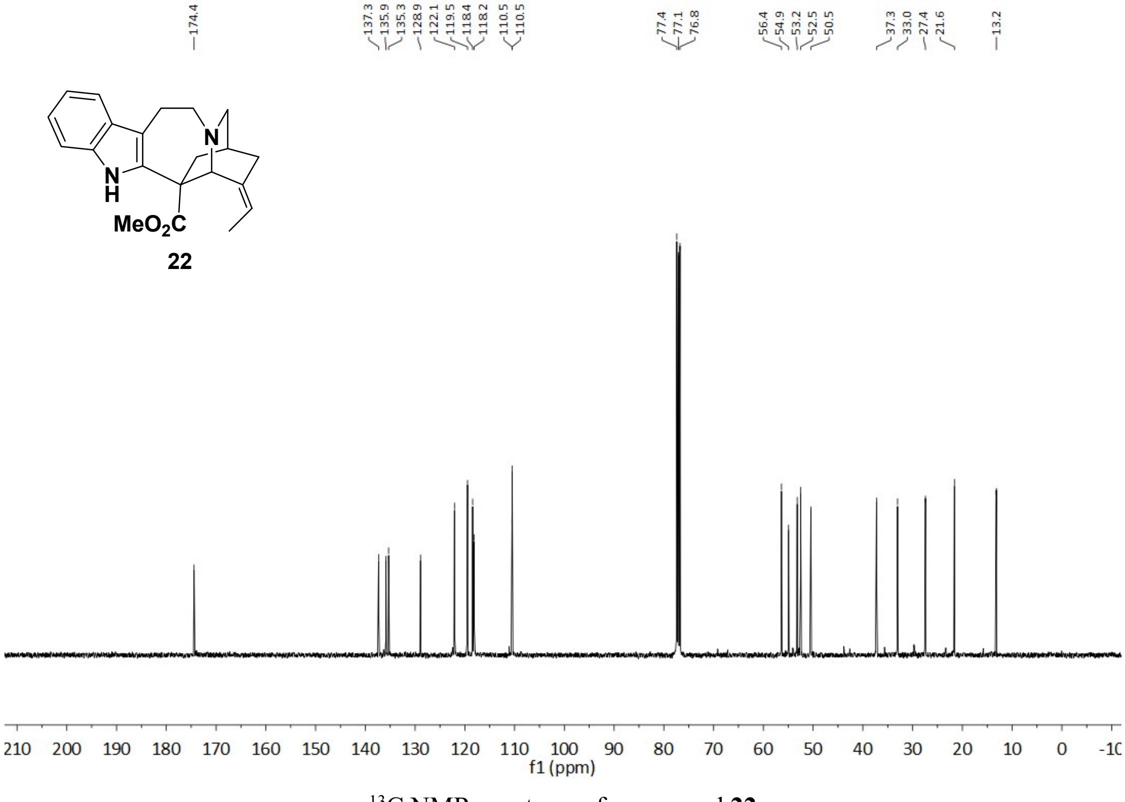

^{13}C NMR spectrum of compound **22**

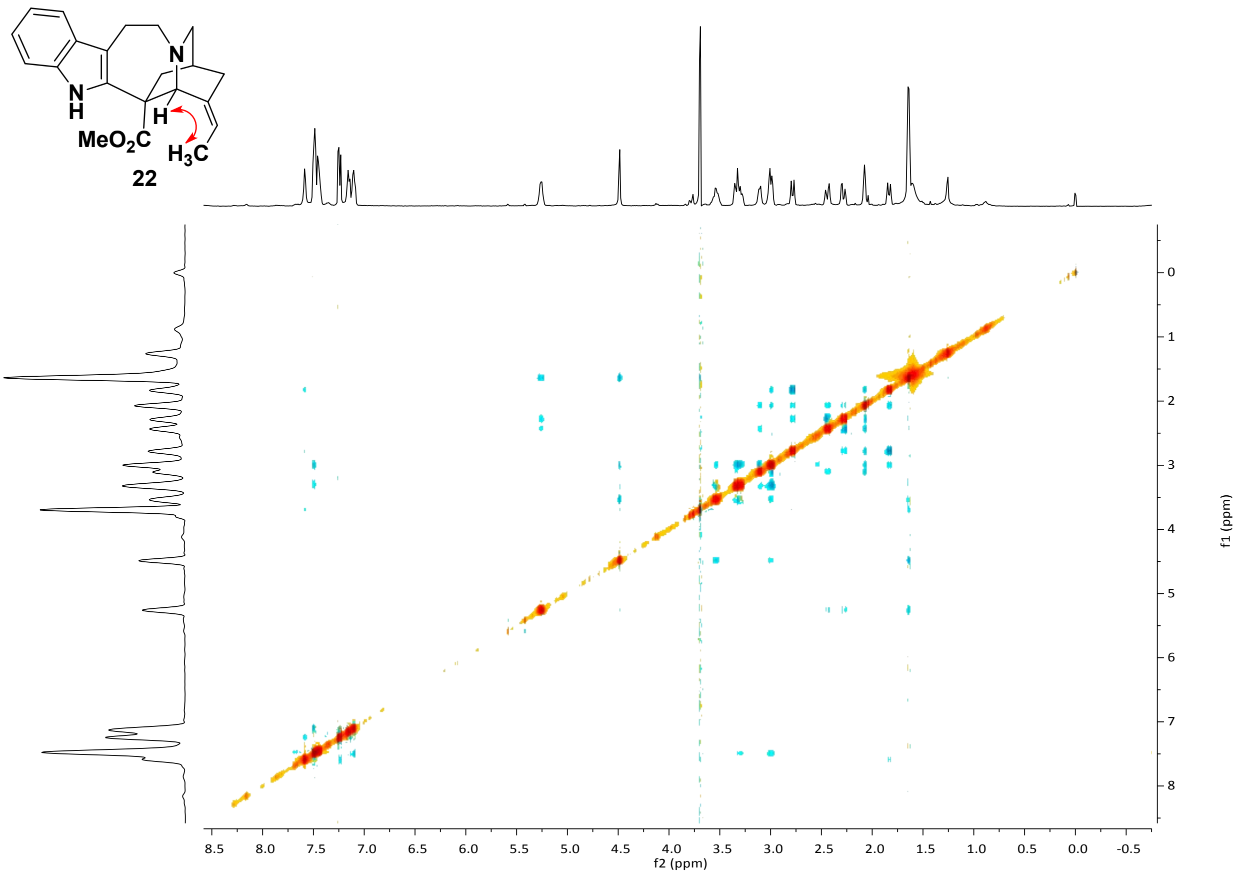

^{1}H-^{1}H NOESY spectrum of compound **22**

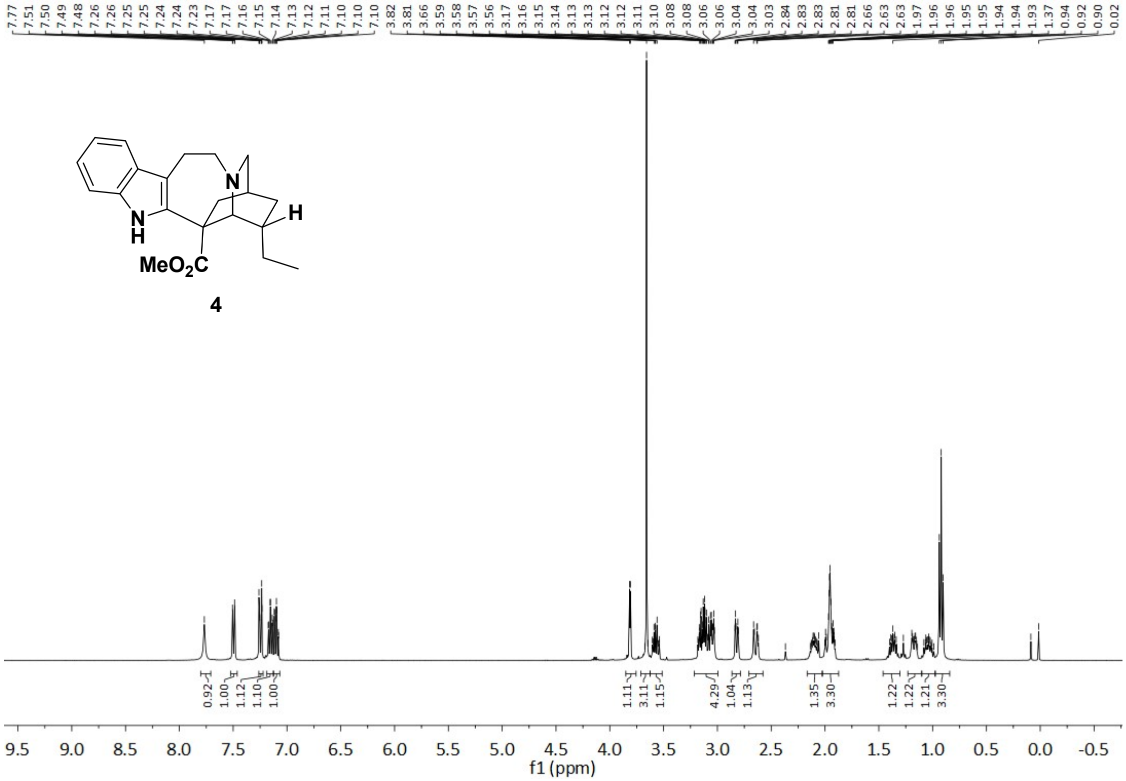

^{1}H NMR spectrum of compound **4**

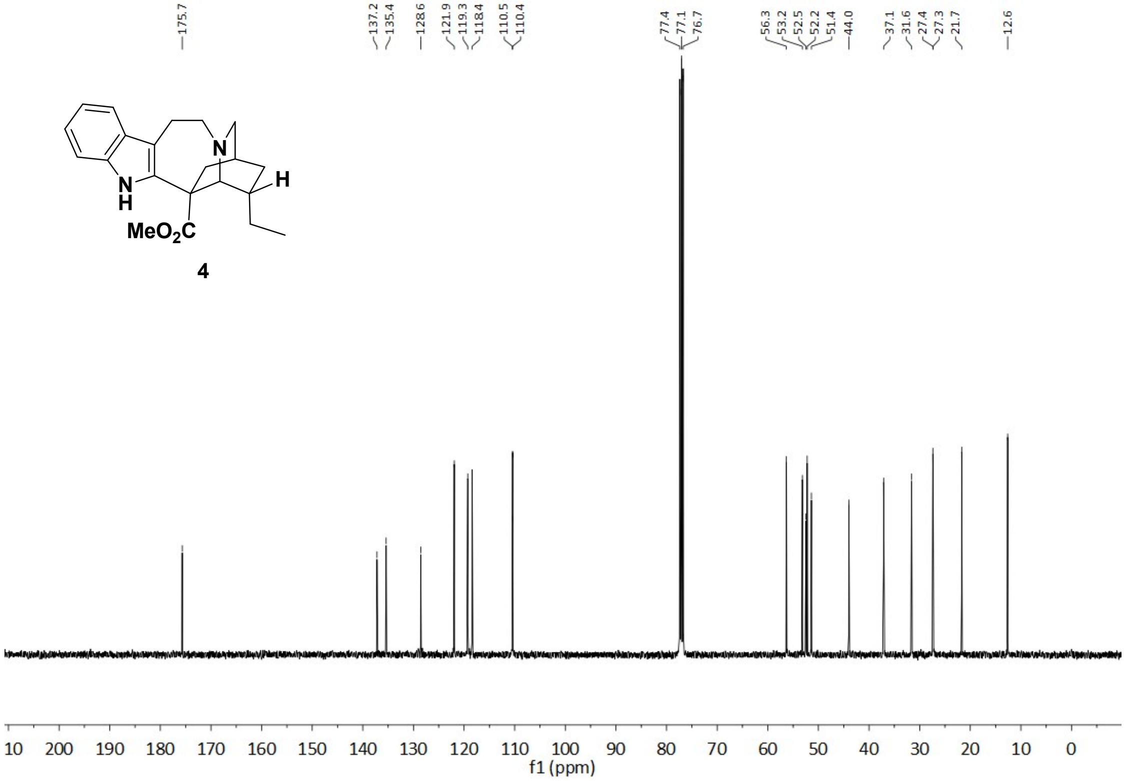

^{13}C NMR spectrum of compound **4**

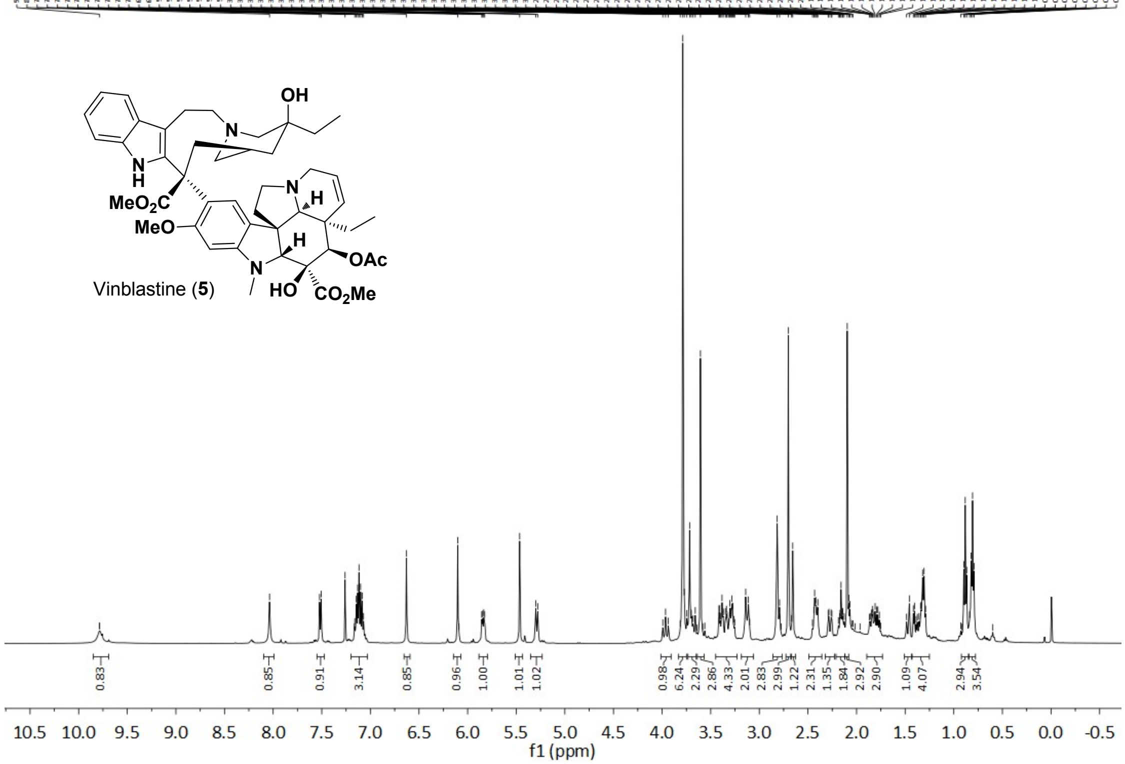

^{1}H NMR spectrum of compound **5**

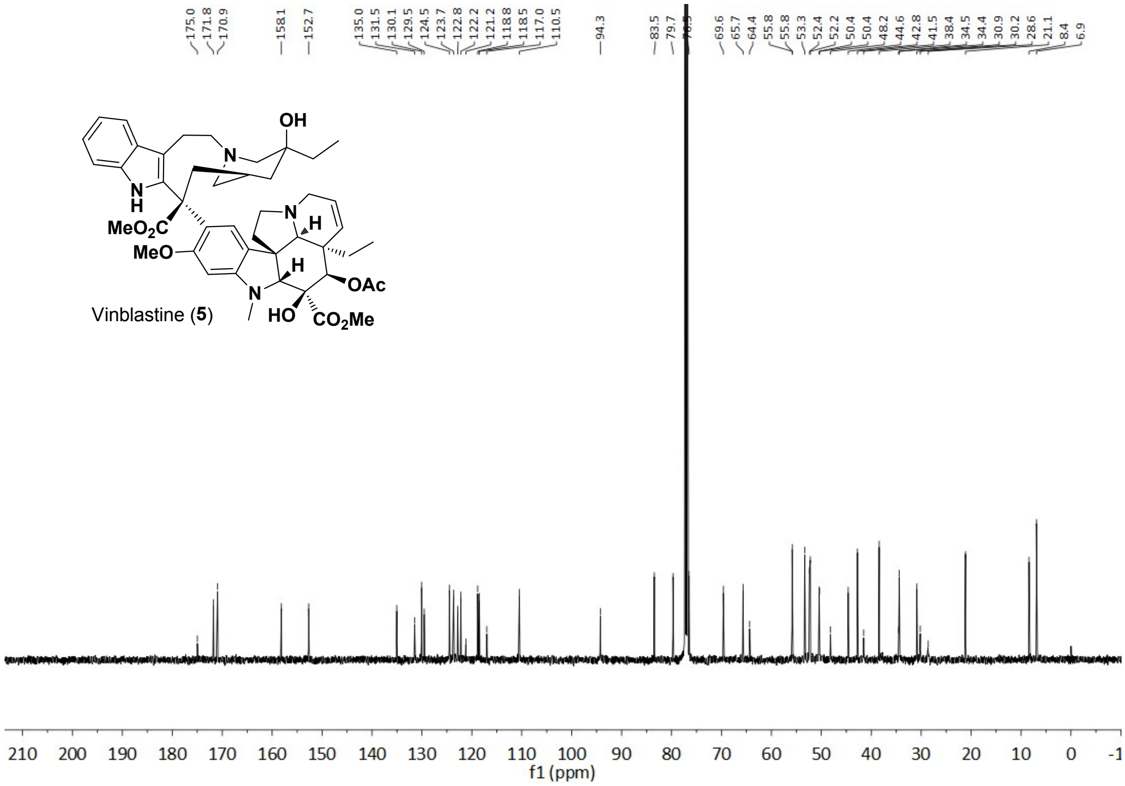

^{13}C NMR spectrum of compound **5**

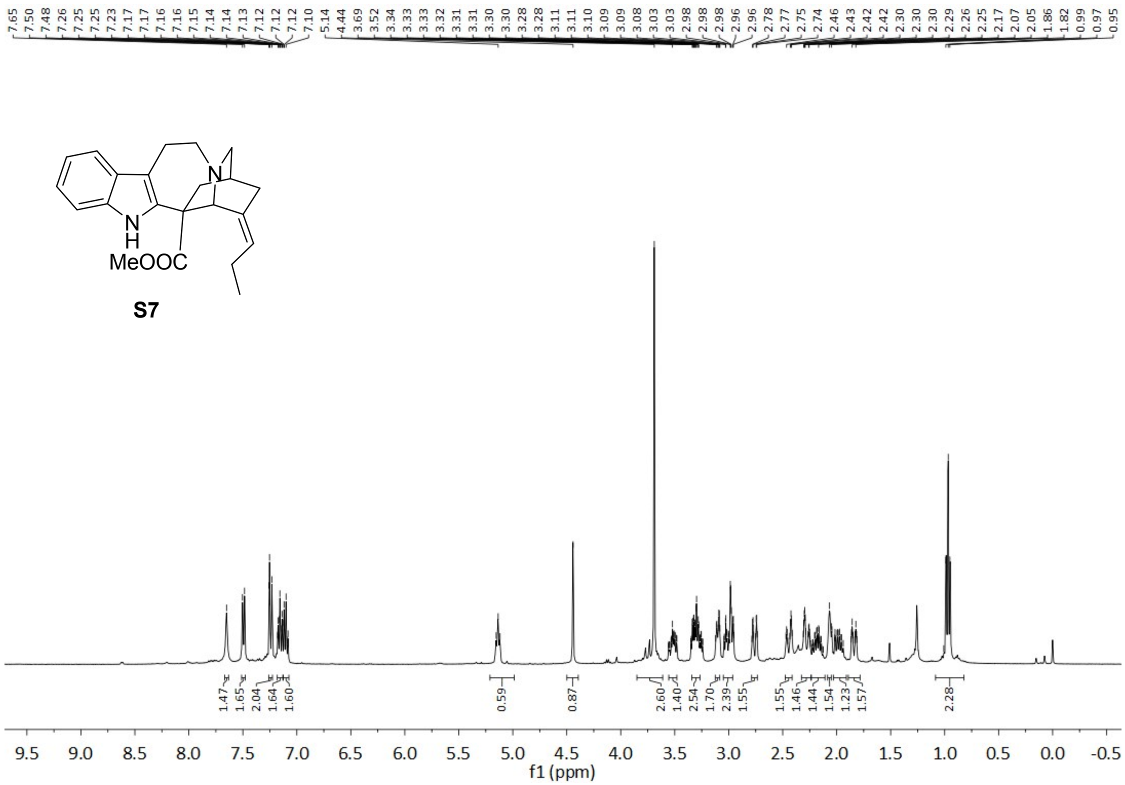

^{1}H NMR spectrum of compound **S7**

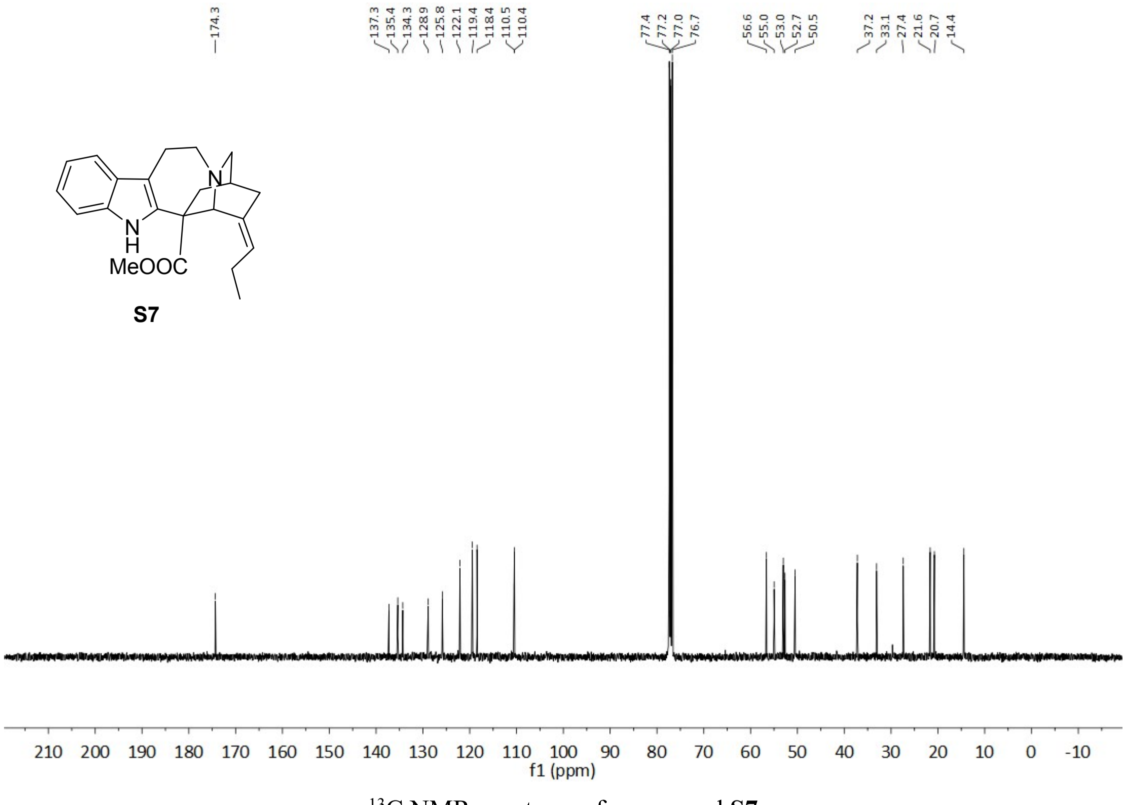

^{13}C NMR spectrum of compound **S7**

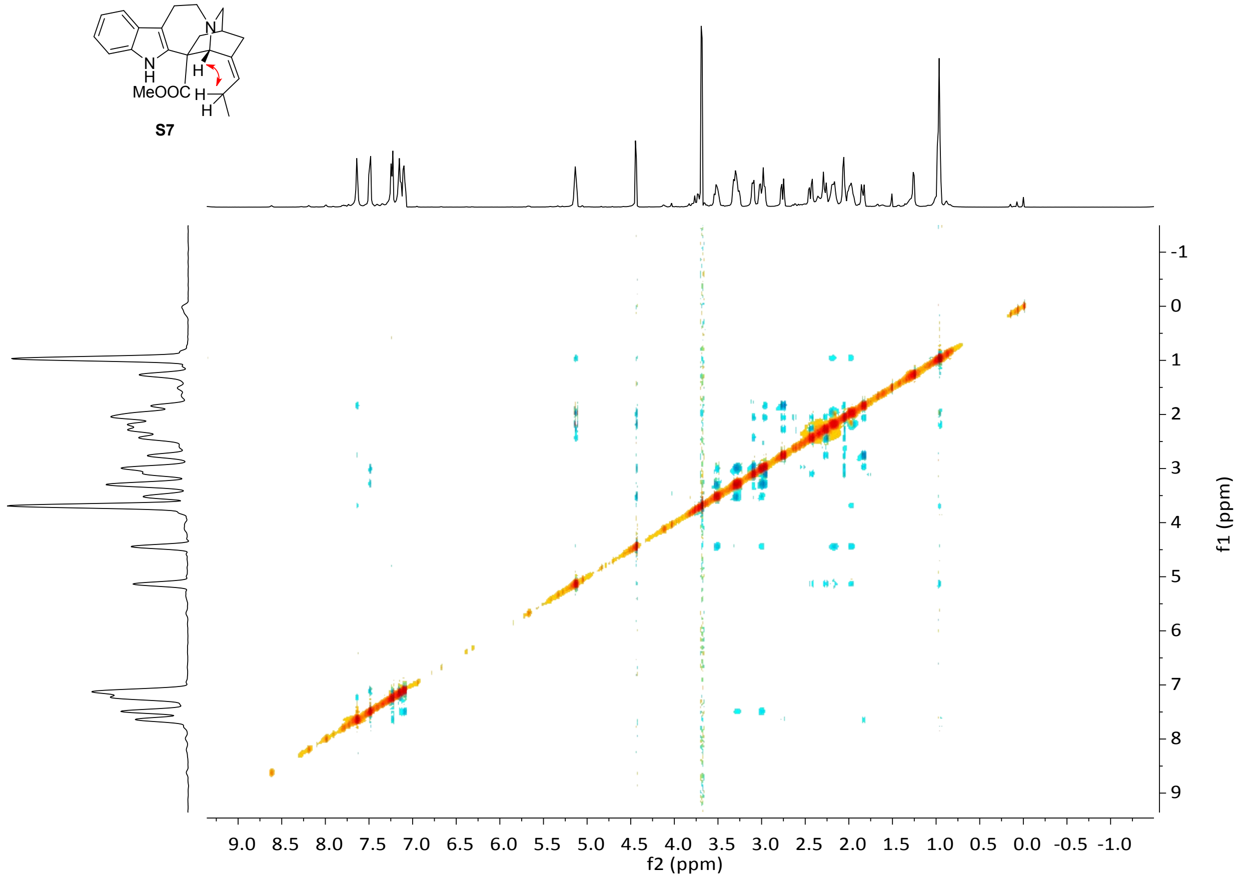

^{1}H-^{1}H NOESY spectrum of compound **S7**

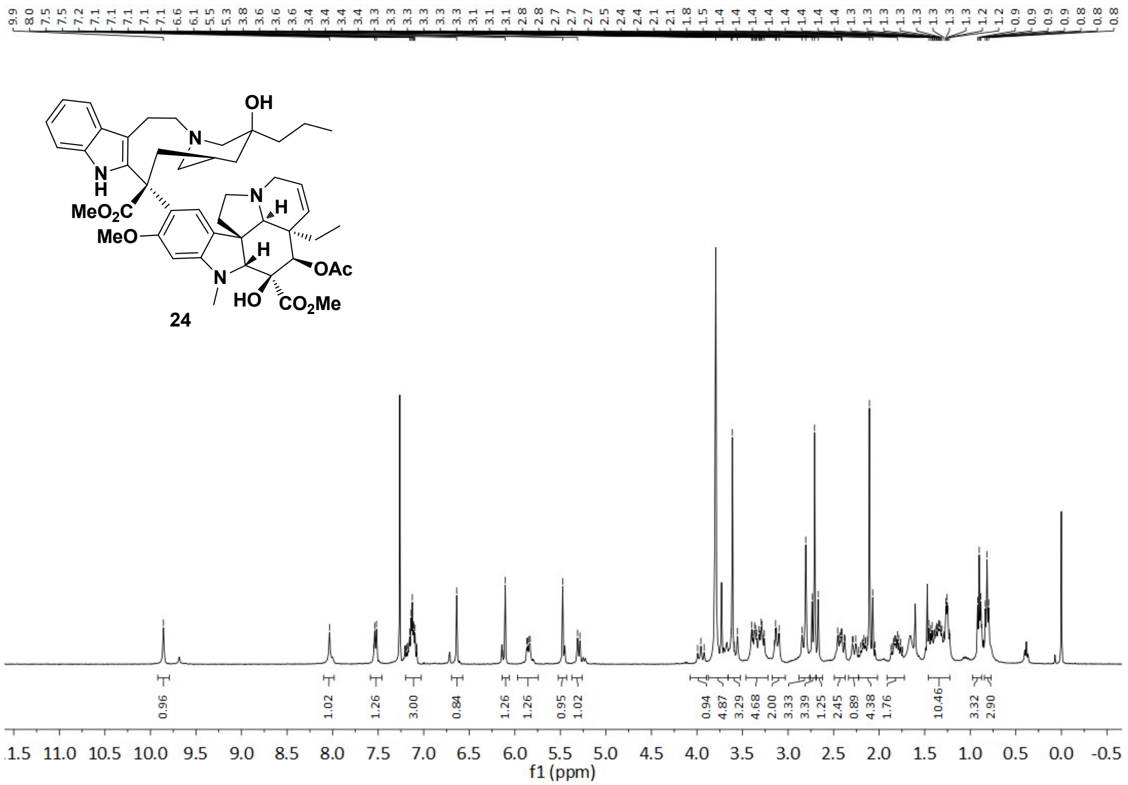

^{1}H NMR spectrum of compound **24**

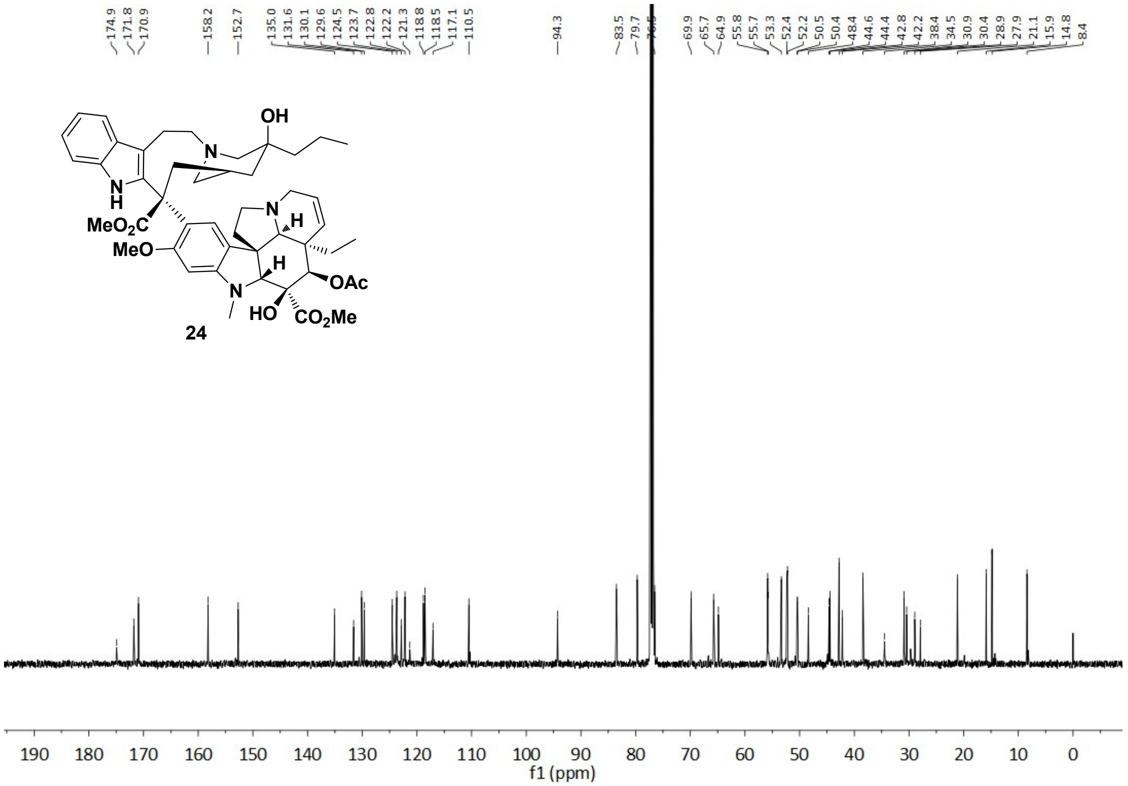

^{13}C NMR spectrum of compound **24**

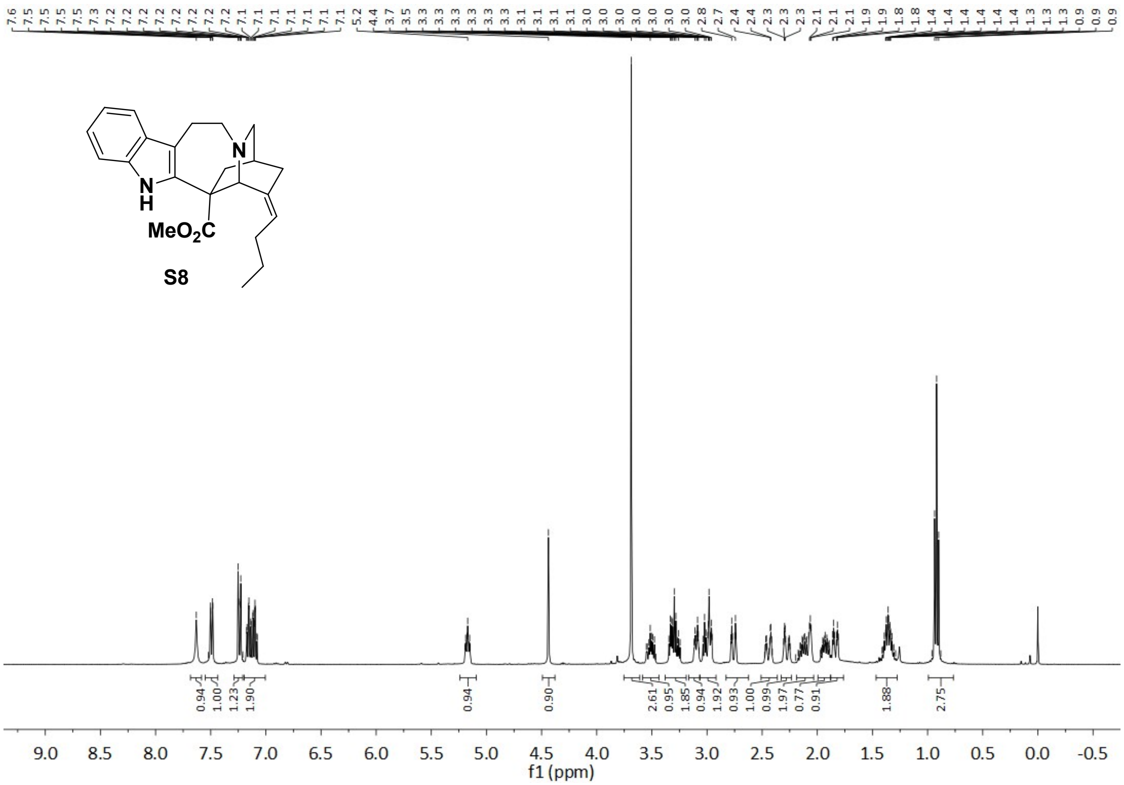

^{1}H NMR spectrum of compound **S8**

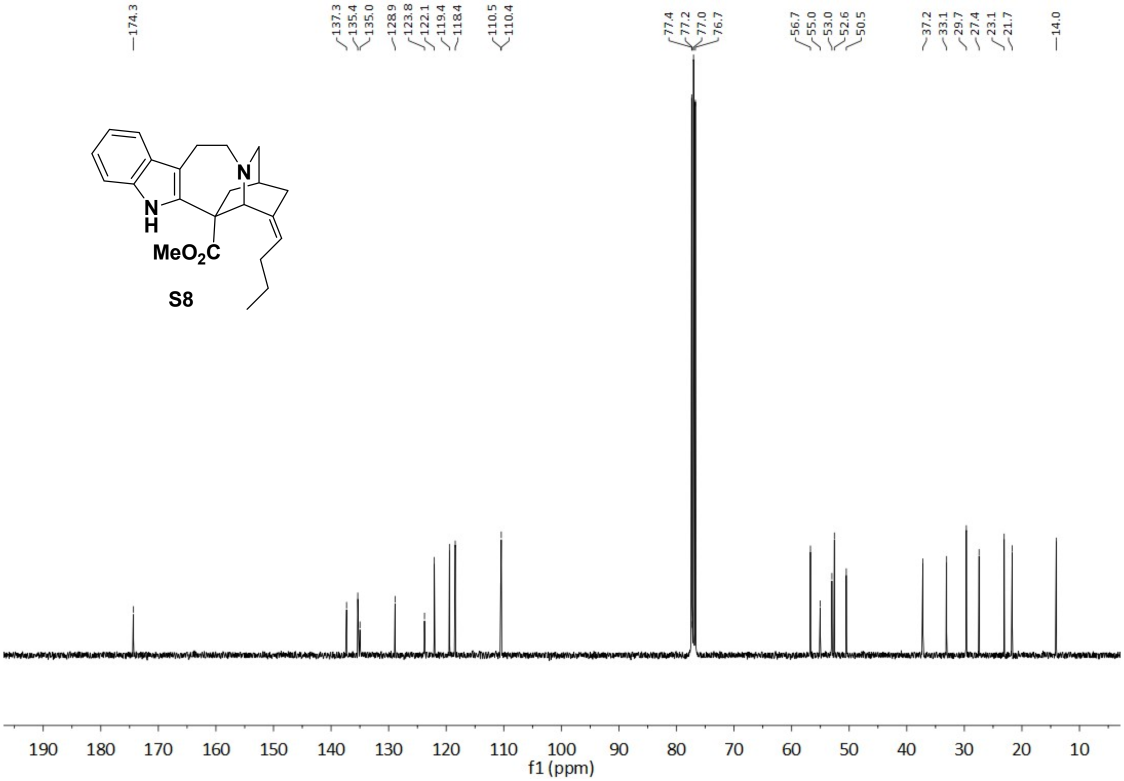

^{13}C NMR spectrum of compound **S8**

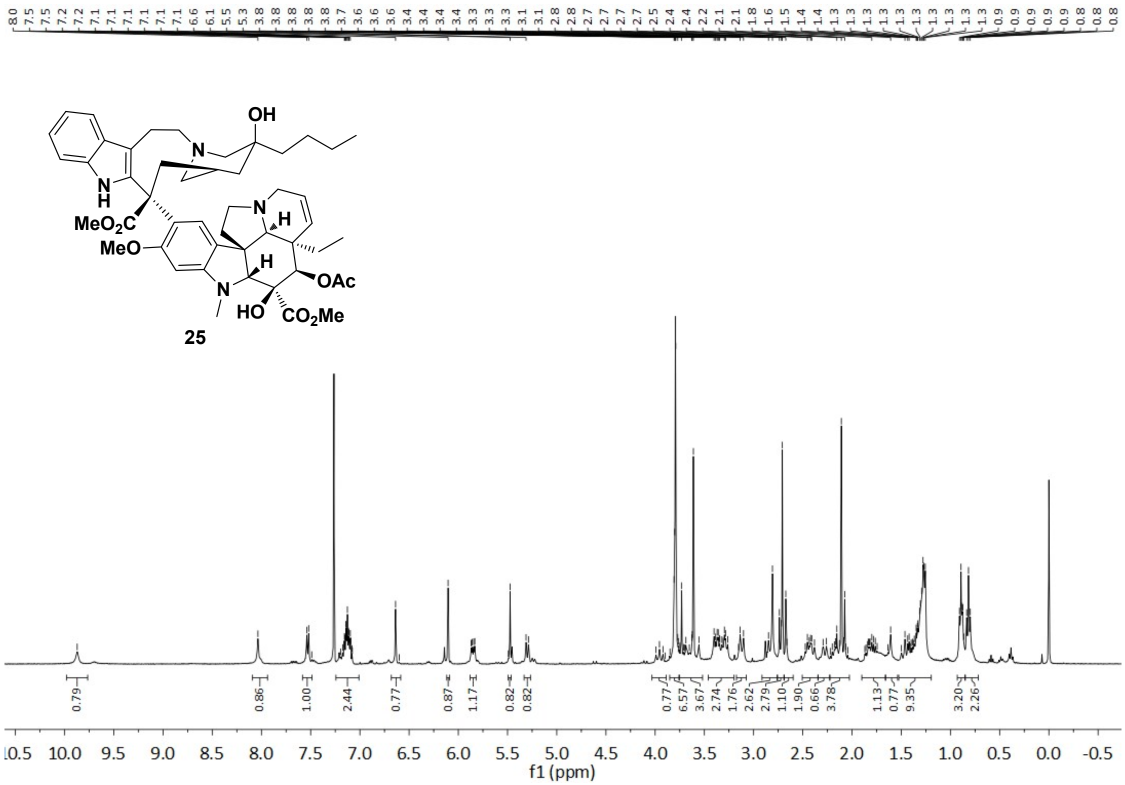

^{1}H NMR spectrum of compound **25**

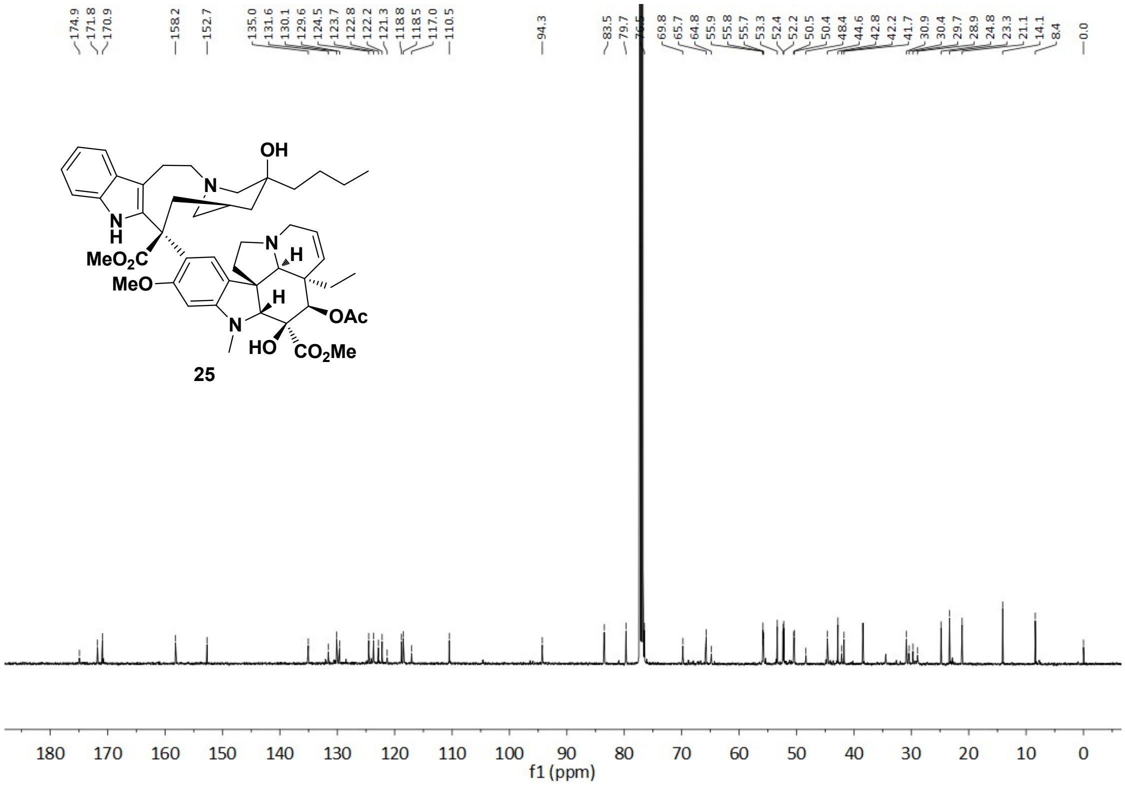

^{13}C NMR spectrum of compound **25**

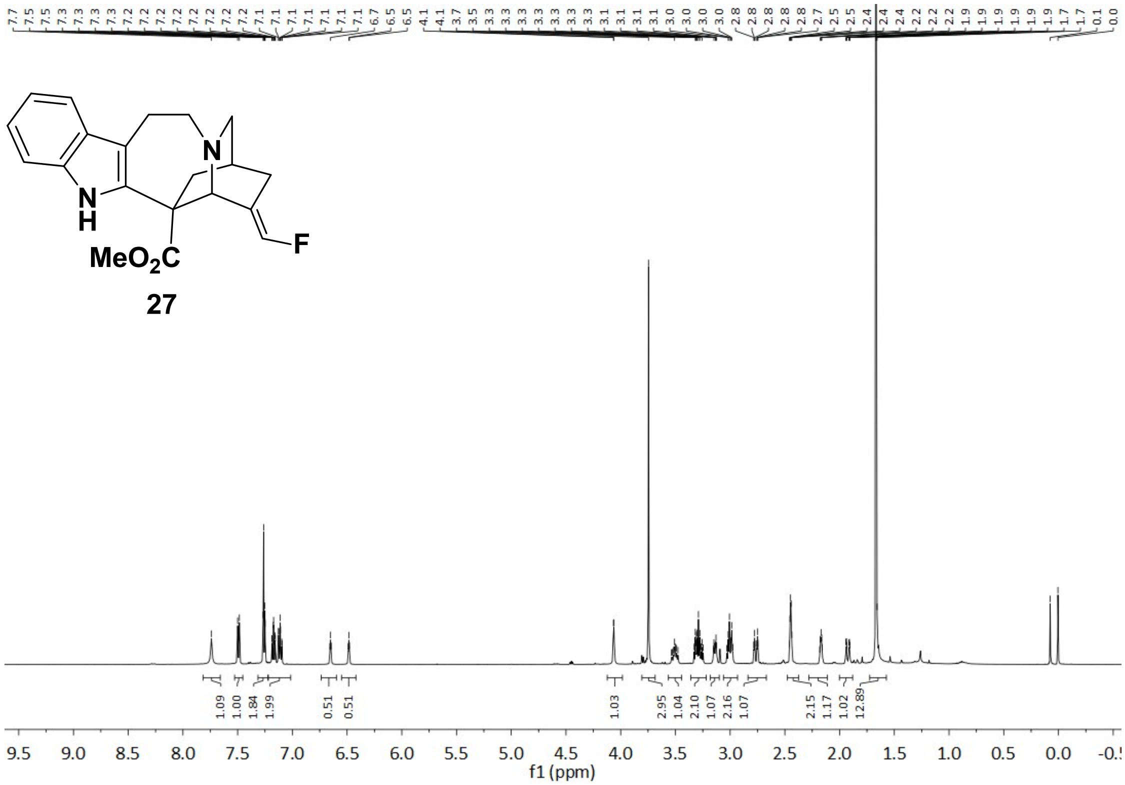

^{1}H NMR spectrum of compound **27**

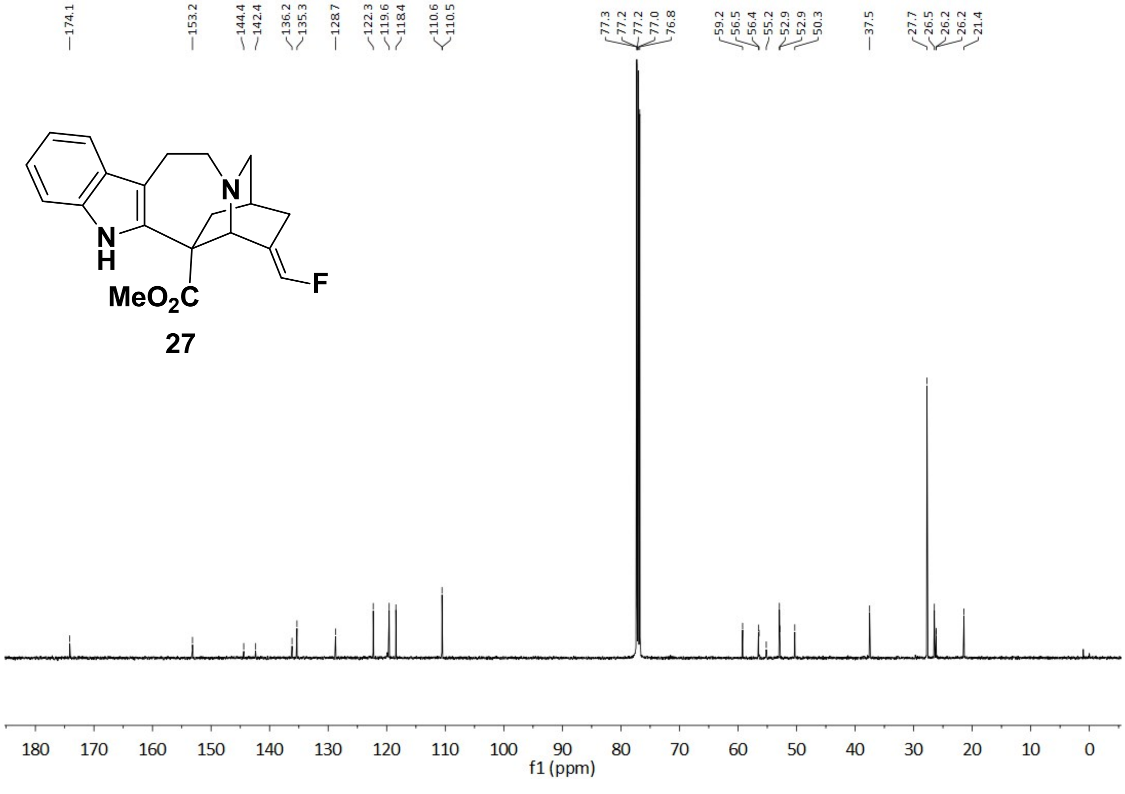

^{13}C NMR spectrum of compound **27**

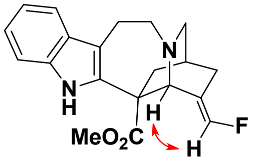

27

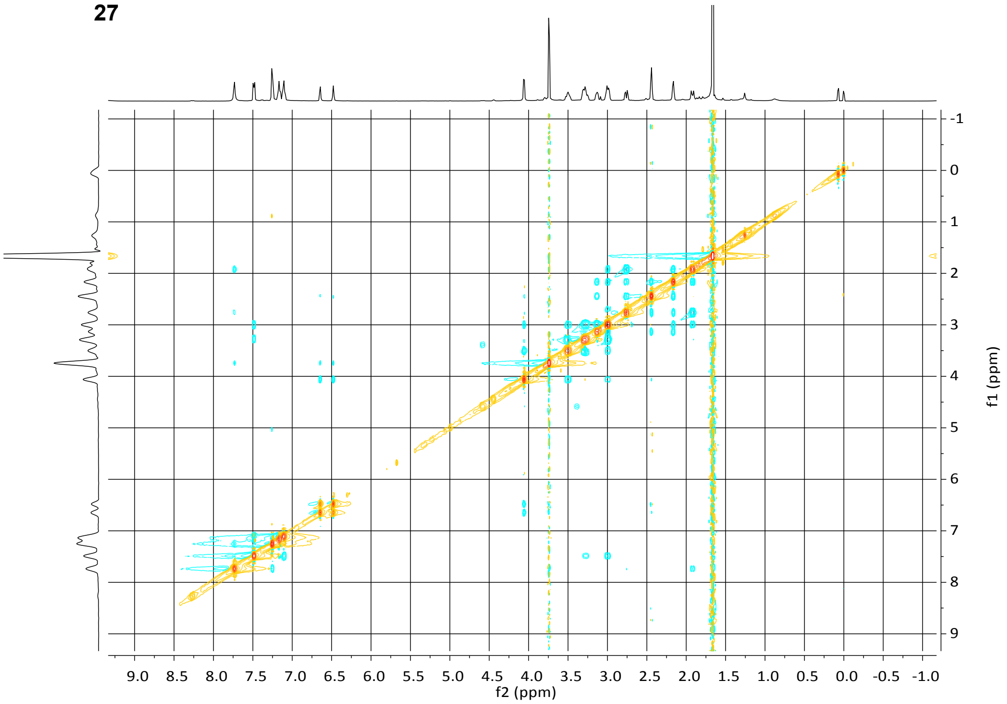

^{1}H-^{1}H NOESY spectrum of compound **27**

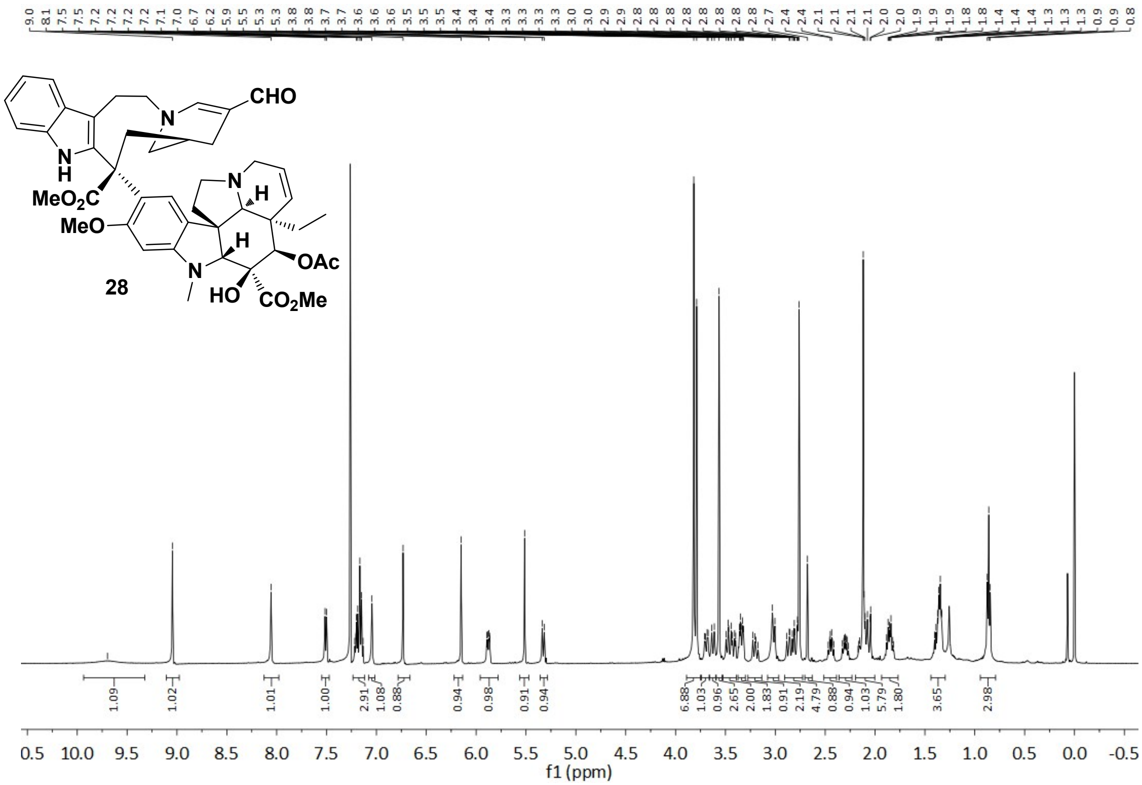

^{1}H NMR spectrum of compound **28**

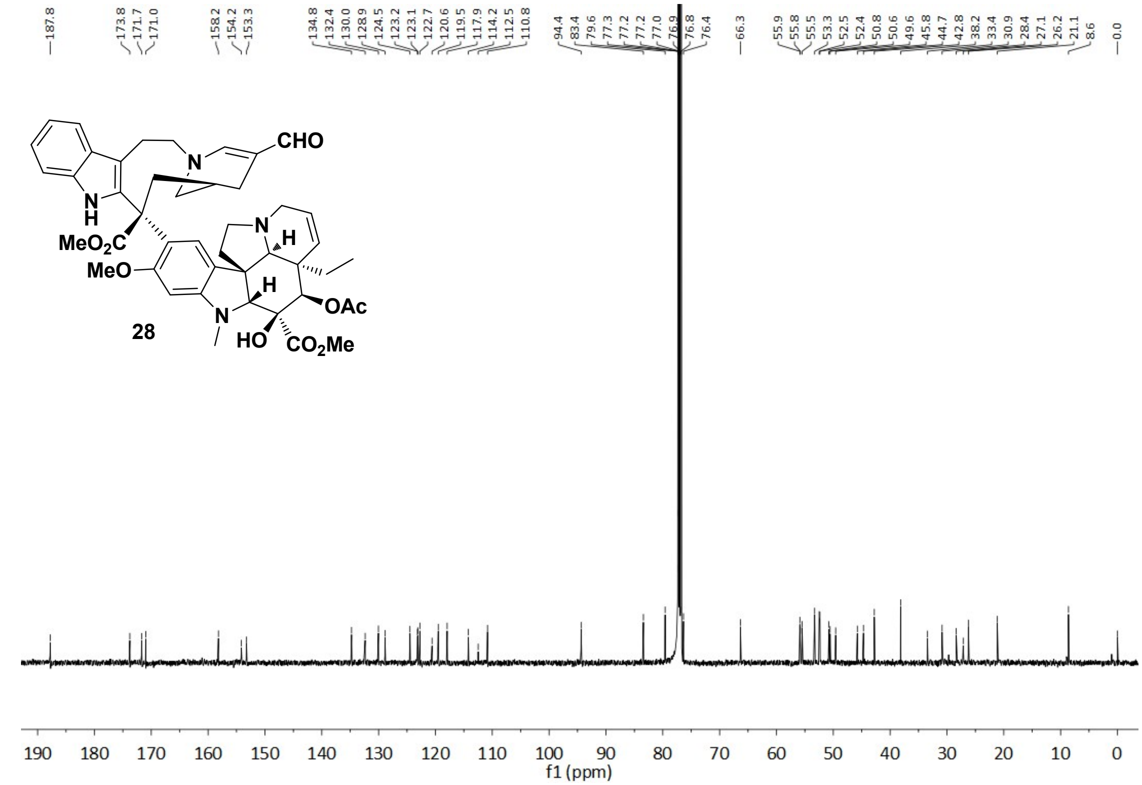

^{13}C NMR spectrum of compound **28**

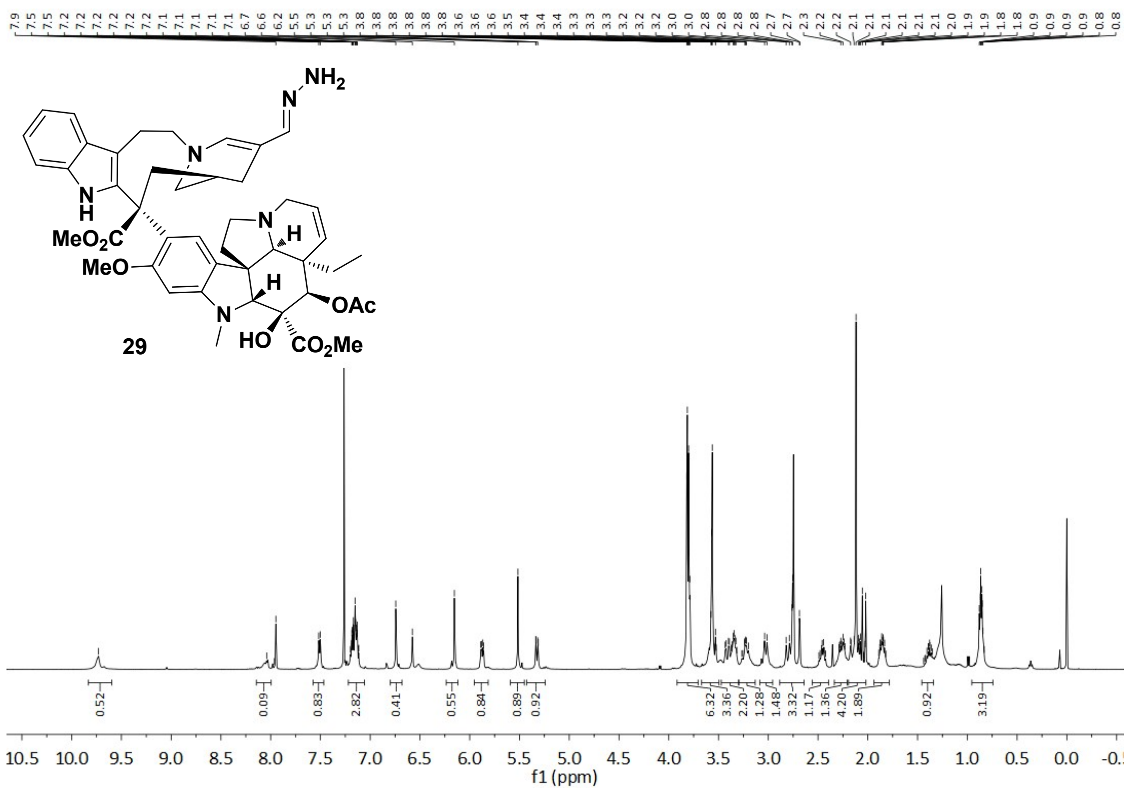

^1H NMR spectrum of compound **29**

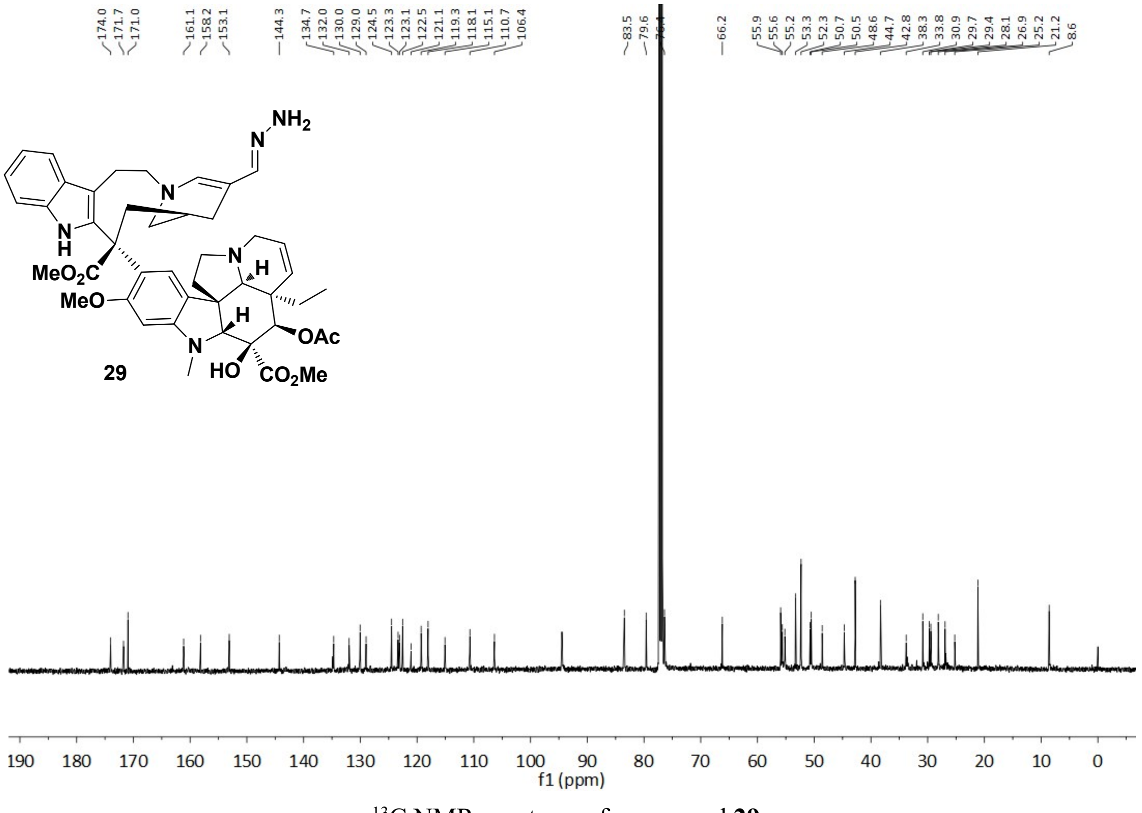

^{13}C NMR spectrum of compound **29**

VI: References

[1] L. Moisan; P. Thuéry; M.Nicolas; E. Doris; B. Rousseau, *Angew. Chem., Int. Ed.* 2006, **45**, 5334-5336.

[2] D. M. Hodgson; J. Galano, *Org. Lett.* 2005, **7**, 2221-2224.

[3] J. W. Beatty; C. R. J. Stephenson, *J. Am. Chem. Soc.* 2014, **136**, 10270-10273.

[4] H. Ishikawa; D. A. Colby; S. Seto; P. Va; A. Tam; H. Kakei; T. J. Rayl; I. Hwang; D. L. Boger, *J. Am. Chem. Soc.* 2009, **131**, 4904-4916.

[5] F. Y. F. Lee; R. Borzilleri; C. R. Fairchild; S.-H. Kim; B. H. Long; C. Revetos-Suatez; G. D. Vite; W. C. Rose; R. A. Kramer, *Clin. Cancer Res.* 2001, **7**, 1492-1437.

[6] E. Giovanelli, L. Moisan, S. Leroux, S. Comesse, B. Rousseau, P. Hellier, M. Nicolas and E. Doris, *Chem. Eur. J.*, 2013, **19**, 1170-1173.
